# TGF-**β** serves as a critical signaling determinant of liver progenitor cell activation and function

**DOI:** 10.1172/JCI199913

**Published:** 2026-07-28

**Authors:** Chenhao Tong, Tao Lin, Han Wang, Luyao Jiang, Xiaodong Yuan, Wenwu Luo, Minghan Zhou, Carolina De La Torre, Hui Liu, Chen Shao, Seddik Hammad, Hui Gao, Jiarong Xie, Lei Xu, Roman Liebe, Zuguang Gu, Matthias P. Ebert, Huiguo Ding, Steven Dooley, Hong-Lei Weng

**Affiliations:** 1 Department of Medicine II, Section Molecular Hepatology, University Medical Center Mannheim, Medical Faculty Mannheim, Heidelberg University, Mannheim, Germany.; 2 Hepatopancreatobiliary Surgery Department, The First Affiliated Hospital of Ningbo University, Ningbo, Zhejiang, China.; 3 Department of Biochemistry, Case Western Reserve University, Cleveland, Ohio, USA.; 4 Liver Disease Research Laboratory, The First Affiliated Hospital of Ningbo University, Ningbo, Zhejiang, China.; 5 Department of General Surgery, The First Affiliated Hospital of USTC, Division of Life Sciences and Medicine, University of Science and Technology of China, Hefei, Anhui, China.; 6 Department of Pathology, The First Affiliated Hospital of Anhui Medical University, Anhui, China.; 7 NGS Core Facility, Medical Faculty Mannheim, University of Heidelberg, Mannheim, Germany.; 8 Department of Pathology, Beijing You’an Hospital affiliated with Capital Medical University, Beijing, China.; 9 Department of Gastroenterology, The First Affiliated Hospital of Ningbo University, Ningbo, China.; 10 Department of Gastroenterology, Hepatology and Transplant Medicine, Medical Faculty, University of Duisburg-Essen, Essen, Germany.; 11 Institute of Cell and Gene Technology, Shenzhen University of Advanced Technology, Shenzhen, China.; 12 Computational Oncology Group, Molecular Precision Oncology Program, National Center for Tumor Diseases (NCT) Heidelberg and German Cancer Research Center (DKFZ), Heidelberg, Germany.; 13 Department of Medicine II, University Medical Center Mannheim, Medical Faculty Mannheim, Heidelberg University, Mannheim, Germany.; 14 DKFZ-Hector Cancer Institute at the University Medical Center Mannheim, Mannheim, Germany.; 15 Molecular Medicine Partnership Unit, European Molecular Biology Laboratory, Heidelberg, Germany.; 16 Department of Gastroenterology and Hepatology, Beijing You’an Hospital affiliated with Capital Medical University, Beijing, China.

**Keywords:** Cell biology, Hepatology, Adult stem cells, Cell cycle

## Abstract

When massive hepatic necrosis–associated (MHN-associated) acute liver failure (ALF) occurs following severe damage, liver progenitor cells (LPCs) exit quiescence and enter differentiation programs during which they acquire hepatocyte-like functions. How LPCs maintain quiescence under physiological conditions and orchestrate activation following MHN remains largely unknown. We elucidate an essential role of TGF-β in regulating LPC quiescence and activation. Spatial transcriptomics and single-cell sequencing revealed that LPCs receive multiple signals, particularly TGF-β, HGF, and EGF, from surrounding hepatic stellate cells and macrophages in patients and zebrafish with MHN-induced ALF. Physiologically, TGF-β inhibits LPC proliferation by blocking G1-to-S phase transition, an effect reversed by *Smad7* overexpression in a murine injury model. We observed extensive LPC proliferation in patients with ALF despite strong TGF-β/phosphorylated SMAD signaling. Immunostaining revealed concurrent activation of HGF/MET, EGF/EGFR, and downstream STAT3/ERK pathways in LPCs. In vitro, HGF or EGF overcame TGF-β–mediated growth arrest and promoted LPC proliferation. Beyond acting as a mitogen, HGF additionally induced hepatocyte gene programs (e.g., *Hnf4a*, *Hnf1a*) in LPCs. Strikingly, TGF-β signaling was required for HGF-dependent hepatocyte gene induction, indicating a dual role in restraining LPC proliferation and promoting functional maturation. These findings position TGF-β as a context-dependent determinant of LPC activation and lineage specification during ALF.

## Abstract 2

In acute liver failure, growth signals override TGF-β’s brake so the organ’s backup repair cells multiply—yet TGF-β remains essential for turning them into working liver cells.

## Introduction

Acute liver failure (ALF) results from 2 catastrophic events in the liver: (a) loss of the majority of the parenchyma, massive hepatic necrosis (MHN), or submassive hepatic necrosis and (b) metabolic dysfunction in hepatocytes caused by mitochondrial toxicity (1–5). MHN is the most severe pathologic lesion in ALF (6–8). In MHN-associated ALF, liver progenitor cells (LPCs), the smallest cholangiocytes, exit quiescence and acquire hepatocyte-like functions to support systemic homeostasis (3, 5, 9–12). Over time, activated LPCs differentiate into mature hepatocytes and restore lost liver mass (12), a process termed the second pathway of liver regeneration (13). Detailed mechanisms of how LPCs rapidly proliferate, perform hepatic function, and subsequently differentiate into hepatocytes remain largely unknown.

Physiologically, quiescent LPCs reside in the canal of Hering and the smallest branches of the biliary tree (9). Inflammatory mediators in liver disease activate LPCs to give rise to ductular reaction (DR), defined as a reaction of ductular phenotype, possibly but not necessarily of ductular origin (9). Depending on the disease settings, DR may arise from distinct sources, including proliferation of preexisting cholangiocytes, activation of LPCs, or rarely, biliary metaplasia of hepatocytes (14). In MHN-associated ALF, LPCs are the predominant source of DR because most parenchymal cells are destroyed (14). There are 3 major open questions in LPC biology: (a) How are LPCs maintained in a quiescent state in normal liver? (b) Which microenvironment-derived signals, such as inflammatory mediators, induce LPC proliferation? (c) How do these external signals drive LPC differentiation toward hepatocytes? Among multiple inflammatory cytokines known to regulate LPC activation in various liver diseases and animal models (15–21), the effects of TGF-β on LPC biology warrant deeper investigation.

Members of the TGF-β superfamily play fundamental roles in development, tissue homeostasis, and regeneration (22). TGF-β signaling is considered a guardian in the preservation of systemic, tissue, and cellular integrity (23). In many normal cell types, TGF-β has the last word in determining whether cells proliferate (24). In the context of cell cycle control, the most important targets of TGF-β are *CDKN1A* and *CDKN2B* genes, which encode the CDK inhibitors p21^Cip1^ and p15^INK4B^, respectively (25, 26). In this process, transcription factors FOXO1/3/4, C/EBP-β, and MIZ-1 cooperate with SMAD3-SMAD4 complexes to activate transcription of p15^INK4B^ and p21^Cip1^ (25, 26). In addition, C/EBP-β synergizes with SMAD proteins to repress c-MYC, further contributing to growth inhibition (26). In normal rat livers, inhibition of TGF-β receptors by dominant-negative plasmids induces hepatocyte proliferation (27), suggesting an antiproliferative effect of TGF-β on liver cells.

Besides proliferation, TGF-β regulates multiple physiological and pathophysiological processes. During liver development, TGF-β signaling is required for hepatoblast differentiation toward intrahepatic cholangiocytes (28). In a mouse model mimicking Alagille syndrome, TGF-β drives formation of novel biliary structure from hepatocytes (29). The critical role of TGF-β in liver fibrosis has been well recognized for decades (30–32). However, precise mechanisms by which TGF-β/SMAD signaling regulates LPC proliferation and differentiation in ALF following MHN remain largely unknown.

Here, we combined spatial transcriptomics and single-cell sequencing of samples from patients and zebrafish with MHN-induced ALF. Cell-cell interaction analyses revealed predominant signaling pathways in LPCs (e.g., TGF-β, HGF, and EGF) produced by hepatic stellate cells (HSCs), macrophages, or both. In normal LPCs, TGF-β inhibited proliferation by blocking G1-to-S phase transition, whereas in ALF, HGF and EGF overrode this inhibition, allowing LPC proliferation. Despite its antiproliferative effect being neutralized, TGF-β/SMAD signaling remained essential for LPCs to transcribe hepatocyte-specific genes, thereby enabling liver functions.

## Results

### Spatial transcriptomics reveals transcriptomes of LPCs, macrophages, and HSCs in patients with ALF.

We first conducted histological examination, including hematoxylin and eosin (H&E), immunohistochemistry (IHC), and immunofluorescence (IF) staining, in 8 patients with irreversible ALF who underwent liver transplantation (Supplemental Figure 1A; supplemental material available online with this article; https://doi.org/10.1172/JCI199913DS1). DRs were characterized by proliferative LPCs surrounded by marked inflammatory cell infiltration (Supplemental Figure 1B). IHC for CD68 and α–smooth muscle actin (α-SMA) demonstrated that LPCs were closely associated with numerous macrophages and activated HSCs (Supplemental Figure 1C). A combination of morphological features and immunohistochemistry for CK19, CK7, and CK8/18 was used to identify LPCs, intermediate hepatocyte-like cells (IHLCs), and mature hepatocytes (Supplemental Figure 1, D and E). Macrophages and activated HSCs were predominantly observed in regions enriched for LPCs. In contrast, inflammatory infiltration and activated HSCs were reduced in areas containing IHLCs, and only rare inflammatory cells and few activated HSCs were detectable in regions composed mainly of mature hepatocytes (Supplemental Figure 1, D and E). These findings suggested that macrophages and activated HSCs may play critical roles in LPC proliferation and differentiation.

**Figure 1:**
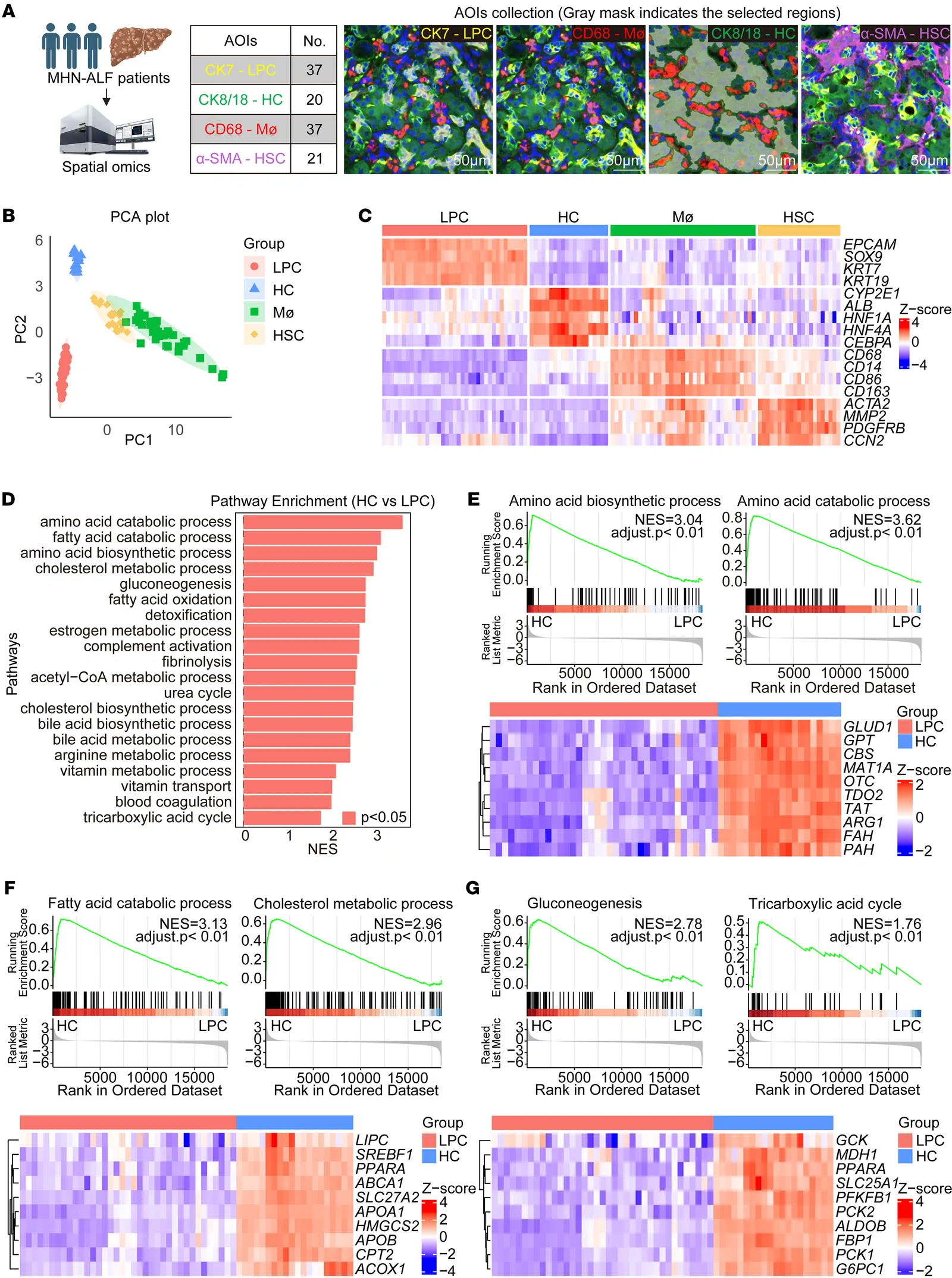
Spatial transcriptomics of LPCs, hepatocytes, macrophages, and HSCs in explanted livers of 4 patients with ALF. (**A**) A scheme depicts the GeoMx DSP workflow. A total of 115 areas of illumination (AOIs) were collected based on cell type–specific markers: CK7^+^ LPCs (yellow, *n* = 37), CK8/18^+^ hepatocytes (HCs) (green, *n* = 20), CD68^+^ macrophages (MΦ) (red, *n* = 37), and α-SMA^+^ HSCs (violet, *n* = 21). Gray-masked regions indicate the precise AOIs selected for transcriptomic profiling within each cell type. (**B**) Principal component analysis (PCA) based on whole-transcriptome profiles shows distinct clustering of LPCs, HCs, macrophages, and HSCs. (**C**) Heatmap illustrates representative cell type–specific gene expression patterns. (**D**) GO BP pathway enrichment analysis was performed to identify enrichments of metabolic function genes between HCs and LPCs, including pathways for amino acid, fatty acid, and cholesterol metabolism; detoxification; and gluconeogenesis. (**E**–**G**) GSEA and corresponding heatmaps reveal transcriptional downregulation of hepatic functional pathways in LPCs, including amino acid metabolism, lipid metabolism, gluconeogenesis, and the tricarboxylic acid cycle. Representative metabolic genes (e.g., *GLUD1*, *PPARA*, *FBP1*, *PCK1*, et al.) are listed.

Next, we performed GeoMx digital spatial profiler (DSP) digital spatial transcriptomics analysis on liver tissues collected from 4 patients with irreversible ALF (Figure 1A). Tissue sections were hybridized with the GeoMx Human Whole Transcriptome Atlas (WTA) probes and stained with multiplexed IF markers. Regions of interest (ROIs) were defined across the tissue, and cell type–specific AOIs were segmented within each ROI for UV photocleavage-mediated release of barcoded oligonucleotides, followed by next-generation sequencing–based quantification (Supplemental Figure 2, A and B). We selected multiple AOIs from different cell types, including CK7-positive LPCs (*n* = 37), CK8/18-positive hepatocytes (*n* = 20), CD68-positive macrophages (*n* = 37), and α-SMA–positive HSCs (*n* = 21) (Figure 1A). Principal component analyses (PCAs) based on the chosen AOIs revealed completely distinct transcriptomic profiles between LPCs and HCs (Figure 1B). There were partially overlapping transcriptomic profiles between macrophages and activated HSCs (Figure 1B). LPCs exhibited high levels of *EPCAM*, *SOX9*, *KRT7*, and *KRT19* transcripts, whereas hepatocytes expressed *CYP2E1*, *ALB*, *HNF1A*, *HNF4A*, and *CEBPA* transcripts (Figure 1C). Macrophages showed high levels of *CD68*, *CD14*, *CD86*, and *CD163* transcripts, whereas activated HSCs expressed *ACTA2*, *MMP2*, *PDGFRB*, and *CCN2* transcripts (Figure 1C).

Gene Ontology Biological Process (GO BP) analysis revealed distinct functional pathways between hepatocytes and LPCs, including amino acid and fatty acid catabolic processes; cholesterol, estrogen, acetyl-CoA, arginine, bile acid, and vitamin metabolic processes; amino acid, bile acid and cholesterol biosynthesis processes; detoxification, fibrinolysis, gluconeogenesis; complement activation; the urea cycle; blood coagulation; and vitamin transport and tricarboxylic acid (TCA) cycle (Figure 1D). Gene set enrichment analysis (GSEA) showed lower expression of genes involved in these pathways in LPCs compared with hepatocytes (Figure 1, E–G). As shown in Figure 1, E–G, genes associated with the biosynthesis and catabolism of amino acids (e.g., *GLUD1*, *GPT*, *CBS*, *TAT*), fatty acids (e.g., *PPARA*, *ACOX1*, *CPT2*), and cholesterol (e.g., *SREBF1*, *ABCA1*, *HMGCS2*), as well as gluconeogenesis (e.g., *G6PC1*, *PCK1*, *FBP1*) and TCA cycle (e.g., *MDH1*, *SLC25A1*), were lower in LPCs. Additional lower genes in LPCs were associated with coagulation factors (e.g., *F2*, *F7*, *F9*, *F10*); detoxification factors (e.g., *GSTA1*, *PRDX1*, *GSTO1*); the estrogen metabolic process (e.g., *UGT2B7*, *HSD17B1*, *CYP2C9*); the bile acid metabolic process (e.g., *CYP27A1*, *ABCB11*, *SULT2A1*); the urea cycle (e.g., *CPS1*, *ARG1*, *OTC*); and vitamin transport (e.g., *SLC23A2*, *SLC22A1*, *TTPA*) (Supplemental Figure 3A).

These pathways are hallmarks of mature liver function and highlight the transcriptional immaturity of LPCs. These results demonstrate the transcriptome changes that occur during the transition from LPCs to mature hepatocytes.

### Cellular communications between LPCs and surrounding cells including macrophages, HSCs, and hepatocytes in ALF.

**Figure 2:**
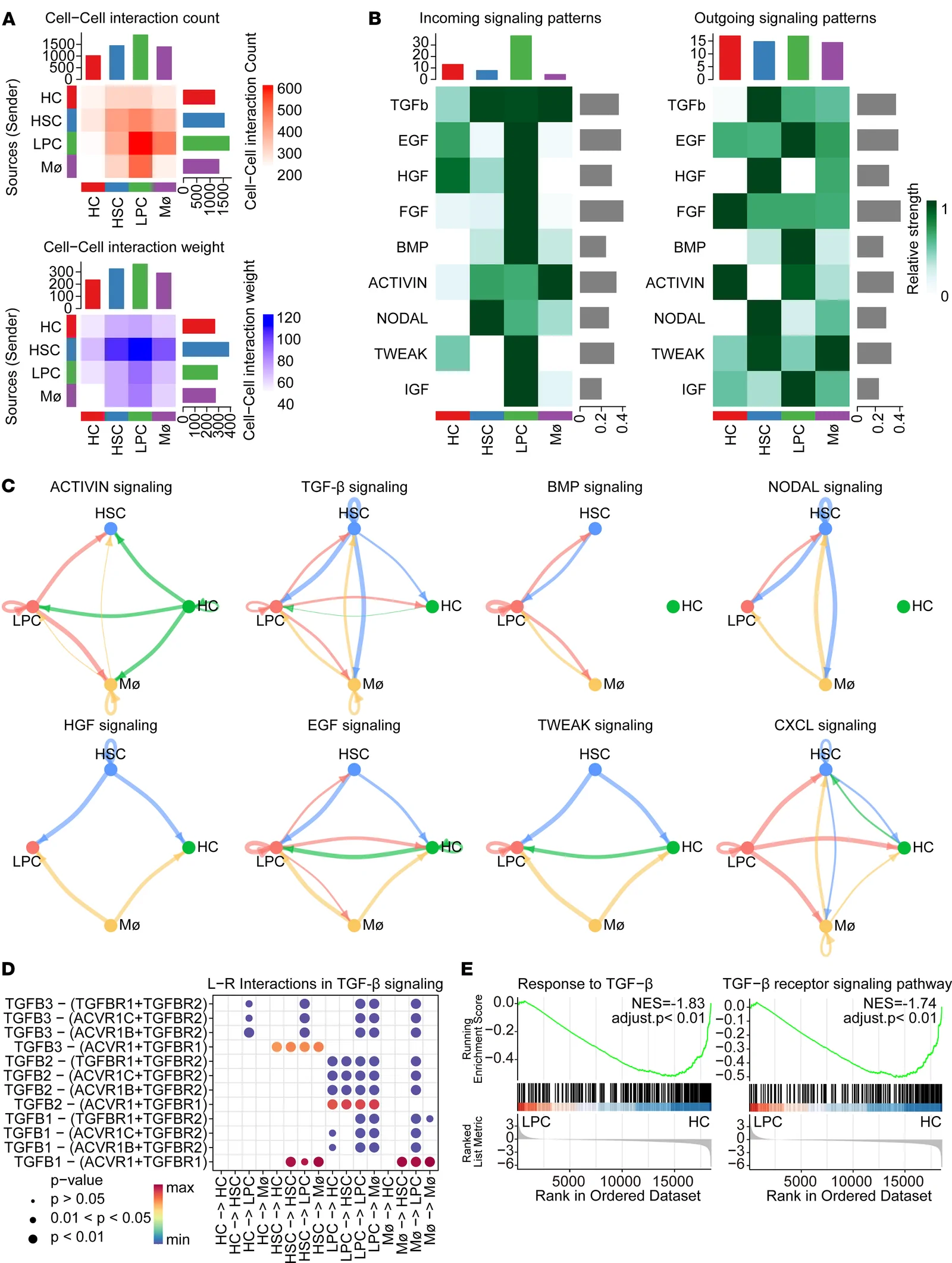
Cell-cell communications between LPCs, macrophages, and HSCs in ALF. (**A**) Cell-cell interaction analysis based on spatial transcriptomic data was performed to evaluate interaction counts and interaction weights between LPCs, macrophages (MΦ), and HSCs. (**B**) Heatmaps summarize the incoming (left) and outgoing (right) signaling patterns in LPCs, macrophages, and HSCs. (**C**) Network diagrams illustrate the directionality and intensity of specific signaling pathways among LPCs, HSCs, macrophages, and HCs. Line thickness indicates interaction strength, and arrow direction denotes the source-to-target relationship. (**D**) Dot plots display specific ligand-receptor (L-R) interactions in TGF-β signaling pathways between different cell populations. (**E**) GSEA highlights enrichment of response to TGF-β and TGF-β receptor signaling pathway gene sets in LPCs compared with HCs.

Subsequently, we analyzed the cellular communications between LPCs, residual hepatocytes, macrophages, and activated HSCs in these patients with irreversible ALF. As shown in Figure 2A, there were high levels of cell-cell interaction counts and weights between LPCs, macrophages, and HSCs. On the one hand, LPCs received multiple strong signals from surrounding cells, including TGF-β, activin, BMP, EGF, HGF, FGF, TWEAK, and IGF (Figure 2B). On the other hand, LPCs also transmitted strong signals to surrounding cells, including TGF-β, activin, BMP, EGF, FGF, and IGF (Figure 2B). Further cell-cell communication analysis revealed the directionality of signal transduction between LPCs and surrounding cells. Macrophages and activated HSCs sent TGF-β, BMP, NODAL, EGF, HGF, and CXCL to LPCs (Figure 2C). LPCs sent TGF-β, activin, BMP, and CXCL to macrophages and TGF-β, activin, BMP, NODAL, EGF, and CXCL to activated HSCs (Figure 2C). In addition, LPCs produced TGF-β, activin, BMP, EGF, TWEAK, and CXCL for autoregulation (Figure 2C). Further ligand-receptor analyses showed that the macrophage- and HSC-derived TGF-β1/ACVR1/TGFBR1 signaling pathway was a predominant signal received by LPCs (Figure 2, D and E), suggesting a potential critical role of this signaling pathway in regulating LPC behavior in ALF. In addition to TGF-β1, high levels of TGF-β2 were produced by LPCs and sent to hepatocytes, macrophages, and HSCs, and back to the LPCs.

Given the complex microenvironment in ALF tissue, partial macrophage contamination within α-SMA–defined AOIs cannot be fully excluded in the GeoMx digital spatial transcriptomics analysis (Supplemental Figure 3, B and C). To confirm the cellular sources of TGF-β1 and HGF in MHN-associated ALF, we performed 10x Genomics Chromium Flex single-cell RNA sequencing (scRNA-seq) on liver tissues from a patient with ALF. Unsupervised clustering identified 13 distinct cell populations, including cholangiocytes, hepatocytes, HSCs, macrophages, and various immune and stromal cell types, which were identified by their marker gene expression (Supplemental Figure 4, A–C). We subsequently focused on 4 major hepatic cell populations: cholangiocytes, hepatocytes, HSCs, and macrophages (Supplemental Figure 4D). The cholangiocyte cluster was defined by high expression of ductal markers *KRT7*, *EPCAM*, *CFTR*, and *SPP1*, while hepatocytes were characterized by robust expression of metabolic and secretory genes, including *APOA1*, *APOA2*, *HP*, and *TTR* (Supplemental Figure 4E). Notably, a set of cholangiocytes exhibited detectable expression of multiple hepatocyte functional genes, including *FGA*, *FGG*, *APOB*, *TF*, *RBP4*, *CYP2E1*, *PAH*, and *AGT* (Supplemental Figure 4E), suggesting partial acquisition of hepatocyte metabolic identity. Consistent with this functional change, transcription factor profiling revealed that a subset of cholangiocytes expressed key hepatocyte transcription factors, including *HNF4A* and *HNF1A* (Supplemental Figure 4F), suggesting that these cells are LPCs expressing hepatocyte functional genes after MHN. Further analysis demonstrated that high levels of *TGFB1* and *HGF* were expressed in macrophages and HSCs, respectively, whereas the HGF receptor *MET* was enriched in cholangiocytes/LPCs (Supplemental Figure 4G).

To further resolve the cellular sources of TGF-β1 and HGF at the macrophage subset level, we performed unsupervised subclustering of the macrophage compartment from the 10x Genomics Chromium Flex dataset. Three subsets were identified: resident Kupffer cells (C1QA, CD5L, VSIG4), recruited monocyte-derived macrophages (S100A8/9, VCAN), and an inflammatory subset (NFKBIA, NLRP3, CXCL2) (Supplemental Figure 5, A and B).

At the whole-liver level, TGFB1 was broadly expressed across multiple cell types, including macrophages, neutrophils, lymphatic endothelial cells, T/NK cells, pDCs, and B cells, whereas HGF was primarily restricted to HSCs (Supplemental Figure 5C). Within the macrophage compartment, TGFB1 was uniformly expressed across all 3 subsets, and HGF expression was sparse and uniformly low (Supplemental Figure 5D). These results indicate that no individual macrophage subpopulation preferentially produces TGF-β1 or HGF; rather, macrophage-derived TGF-β1 represents a broadly shared feature of the compartment.

### Cholangiocytes exhibit robust TGF-β and HGF signaling during their differentiation into hepatocytes in zebrafish undergoing hepatocyte ablation.

To further validate key signaling pathways governing LPC proliferation and differentiation following MHN, we analyzed a publicly available single-cell RNA-seq dataset (NCBI GEO GSE272484), generated from *Tg(fabp10a:CellCousin)* zebrafish subjected to metronidazole-induced (Mtz-induced) hepatocyte ablation (33). It is established that although administration of Mtz in nitroreductase-expressing zebrafish ablates nearly all hepatocytes, the zebrafish survive by inducing cholangiocyte-derived liver regeneration (10, 11, 34).

**Figure 3:**
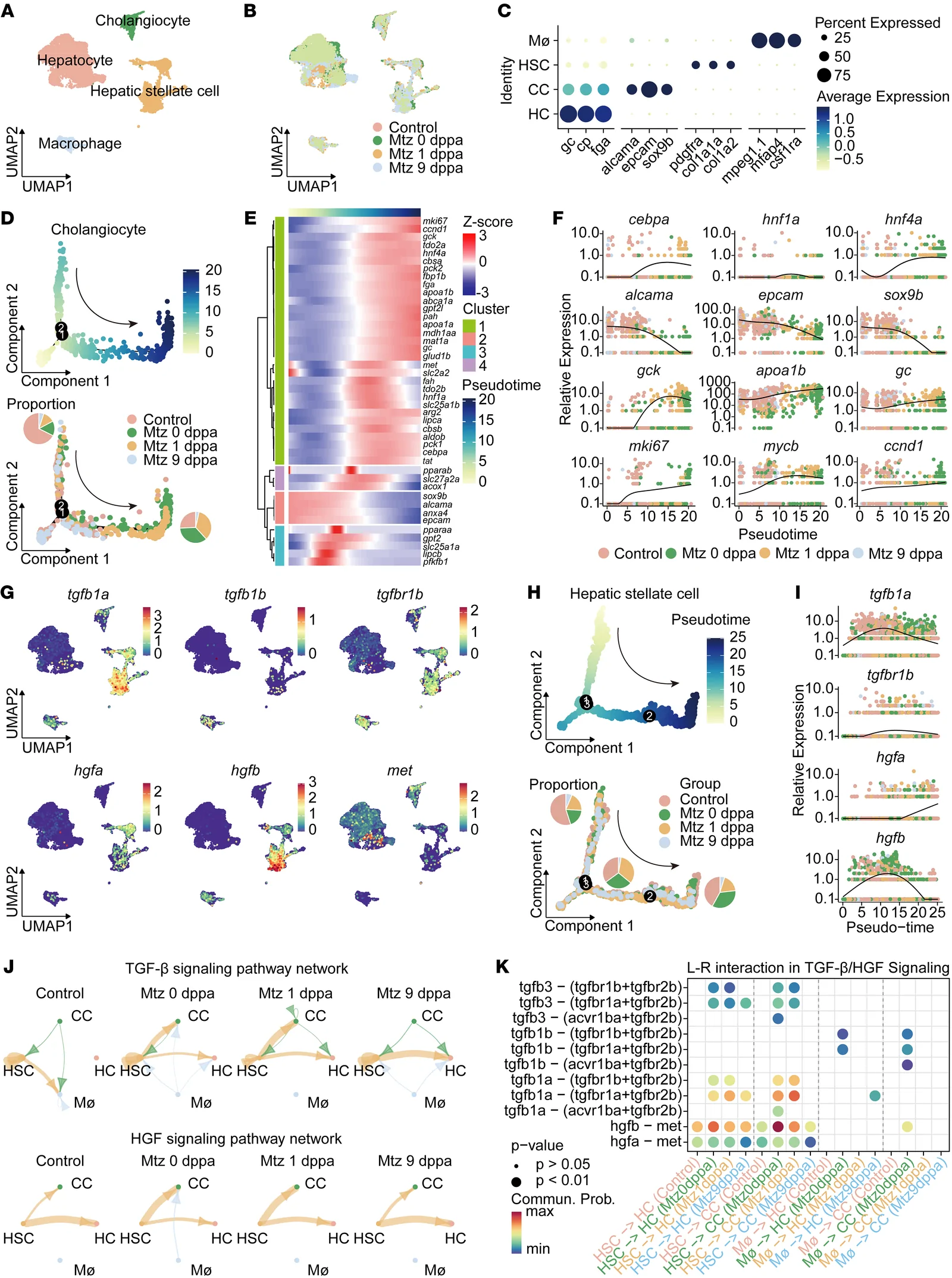
ScRNA-seq reveals TGF-β and HGF signaling during cholangiocyte-to-hepatocyte differentiation following hepatocyte ablation in zebrafish. (**A** and **B**) Uniform manifold approximation and projection (UMAP) clustering and cell cluster annotation — hepatocytes (HC), hepatic stellate cells (HSC), cholangiocytes (CC), and macrophages (MΦ) — based on scRNA-seq data from the livers of Mtz-treated *Tg(fabp10a:CellCousin)* zebrafish at control and 0, 1, and 9 dppa. (**C**) A dot plot illustrates the expression of representative genes in HC, HSC, CC, and MΦ. (**D**) Pseudotime trajectory analysis of CC reveals a branched differentiation path (top, colored by pseudotime; bottom, by group), with branch points labeled 1 and 2. Pie charts indicate the relative proportions of cells at corresponding pseudotime stages. (**E**) A heatmap shows dynamic expression of selected genes along the pseudotime trajectory. Genes are clustered into 4 clusters by expression patterns. (**F**) Pseudotime plots show the expression of selected genes along the trajectory. The black line indicates the overall trend in gene expression across pseudotime. (**G**) UMAP plots show the expression of *tgfb1a*, *tgfb1b*, *tgfbr1b*, *hgfa*, *hgfb*, and *met* in different cell clusters. (**H**) Pseudotime trajectory analysis of HSCs reveals a branched differentiation path (top, colored by pseudotime; bottom, by group), with branch points labeled 1, 2, and 3. (**I**) Pseudotime plots show the dynamic expression of hgfa, hgfb, tgfb1a, and tgfbr1b along pseudotime. (**J**) Network diagrams illustrate the directionality and intensity of TGF-β and HGF signaling among CC, HSC, MΦ, and HC. Line thickness indicates interaction strength, and arrow direction denotes the source-to-target relationship. (**K**) Dot plots display specific ligand-receptor (L-R) interactions in TGF-β and HGF signaling pathways between different cell populations.

According to scRNA-seq, hepatocytes, cholangiocytes, HSCs, and macrophages in the livers of zebrafish at 0, 1, and 9 days post partial ablation (dppa) were clustered using Seurat (Figure 3, A and B). Hepatocytes, cholangiocytes, HSCs, and macrophages were identified by the expression of canonical markers: *gc*, *cp*, and *fga* for hepatocytes; *alcama*, *epcam*, and *sox9b* for cholangiocytes; *pdgfra*, *col1a1a*, and *col1a2* for HSCs; and *mpeg1.1*, *mfap4*, and *csf1ra* for macrophages (Figure 3C).

RNA-based cellular trajectory analysis revealed a cholangiocyte lineage trajectory, in which a subset of cholangiocytes progressively differentiated to hepatocytes (Figure 3D). Pseudotime analysis further showed that genes active at early pseudotime, 0–5, were enriched in uninjured zebrafish livers, including *met*, *slc2a2*, *sox9b*, *alcama*, *anxa4*, and *epcam* (Figure 3E). At later pseudotime stages, 10–20, gene activity shifted toward those associated with proliferation (e.g., *mki67*, *ccnd1*), hepatocyte master transcription factors (e.g., *hnf4a*, *hnf1a*, *cebpa*), and liver function (e.g., *gck*, *gc*, *pck1*, *apoa*). Dynamic pseudotime plots further revealed rapidly upregulated genes, including hepatocyte transcription factors (*hnf4a*, *cebpa*), liver function genes (*gc*, *apoa1b*), and proliferative markers (*mycb*, *mki67*, *ccnd1*), along with downregulated cholangiocyte identity genes (*sox9b*, *alcama*, *epcam*), in zebrafish from 0 to 9 dppa (Figure 3F). These results are consistent with findings from spatial transcriptomic analysis in human ALF (Figure 1), in which the active gene transcriptome shifted from LPC-related to hepatocyte-related gene expression.

Next, we analyzed the spatial distribution and dynamic expression of genes relevant to TGF-β and HGF signaling. UMAP plots demonstrated that *tgfb1a*, *tgfb1b*, *hgfa*, and *hgfb* were primarily expressed in HSCs, while their corresponding receptors, *tgfbr1b* and *met*, were detected in both hepatocytes and cholangiocytes (Figure 3G). Pseudotime analysis showed dynamic changes in HSC distribution in the livers of zebrafish from 0 to 9 dppa (Figure 3H). The highest HSC activity was observed 0 dppa, immediately following hepatocyte ablation (Figure 3H). At this point, HSC-derived *tgfb1a*, *hgfa*, and *hgfb* were predominant (Figure 3I).

Subsequently, we performed CellChat analysis to characterize TGF-β and HGF ligand-receptor interactions between liver cell types in zebrafish. In healthy zebrafish, HSCs were the primary source of both TGF-β and HGF (Figure 3J). HSC-derived TGF-β signaling was mainly directed toward HSCs themselves and macrophages (Figure 3J). After hepatocyte ablation (0 and 1 dppa), the HSC-derived TGF-β signal shifted from targeting macrophages to cholangiocytes and hepatocytes (Figure 3J). By 9 dppa, the TGF-β signal from HSC was directed exclusively toward hepatocytes (Figure 3J). In healthy zebrafish, HSC-derived HGF signal was directed to both hepatocytes and cholangiocytes (Figure 3J). Notably, hepatocytes received a stronger HGF signal than cholangiocytes under physiological conditions (Figure 3J). Following hepatocyte ablation (0 and 1 dppa), the HGF signal from HSCs was primarily directed to cholangiocytes rather than hepatocytes (Figure 3J). By 9 dppa, this signal was once again predominantly directed toward hepatocytes (Figure 3J). Ligand-receptor analysis further revealed that among TGF-β family members, tgfb1a is the predominant cytokine in the zebrafish liver (Figure 3K). At 0 and 1 dppa, HSC-derived tgfb1a and receptors (tgfbr1a and tgfbr2b) on cholangiocytes and hepatocytes formed strong interactions (Figure 3K). By 9 dppa, this ligand-receptor interaction was observed only between HSCs and hepatocytes (Figure 3K). Consistent with the CellChat analysis, HSC-derived hgfb and its receptor met on both hepatocytes and cholangiocytes formed interactions at different points (Figure 3K). The hgfb-met interaction was stronger between HSCs and hepatocytes under healthy conditions and at 9 dppa, whereas it was more robust between HSCs and cholangiocytes at 0 and 1 dppa (Figure 3K).

These findings align with observations in patients with ALF, where following MHN, LPCs in patients and cholangiocytes in zebrafish receive robust TGF-β and HGF signals from HSCs.

### TGF-β controls LPC proliferation.

The potential importance of the TGF-β signaling pathway has also been demonstrated in a recent spatiotemporal analysis of mice fed with 3,5-diethoxycarbonyl-1,4-dihydrocollidine (DDC) (35). In DDC-fed mice, TGF-β signaling was identified as a predominant pathway regulating DDC-induced DRs and liver tissue recovery after cessation of DDC toxicity (35). Given the key role of TGF-β in cell proliferation (24), we examined liver tissues collected from wild-type and *Smad7*-transgenic mice fed with DDC for 4 weeks. SMAD7 is a negative feedback regulator that terminates TGF-β signaling (36).

**Figure 4:**
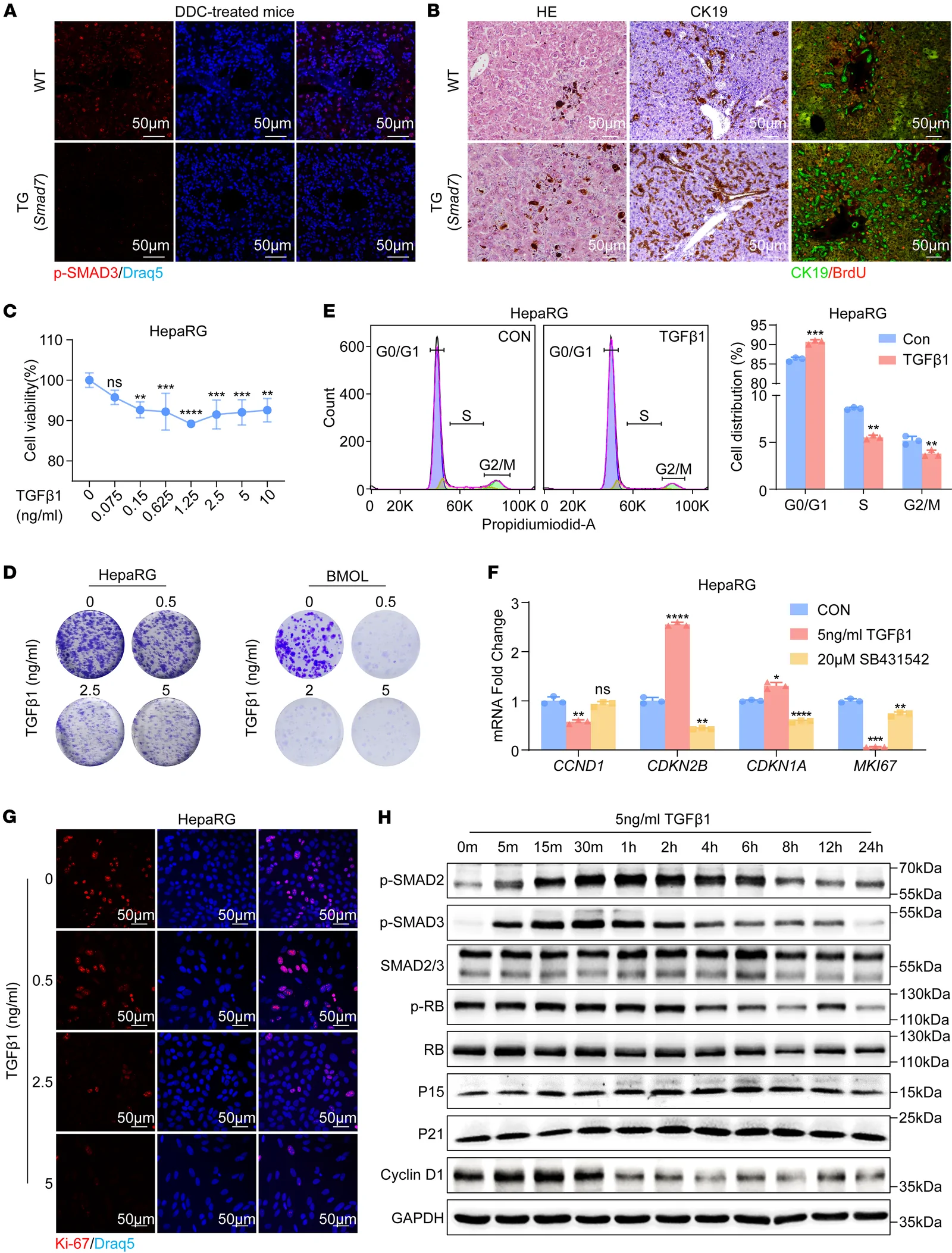
TGF-β controls LPC proliferation. (**A**) IF staining for p-SMAD3 was performed on liver tissues from wild-type (WT) and *Smad7*-transgenic (TG) mice fed with DDC for 4 weeks. (**B**) H&E staining, IHC and IF for CK19 (green) and bromodeoxyuridine (BrdU; red) were performed on liver tissues collected from mice fed with DDC for 4 weeks. Mice received BrdU injection 2 hours before sacrifice. (**C**) Cell viability was analyzed in HepaRG cells treated with different concentrations of TGF-β1 for 48 hours using the CCK-8 assay. (**D**) A colony formation assay was performed on HepaRG and BMOL cells exposed to various concentrations of TGF-β1 for 14 days. (**E**) Flow cytometric analysis was conducted to examine the cell cycle distribution in HepaRG cells treated with or without TGF-β1 for 24 hours. (**F**) qRT-PCR was used to analyze mRNA expression of *CCND1*, *CDKN2B*, *CDKN1A*, and *MKI67* in HepaRG cells treated with TGF-β1 and SB431542. (**G**) IF staining for Ki-67 was performed on HepaRG cells treated with TGF-β1 at indicated concentrations. (**H**) Western blotting was conducted to analyze SMAD and cell cycle–related proteins in HepaRG cells treated with 5 ng/mL TGF-β1 at different time points. Scale bars: 50 μm (**A**, **B**, and **G**). Data are presented as mean ± SD. *P* < 0.05 (*), *P* < 0.01 (**), *P* < 0.001 (***), *P* < 0.0001 (****). 1-way ANOVA/Dunnett (**C**); unpaired 2-tailed Welch’s *t* test (**E** and **F**).

Compared with DDC-fed wild-type mice, phosphorylated SMAD3 (p-SMAD3) expression in *Smad7*-transgenic mice was markedly reduced (*P* < 0.001) (Figure 4A and Supplemental Figure 6A), indicating suppression of TGF-β signaling in these mice. Concomitant with the inhibition of TGF-β signaling, H&E staining revealed markedly expanded DR areas in *Smad7*-transgenic mice compared with wild-type controls (Figure 4B). IHC for CK19 confirmed a significant increase in LPC numbers in *Smad7*-transgenic mice, with integrated optical density/area analysis demonstrating approximately a 2-fold increase compared with wild-type controls (11.624 ± 1.88 vs. 6.228 ± 1.23, *P* < 0.0001) (Figure 4B and Supplemental Figure 6, B and C). IF costaining for CK19 and bromodeoxyuridine (BrdU) confirmed enhanced LPC proliferation in *Smad7*-transgenic mice (*P* < 0.0001) (Figure 4B and Supplemental Figure 6D). These results suggest that TGF-β acts as an antiproliferative regulator in LPC proliferation.

To further investigate how TGF-β1 regulates LPC proliferation, we performed a cell counting kit-8 (CCK-8) assay in the human LPC line HepaRG cells and murine LPC line BMOL cells, which were treated with varying concentrations of TGF-β1. As shown in Figure 4C and Supplemental Figure 6E, TGF-β1 reduced LPC cell viability in a dose-dependent manner. To examine whether TGF-β1–induced reduction in cell viability was due to apoptosis, we performed a caspase-3 activity assay in HepaRG cells treated with or without TGF-β1 (5 ng/mL for 24 hours). TGF-β1 treatment did not induce apoptosis in LPCs (Supplemental Figure 6F). These results indicate that the reduction in cell viability caused by TGF-β1 is more likely attributable to cell cycle arrest than to apoptotic cell death.

A colony formation assay also demonstrated that TGF-β1 dose-dependently decreased LPC colony formation (Figure 4D). Flow cytometric (FACS) analysis further revealed that TGF-β1 arrested LPCs in the G1 phase, preventing their entry into the S phase (Figure 4E). Consistent with the FACS finding, qRT-PCR analysis showed that TGF-β1 inhibited the mRNA expression of *CCND1* while increasing the expression of *CDKN2B* and *CDKN1A* (Figure 4F and Supplemental Figure 6G). Furthermore, both mRNA and protein expression of *MKI67* were significantly inhibited in TGF-β1–treated HepaRG cells and BMOL cells (Figure 4, F and G, and Supplemental Figure 6, H and I). The effects were reversed by the TGF-β receptor I inhibitor SB431542 (Figure 4F).

Next, we performed Western blot analysis to examine dynamic alterations of cell cycle–regulatory proteins in HepaRG cells under TGF-β1 stimulation. TGF-β1–induced p-SMAD2 and p-SMAD3 levels rapidly increased within 5 minutes of stimulation and remained elevated for up to 24 hours (Figure 4H and Supplemental Figure 6I). TGF-β1 stimulation decreased cyclin D1 and p-RB expression over time while rapidly increasing p15 and p21 levels, indicating TGF-β1–induced cell cycle arrest (Figure 4H and Supplemental Figure 6J). These results suggest that TGF-β1 plays a critical role in controlling the G1-to-S phase transition in LPCs, similar to its role in other cells (37).

### Active TGF-β signaling in proliferating LPCs.

**Figure 5:**
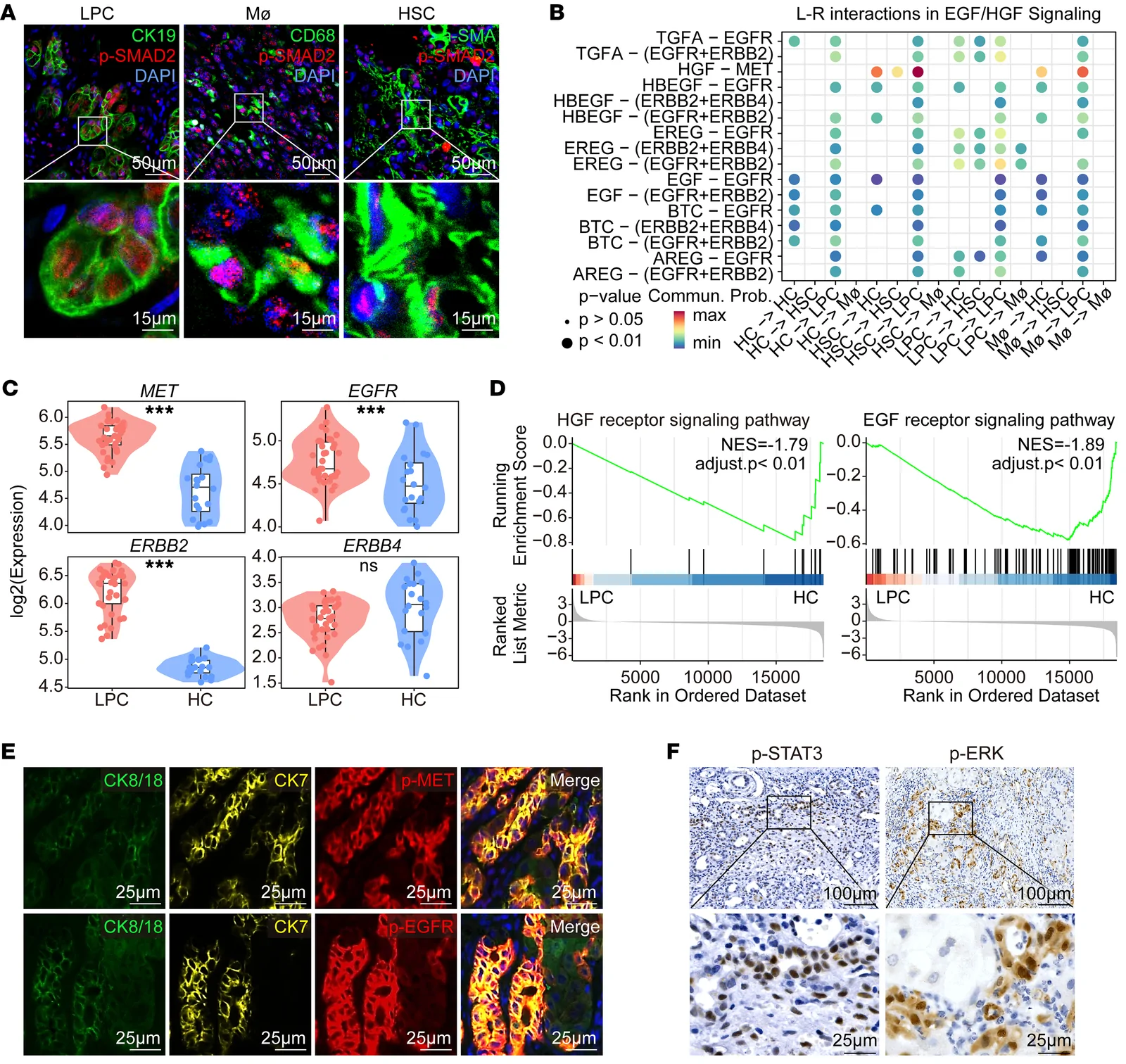
TGF-β, HGF, and EGF signaling are predominant in proliferating LPCs. (**A**) IF costaining for p-SMAD2 (red), a cell type marker (green; CK19 for LPCs, CD68 for macrophages, or α-SMA for HSCs), and DAPI (blue) in liver tissues from patients with ALF. Boxed areas are magnified below. Scale bars: 50 μm (top), 15 μm (bottom). (**B**) Ligand-receptor interaction analysis focusing on EGF and HGF signaling pathways was conducted to predict communication intensity between LPCs, HSCs, MΦ, and HCs. (**C**) Violin plots display expression levels of *MET*, *EGFR*, *ERBB2*, and *ERBB4* in LPCs and HCs. (**D**) GSEA was performed to compare HGF and EGF receptor signaling pathways between LPCs and HCs. (**E**) IF staining for CK8/18 (green), CK7 (yellow), and p-EGFR or p-MET (red) was conducted on liver tissues from patients with ALF. Scale bar: 25 μm. (**F**) IHC staining of p-STAT3 and p-ERK was conducted on liver tissues from patients with ALF. Scale bars: 25 and 100 μm. Data are presented as mean ± SD. *P* < 0.001 (***). Mann-Whitney *U* test (**C**).

To assess TGF-β signaling activity in LPCs, we performed IHC to examine the expression of p-SMAD2 in 8 patients with irreversible ALF. As shown in Figure 5A, robust p-SMAD2 expression was observed in LPCs, macrophages, and HSCs, indicating active TGF-β signaling in these cells. Interestingly, these cells, including LPCs, simultaneously expressed high levels of c-MYC (Supplemental Figure 7A), suggesting that TGF-β signaling does not inhibit LPC proliferation in ALF following MHN.

How can LPCs proliferate in the presence of active TGF-β/SMAD signaling? Spatial transcriptomic and ligand-receptor analyses showed that, in addition to TGF-β, LPCs received multiple proliferative signals from surrounding cells, including TGF-α, EGF, HGF, HBEGF, EREG, and AREG (Figure 5B). Among these signals, macrophage- and HSC-derived HGF/MET signaling provided the critical proliferative stimulus for LPCs (Figure 5B). GSEA revealed a significant increase in the expression of HGF and EGF receptor genes (e.g., *MET*, *EGFR*, *ERBB2*, and *ERBB4*) in LPCs compared with hepatocytes (Figure 5, C and D). The substantial expression of p-MET and p-EGFR in LPCs — but not in hepatocytes — was confirmed in the same 4 patients with ALF through co-immunofluorescence staining (Figure 5E). Subsequently, we performed IHC for p-STAT3 and p-ERK in these patients. As shown in Figure 5F and Supplemental Figure 7B, both p-STAT3 and p-ERK were positively expressed in LPCs. These findings indicate that HGF and EGF signaling pathways are activated in LPCs.

### HGF promotes proliferation of liver progenitor cells under TGF-β stimulation.

Compared with healthy individuals, serum concentrations of HGF in patients with fulminant hepatic failure were markedly elevated (from 0.27 ± 0.08 ng/mL to 16.40 ± 14.67 ng/mL) (38). Therefore, we examined the effects of HGF on LPC proliferation.

**Figure 6:**
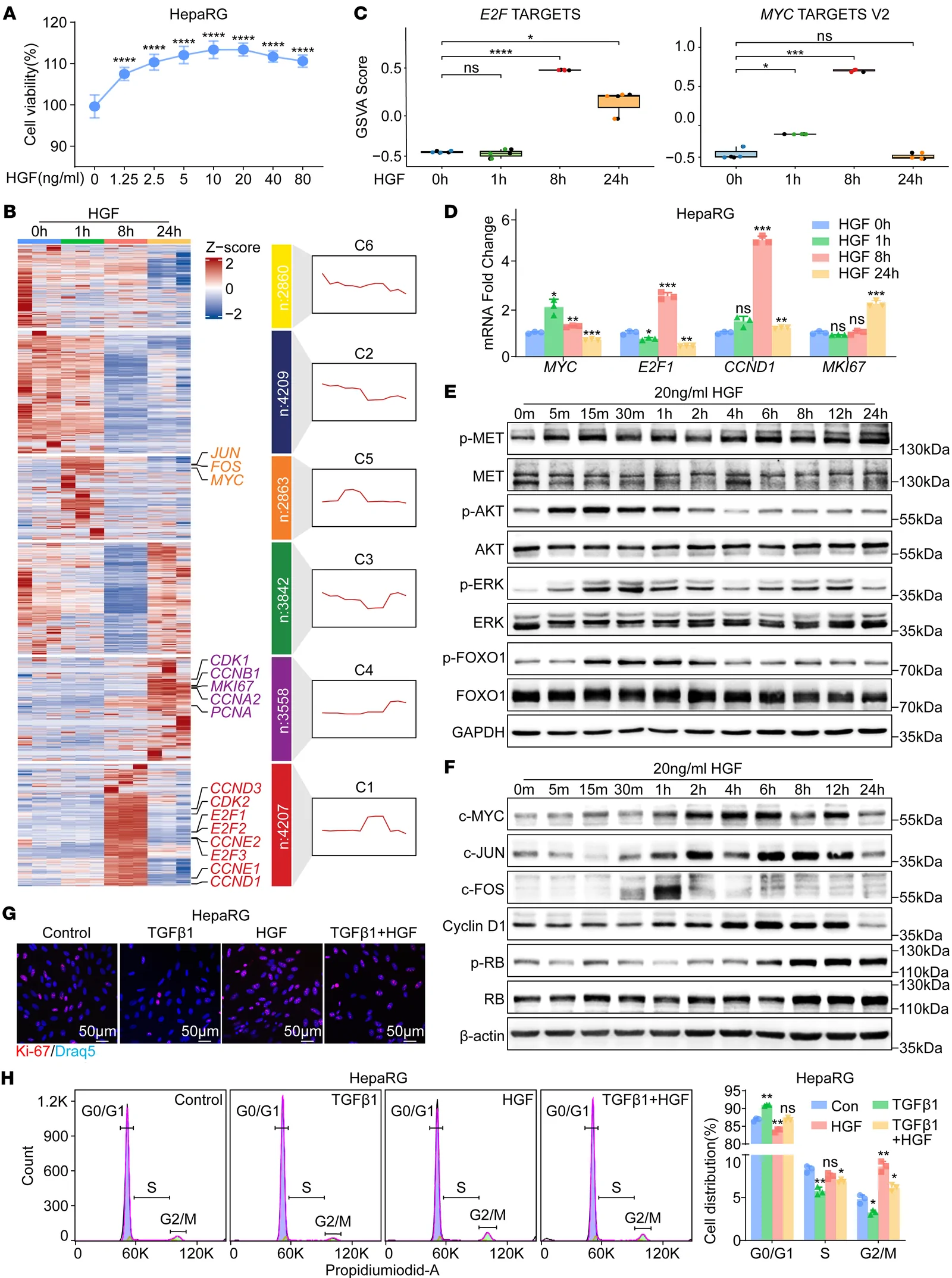
HGF overrides TGF-β–mediated cell cycle arrest in LPCs. (**A**) Cell viability was assessed in HepaRG cells treated with different concentrations of HGF for 48 hours using the CCK-8 assay. (**B**) A heatmap displays transcriptomic changes in HepaRG cells treated with 20 ng/mL HGF for 0, 1, 8, and 24 hours. Genes were grouped into 6 clusters (C1–C6) based on distinct expression patterns. Altered cell cycle– and transcription factor–related genes are annotated within each cluster. Corresponding enrichment curves and gene counts illustrate dynamic expression trends over time. (**C**) GSVA scores were calculated to assess E2F and MYC target gene sets at indicated time points. (**D**) qRT-PCR was performed to examine mRNA expression of *MYC*, *E2F1*, *CCND1*, and *MKI67* in HepaRG cells treated with HGF for the indicated durations. (**E**) Western blot analysis was conducted to assess phosphorylated and total MET, AKT, ERK, and FOXO1 in HepaRG cells treated with 20 ng/mL HGF for the indicated time points. (**F**) Western blotting was used to analyze protein expression of c-MYC, c-JUN, c-FOS, cyclin D1, and phosphorylated and total RB in HepaRG cells stimulated with 20 ng/mL HGF for different periods. (**G**) IF staining for Ki-67 and Draq5 was performed in HepaRG cells treated with TGF-β1, HGF, or a combination of both. Scale bar: 50 μm. (**H**) Flow cytometry was used to analyze cell cycle distribution in HepaRG cells under TGF-β1, HGF, and TGF-β1+HGF treatment. The right panel summarizes the percentage of cells in the G0/G1, S, and G2/M phase. Data are presented as mean ± SD. *P* < 0.05 (*), *P* < 0.01 (**), *P* < 0.001 (***), *P* < 0.0001 (****). 1-way ANOVA with Dunnett’s test (**A** and **C**); 2-tailed Welch’s *t* test vs. Con (**D** and **H**).

CCK-8 assay showed that HGF dose-dependently increased the cell viability of HepaRG and BMOL cells (Figure 6A and Supplemental Figure 8A). Colony formation assay further confirmed that HGF dose-dependently promoted BMOL cell proliferation (Supplemental Figure 8B). To investigate how HGF drives LPC proliferation, we stimulated LPCs with HGF for different durations (e.g., 1 hour, 8 hours, and 24 hours) and performed a time-resolved transcriptomic analysis (Figure 6B). Unsupervised clustering revealed 6 distinct gene expression modules (C1, C2, C3, C4, C5, and C6) after dynamic HGF stimulation (Figure 6B): Module C1 comprises 4,207 genes, which reached peak expression at 8 hours after HGF stimulation; Module C2 contains 4,209 genes, which were downregulated following HGF stimulation for 8 hours and 24 hours; Module C3 consists of 3,842 genes, which were inhibited by HGF stimulation for 1 hour and 8 hours and then restored to baseline levels at 24 hours poststimulation; Module C4 comprises 3,558 genes, which remained stable after 1 hour and 8 hours of HGF stimulation but were upregulated after 24 hours of treatment; Module C5 includes 2,863 genes, which were upregulated by HGF stimulation for 1 hour and subsequently downregulated to normal levels; and Module C6 has 2,860 genes, which exhibited stable expression after 1 hour and 8 hours of HGF stimulation but were downregulated at 24 hours. Gene set variation analysis (GSVA) further showed that transient activation of the *MYC* target gene signature was induced by HGF stimulation for 1 hour and peaked at 8 hours. In contrast, the *E2F* target gene signature reached its peak 8 hours after HGF stimulation and remained elevated until 24 hours (Figure 6C).

Subsequent qPCR assay revealed that HGF stimulation for 1 hour significantly increased mRNA expression of *MYC* (Figure 6D). *E2F1* and *CCND1* expression were upregulated after 8 hours of HGF stimulation (Figure 6D). HGF incubation for 24 hours resulted in high levels of *MKI67* mRNA (Figure 6D). In BMOL cells, HGF similarly upregulated *Ccnd1* and *Mki67* expression in a time-dependent manner, while concomitantly reducing the expression of the CDK inhibitors *Cdkn2b* and *Cdkn1a* (Supplemental Figure 8C). Subsequently, we performed Western blotting to examine the dynamic expression of HGF downstream signaling molecules and targeted proteins in HepaRG cells. HGF administration for 5 minutes was sufficient to induce high levels of expression of p-MET, p-AKT, and p-ERK in LPCs (Figure 6E and Supplemental Figure 8D). p-FOXO1 expression was induced after 15 minutes of HGF incubation (Figure 6E and Supplemental Figure 8D). The peak levels of p-MET persisted until 24 hours, whereas p-AKT and p-FOXO1 expression was maintained for only 2 hours (Figure 6E and Supplemental Figure 8D). p-ERK expression lasted up to 12 hours (Figure 6E and Supplemental Figure 8D). Administration of HGF significantly induced the expression of c-MYC, c-JUN, cyclin D1, and p-RB in LPCs (Figure 6F and Supplemental Figure 8D). Expression of c-MYC, c-JUN, and cyclin D1 was robustly upregulated after 1 hour of HGF incubation and lasted for 12 hours (Figure 6F and Supplemental Figure 8D). p-RB expression was upregulated after 6 hours of HGF incubation and persisted until 24 hours (Figure 6F and Supplemental Figure 8D).

Colony formation assay further showed that HGF significantly promoted BMOL cell proliferation even in the presence of TGF-β1 (Supplemental Figure 9A). IF staining further revealed that HGF increased Ki-67 expression in HepaRG and BMOL cells even in the presence of TGF-β1 stimulation (Figure 6G and Supplemental Figure 9, B and C). FACS analysis further demonstrated that HGF promoted G1-to-S phase progression in HepaRG and BMOL cells, regardless of TGF-β stimulation (Figure 6H and Supplemental Figure 9D). HGF reversed the TGF-β1–induced upregulation of cell cycle inhibitors CDKN1A and CDKN2B at the mRNA level in HepaRG cells (Supplemental Figure 9E). These results suggest that HGF largely overrides the ability of TGF-β1 to arrest LPCs from entering the S phase from the G1 phase.

### EGF promotes LPC proliferation in the presence of TGF-β.

In addition to HGF, we investigated how EGF regulates LPC proliferation. CCK-8 and colony formation assays showed that EGF increased cell viability and colony formation in a dose-dependent manner in HepaRG and BMOL cells (Supplemental Figure 10, A and B). qRT-PCR analysis further revealed that EGF stimulation significantly upregulated *MYC* and *CCND1* mRNA expression in both HepaRG and BMOL cells (Supplemental Figure 10C). Dynamic Western blot analysis showed that EGF administration for 5 minutes rapidly induced the expression of p-ERK, p-AKT, and p-FOXO1 (Supplemental Figure 10, D and E). The peak levels of these proteins persisted for up to 1 hour (Supplemental Figure 10, D and E). EGF downstream transcription factor c-FOS was induced 30 minutes after EGF stimulation and remained elevated for up to 2 hours (Supplemental Figure 10, D and E). Additionally, EGF stimulation for 5 minutes was sufficient to induce high levels of the expression of c-MYC, p-RB, and cyclin D1, with these effects lasting for more than 12 hours (Supplemental Figure 10, D and E). ChIP-qPCR analysis further demonstrated that c-MYC binds to the *CCND1* promoter (–247 bp ~ –31 bp and –797 bp ~ –593 bp) upon EGF stimulation in HepaRG cells (Supplemental Figure 10F).

FACS assay further demonstrated that EGF promoted G1-to-S phase progression in HepaRG and BMOL cells (Supplemental Figure 11A). In the presence of EGF, the ability of TGF-β1 to arrest LPCs from entering the S phase was largely compromised (Supplemental Figure 11A). Consistent with the FACS analysis, a colony formation assay showed that EGF incubation significantly promoted cell proliferation, even in the presence of TGF-β1 (Supplemental Figure 11B). Furthermore, IF staining revealed that EGF increased Ki-67 expression in HepaRG and BMOL cells treated with TGF-β1 (Supplemental Figure 11C). EGF reversed the TGF-β1–induced upregulation of cell cycle inhibitors *CDKN1A* and *CDKN2B* at the mRNA level in HepaRG cells (Supplemental Figure 11D). These results suggest that EGF counteracts the antiproliferative effect of TGF-β on LPCs.

To further clarify the effects of HGF-MET and EGF-EGFR on LPC proliferation and hepatocyte function gene expression, we performed siRNA-mediated knockdown of *MET* and *EGFR* in HepaRG cells. As shown in Supplemental Figure 12, A and B, knockdown of *MET* by RNAi significantly reduced both the proliferation marker *MKI67* expression and hepatocyte functional genes, including *CPS1* and *APOB*, demonstrating that MET signaling is essential for both LPC proliferation and hepatocyte function gene expression. In contrast, knockdown of *EGFR* by RNAi resulted in upregulation of *MKI67* and hepatocyte markers (*HNF4A*, *CPS1*, *APOB*, *F10*), suggesting that similar to HGF, EGFR signaling is also required for LPC proliferation; however, it suppresses hepatocyte function gene expression.

Consistent with the findings of a previous study (39), these results indicate that MET and EGFR collaborate to increase the proliferation of LPCs. MET is a key player in inducing hepatocyte function gene expression. In contrast, EGFR suppresses hepatocyte commitment.

### TGF-β signaling is essential for cholangiocytes/LPCs to activate HGF-dependent liver function genes.

Based on the results obtained above, TGF-β provides predominant signaling in activated human LPCs and zebrafish cholangiocytes following MHN. Since its antiproliferative effect on LPCs can be counteracted by growth factors such as HGF, the role of TGF-β signaling in LPCs and cholangiocytes under these conditions remained unclear.

**Figure 7:**
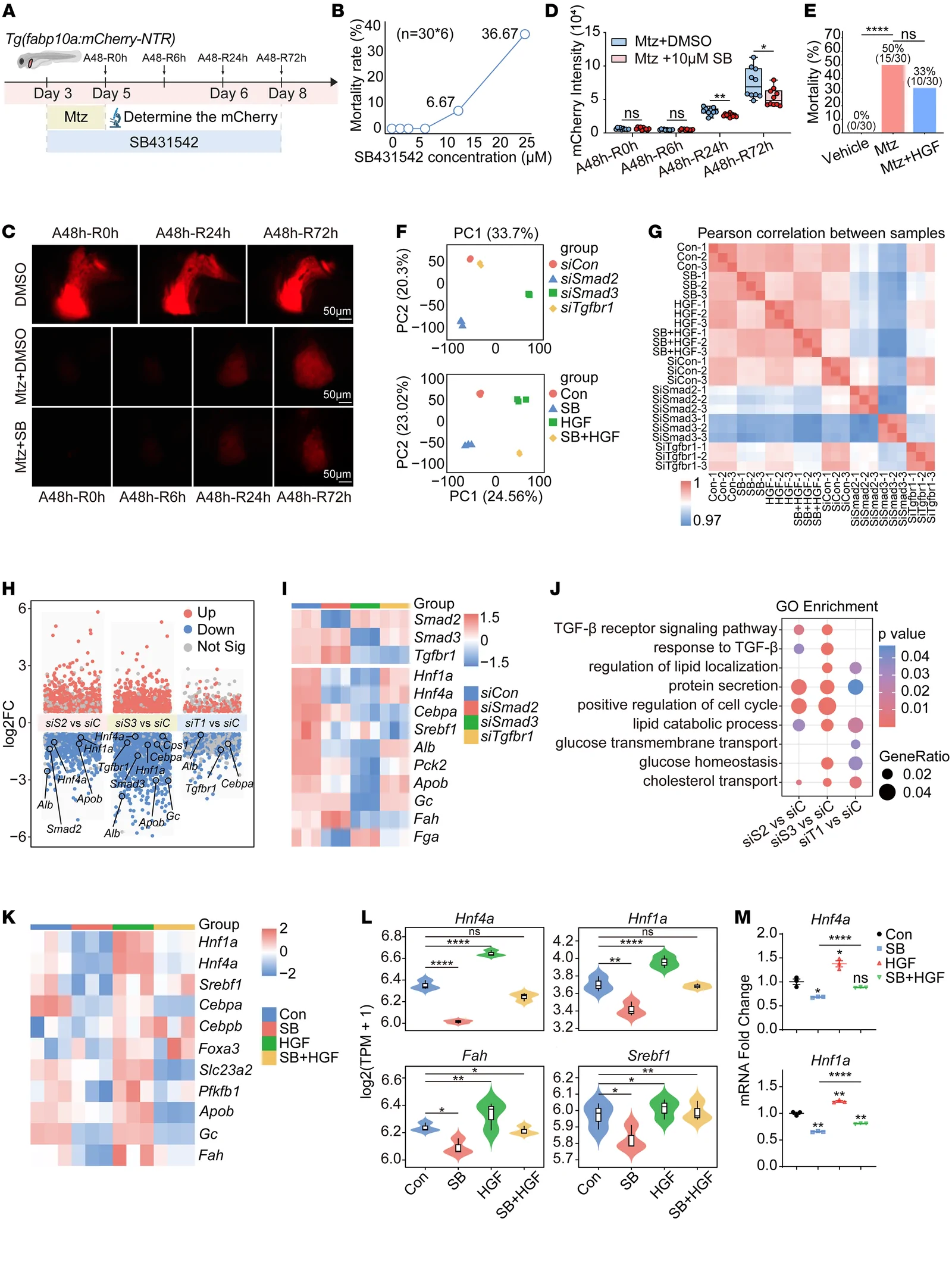
TGF-β signaling is required for cholangiocyte/LPC-mediated liver regeneration and hepatocyte gene activation. (**A**) A diagram depicts the experimental timeline in *Tg(fabp10a:mCherry-NTR)* zebrafish. Zebrafish were treated with Mtz from 3 to 5 days postfertilization (dpf) to induce hepatocyte ablation. Zebrafish were treated with SB431542 from 3 to 8 dpf. (**B**) Zebrafish (*n* = 30 × 6) were treated with different concentrations of SB431542 from 3 to 8 dpf to assess mortality. (**C** and **D**) Representative fluorescence images show mCherry expression at indicated time points in zebrafish treated with Mtz + DMSO, or Mtz +10 μM SB431542. Scale bar: 50 μm. (**E**) Mortality rates of *Tg(fabp10a:mCherry-NTR)* zebrafish treated with vehicle, Mtz (15.0 mM), or Mtz (15.0 mM) + HGF (10 nL × 50 ng/μL, injected at 3, 5, and 7 dpf) (*n* = 30 per group). (**F**) PCA shows distinct clustering of BMOL cells transfected with *siCon*, *siSmad2*, *siSmad3*, or *siTgfbr1* and cells treated with SB431542, HGF, and SB431542 + HGF. (**G**) Pearson’s correlation heatmap presents RNA-seq samples’ relationships in BMOL cells. (**H**) Volcano plot presents differentially expressed genes in BMOL cells transfected with *siSmad2* (*siS2*), *siSmad3* (*siS3*), or *siTgfbr1* (*siT1*), compared with *siCon* (*siC*). (**I**) A heatmap shows expression of representative liver-related genes in the *siCon*, *siSmad2*, *siSmad3*, and *siTgfbr1* groups. (**J**) GO BP enrichment analysis of downregulated genes in BMOL cells transfected with *siS2*, *siS3*, or *siT1*. (**K**) A heatmap shows expression of representative liver-related genes in the Con, SB431542, HGF, and SB431542 + HGF group. (**L**) Violin plots show expression of *Hnf4a*, *Hnf1a*, *Fah*, and *Srebf1*. (**M**) qRT-PCR shows the mRNA expression of *Hnf4a* and *Hnf1a*. Data are shown as mean ± SD. *P* < 0.05 (*), *P* < 0.01 (**), *P* < 0.001 (***), *P* < 0.0001 (****). Two-tailed Welch’s *t* test (**D**, **L**, and **M**); 2-sided Fisher’s exact test vs. Mtz (**E**).

To address this question, we used the general TGF-β receptor inhibitor SB431542 to treat *Tg(fabp10a:mCherry-NTR)* zebrafish exposed to Mtz (Figure 7A). Exposure to Mtz for 2 days resulted in complete hepatocyte ablation in *Tg(fabp10a:mCherry-NTR)* zebrafish; however, all fish survived and gradually restored parenchymal mass by activating cholangiocyte differentiation into hepatocytes (Figure 7C). Low doses of SB431542 (1.56, 3.12, or 6.25 μM) did not affect zebrafish survival. However, 12.5 μM SB431542 resulted in 6.67% mortality, which increased to 36.67% at 25 μM (Figure 7B). Therefore, we subsequently used 10 μM SB431542 to examine its effect on liver regeneration (Figure 7B). mCherry imaging revealed marked liver regeneration at 72 hours after Mtz removal (Figure 7, C and D). Notably, 10 μM SB431542 markedly inhibited this regenerative response (Figure 7, C and D). These results highlight the essential role of TGF-β signaling in cholangiocyte-mediated liver regeneration after massive hepatocyte loss.

In addition to TGF-β, we assessed the effects of HGF on MHN-associated ALF by treating Mtz-exposed zebrafish with recombinant HGF. Interestingly, HGF treatment reduced zebrafish mortality from 50% (15/30) to 33% (10/30) (Figure 7E), indicating its therapeutic potential in this zebrafish model. These results suggest that both the TGF-β and HGF signaling pathways contribute to the survival of zebrafish with MHN-associated ALF.

To further clarify how TGF-β signaling regulates LPC function and interacts with HGF signaling, we performed RNA-seq on murine LPC line BMOL, which was treated with *siSmad2*, *siSmad3*, *siTgfbr1*, SB431542, HGF, or SB431542+HGF. PCA and correlation heatmaps showed that cells with impaired TGF-β signaling, cells treated with HGF, and controls formed distinct clusters (Figure 7, F and G). Notably, silencing *Smad2*, *Smad3*, or *Tgfbr1* induced distinct transcriptomic changes in BMOL cells, particularly involving the downregulation of liver function genes (Figure 7, H and I). For example, silencing Tgfbr1 reduced transcript levels of Cebpa and Alb, while knockdown of *Smad2* downregulated *Hnf4a*, *Hnf1a*, *Alb*, and *Apob* genes (Figure 7, H and I). Compared with *Tgfbr1* and *Smad2* interference, silencing Smad3 suppressed a broad set of liver function genes, including transcription factors (*Hnf4a*, *Hnf1a*, *Cebpa*, and *Srebf1*) and functional genes (*Alb*, *Apob*, *Gc*, and *Fah*) (Figure 7, H and I). GO biological process enrichment analysis showed that in addition to disrupting TGF-β signaling and response pathways, silencing these TGF-β signaling components in LPCs primarily affected cell cycle, lipid localization and catabolic process, glucose homeostasis, cholesterol transport, and protein secretion (Figure 7J).

Another RNA-seq analysis was conducted to examine transcriptomic alterations in BMOL cells treated with HGF, SB431542, or a combination of both cytokines. As shown in the heatmap, HGF treatment upregulated the expression of several hepatocyte-related genes, including *Hnf4a*, *Hnf1a*, *Fah*, *Srebf1*, *Cebpa*, *Slc23a2*, *Pfkfb1*, *Apob*, and *Gc*. The induction was significantly inhibited when BMOL cells were simultaneously treated with SB431542 (Figure 7K). Violin plots further demonstrated that SB431542 inhibited HGF-induced expression of *Hnf4a*, *Hnf1a*, *Fah*, and *Srebf1* in LPCs (Figure 7L). Subsequently, qRT-PCR confirmed that HGF-induced expression of *Hnf4a* and *Hnf1a* was suppressed when TGF-β signaling was inhibited by SB431542 (Figure 7M). These results indicate that TGF-β signaling is essential for LPC-mediated liver function restoration following MHN. HGF requires intact TGF-β signaling to enable LPCs to perform hepatic function and initiate differentiation toward hepatocytes.

## Discussion

In the current study, we show the following. (i) As in hepatocytes (27), TGF-β inhibits LPC proliferation both in vitro and in DDC-fed mice. Its antiproliferative effect is achieved through SMAD-mediated expression of the CDK inhibitors p15 and p21, which suppress cyclin D1 expression and arrest cell cycle transition from the G1 to the S phase. (ii) In ALF patients with MHN, additional signals from HGF and EGF override the antiproliferative effect of TGF-β and drive LPC proliferation. (iii) Single-cell sequencing data from zebrafish undergoing hepatocyte ablation further demonstrated active TGF-β and HGF signaling. (iv) In addition to inducing LPC proliferation, HGF promoted the expression of vital hepatocyte genes in LPCs and reduced zebrafish mortality following MHN. Notably, the expression of these HGF-induced key hepatocyte genes in LPCs requires TGF-β signaling. These results illustrate the central role of TGF-β in regulating LPC behaviors.

To date, the existence and definition of LPCs have been controversial in the scientific community. LPCs are defined as liver cells, which simultaneously express hepatocyte (*HNF4A*, *HNF1A*, *ALB*, *KRT8*, *KRT18*) and cholangiocyte markers (*EPCAM*, *SOX9*, *KRT7*, *KRT19*), a combination that is rarely observed under physiological conditions. Since Yamanaka and colleagues discovered that mature cells can be reprogrammed to become pluripotent, a clear boundary between somatic cells and stem cells no longer exists (40). However, somatic cells actually do not reprogram spontaneously in vivo due to physiological and pathophysiological limitations.

In adult livers, hepatocytes and cholangiocytes can transdifferentiate into each other depending on disease conditions and repair demands (14). Willenbring and colleagues revealed a critical role of TGF-β in driving hepatocyte differentiation into cholangiocytes using an animal model mimicking biliary Alagille syndrome (29). In contrast with hepatocyte dedifferentiation in cholestasis, MHN-associated ALF destroys the majority of hepatocytes and makes dedifferentiation of mature hepatocytes impossible (41). Multiple experimental studies based on zebrafish with complete hepatocyte ablation clearly show that cholangiocytes transdifferentiate into hepatocytes and thus restore parenchymal mass under the condition of MHN (10, 11, 33).

It is not known to date how LPCs are kept in a physiological state. This study revealed that TGF-β signaling might serve as a key regulator preventing LPC activation in normal conditions. We found that TGF-β impedes LPC cell cycle progression from the G1 to the S phase by upregulating the expression of the genes *CDKN1A* and *CDKN2B*, which encode the 2 CDK inhibitors p15^INK4B^ and p21^Cip1^, respectively. Additionally, TGF-β also represses c-MYC expression in LPCs. As in other cell types (42), the antiproliferative effect of TGF-β in LPCs is reversible. The most robust LPC proliferation occurs in MHN-associated ALF, which is defined as a type III DR by Desmet (14). In this study, spatial transcriptomic analysis revealed that LPCs receive multiple critical signals from surrounding macrophages and HSCs, including antiproliferative mediators such as TGF-β and pro-proliferative factors like HGF and EGF. The activation of HGF/p-MET/p-STAT3 and EGF/p-EGFR/p-ERK signaling pathways in LPCs of patients with ALF was confirmed by IHC and IF staining. Notably, robust expression of p-MET, p-STAT3, p-EGFR, and p-ERK was mainly observed in LPCs, while absent in the remaining hepatocytes of these patients with ALF, suggesting that LPCs are primary cells mediating liver regeneration. In vitro studies further demonstrated that both HGF and EGF are sufficient to induce LPC proliferation, even in the presence of high concentrations of TGF-β. These findings explain why p-SMAD2–positive LPCs still undergo robust proliferation: HGF and EGF override the inhibitory signals to drive cell cycle progression. In a cohort of patients with ALF, HGF concentrations reached 16.4 ± 14.67 ng/mL — more than 60 times the levels observed in healthy controls (0.27 ± 0.08 ng/mL) (38). Therefore, LPCs may predominantly receive HGF stimulation rather than EGF in MHN-associated ALF.

The effects of HGF on hepatocytes, particularly during liver regeneration, have been extensively investigated. Following partial hepatectomy (PHx) in rodents (43, 44), hepatocyte-driven liver regeneration is regulated sequentially by HGF induction and later TGF-β–mediated growth inhibition. In contrast with PHx, residual hepatocytes and LPCs in patients with MHN-associated ALF are exposed simultaneously to both HGF and TGF-β. Notably, our current study reveals that (i) LPCs proliferate despite concurrent exposure to HGF and TGF-β, indicating that HGF-mediated signaling predominates in regulating LPC expansion in ALF; and (ii) residual hepatocytes are exposed primarily to TGF-β and appear insensitive to HGF stimulation. The detailed mechanisms require further investigation.

These observations are supported by single-cell sequencing analysis obtained from a zebrafish model. Since He et al. (11) and Choi et al. (10) demonstrated that nearly all cholangiocytes can transdifferentiate into hepatocytes in zebrafish undergoing hepatocyte ablation, the zebrafish (*Danio rerio*) has emerged as the most valuable model system for studying MHN-induced ALF given that no rodent models are available for this urgent clinical syndrome (12, 34, 45). Two elegant studies confirmed that, consistent with findings from patients with ALF, both TGF-β and HGF signaling are robust in cholangiocytes of fish undergoing hepatocyte ablation (33, 45). Physiologically, TGF-β and HGF are primarily produced by HSCs with TGF-β signals directed to macrophages and HGF signals targeted to hepatocytes. Following hepatocyte ablation, both HSC-derived TGF-β and HGF signals redirected to cholangiocytes, suggesting a critical role of cholangiocyte-derived progenitor-like regenerative state in this urgent scenario. In contrast with TGF-β and HGF, EGF signaling appears to be dispensable in zebrafish.

An interesting question is what role the TGF-β/SMAD signaling pathway plays when its antiproliferative effect on LPCs is compromised in MHN-associated ALF. We observed that, in addition to functioning as a complete mitogen, HGF is a critical growth factor inducing hepatocyte genes such as *HNF4A* in LPCs (39). In this study, we performed 2 experiments to clarify the effects of TGF-β signaling in LPCs. In vivo, we found that the TGF-β receptor inhibitor SB431542 negatively regulates cholangiocyte-mediated liver regeneration in zebrafish undergoing hepatocyte ablation. In the absence of hepatocytes, Mtz-treated fish usually survive by initiating cholangiocyte-mediated liver regeneration (10, 11). However, a high dose of SB431542 results in fish death. In vitro, RNA-seq and PCR analyses further demonstrated that HGF-induced hepatocyte genes in LPCs are markedly inhibited by this TGF-β receptor inhibitor. These findings highlight the essential role of TGF-β/SMAD signaling in MHN-associated ALF, which is consistent with our previous observation — loss of p-SMAD2 expression in LPCs is associated with poor clinical outcome in patients with ALF (46).

In chronic liver diseases (47, 48), HSCs are the major source of TGF-β1. In ALF, Flex analysis showed high levels of TGF-β1 expression in macrophages, neutrophils, endothelial cells, and other inflammatory cell populations, including DCs, NK cells, and B cells. In contrast, scRNA-seq analysis demonstrated that activated HSCs are the primary cellular source of TGF-β1 in the zebrafish model of MHN-associated ALF. This discrepancy between the patient and zebrafish data may be attributable not only to species differences but also to the timing of liver tissue collection. Specifically, the zebrafish liver samples were collected shortly after MHN exposure, whereas the patient liver sample was obtained at least 3 weeks after MHN onset. Therefore, analyses of liver tissues collected from patients with MHN-associated ALF shortly after disease onset would help clarify this discrepancy. Unfortunately, opportunities to obtain such liver tissue samples are extremely limited in clinical practice.

A recent study by Wu and colleagues demonstrates an active role of TGF-β in regulating DDC-induced mouse liver injury and repair (35). They showed that TGF-β signaling is strongly activated in LPCs during liver repair following DDC stimulation. TGF-β ligands (mainly TGF-β2), produced by cholangiocytes, suppress proliferation of periportal hepatocytes during recovery (35). In contrast with the DDC model, in which LPCs act primarily to repair injured bile ducts, the current study highlights the role of TGF-β in severely damaged liver characterized by MHN. After MHN, macrophage-derived TGF-β1, rather than cholangiocyte-produced TGF-β2, functions as the dominant signaling ligand. Interestingly, although TGF-β signaling is highly active, it does not drive LPC differentiation toward cholangiocytes. Instead, it cooperates with HGF to initiate transcription of genes essential for hepatocyte function (e.g., HNF4α and HNF1α), illustrating the classic cell context–dependent mechanisms of TGF-β signaling. Notably, whether derived from macrophages during MHN or from cholangiocytes following DDC stimulation, TGF-β signaling inhibits hepatocyte proliferation but does not suppress LPC proliferation. The underlying mechanisms remain to be investigated.

**Figure 8:**
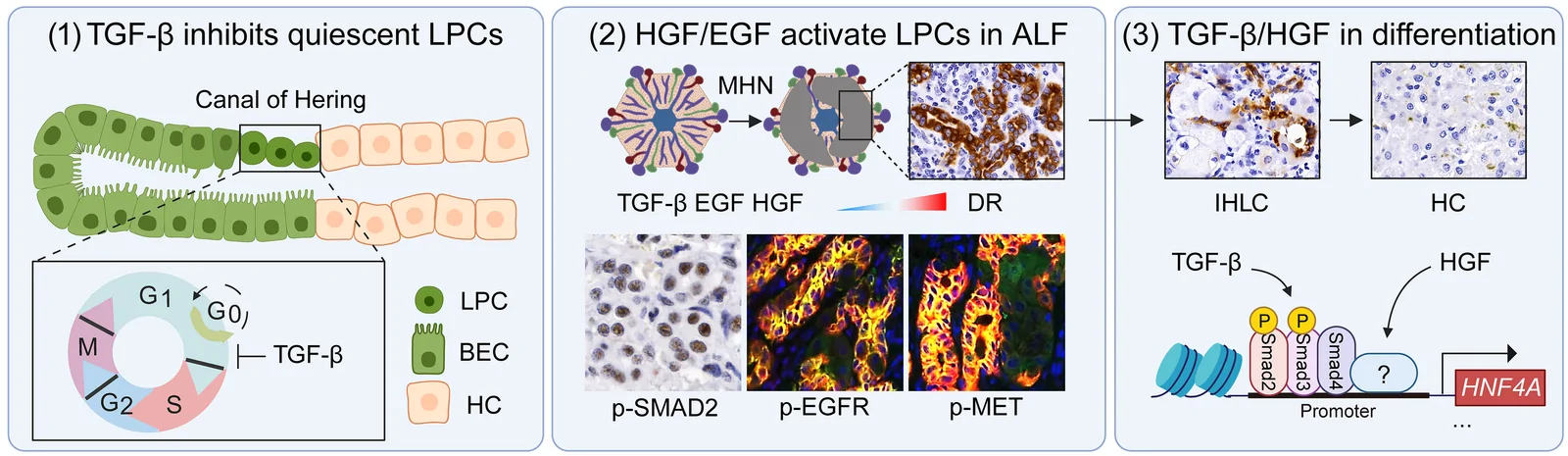
A graphical summary. (i) TGF-β–mediated inhibition of quiescent LPCs, (ii) activation of LPCs by HGF/EGF signaling during MHN, and (iii) coordinated roles of TGF-β and HGF in LPC differentiation toward hepatocytes by regulating transcriptional factors, such as HNF4A. BEC, biliary epithelial cell.

Taken together, although the inhibitory role of TGF-β and the mitogenic role of HGF have long been recognized, this study uncovers an additional mechanism in MHN-associated ALF: TGF-β signaling is not simply suppressive but instead acts as a permissive and integrative signal required for HGF-driven LPC expansion and hepatocyte differentiation in an inflamed regenerative microenvironment. As summarized in Figure 8, TGF-β maintains LPCs in a quiescent state in a normal liver. Once ALF occurs, MHN induces severe inflammatory cell infiltration (e.g., macrophage recruitment) to clear dead hepatocytes and restore local homeostasis, HSC activation for wound repair, and LPC proliferation to restore liver function. Proliferating LPCs produce chemokines that recruit macrophages and activated HSCs. HSCs produce substantial amounts of TGF-β and HGF. HGF further stimulates LPC proliferation and differentiation toward hepatocytes in collaboration with TGF-β. In this scenario, TGF-β serves as an integrative signal that determines LPC functions and fate.

These findings highlight a key role of the microenvironment in determining cellular responses to external signals such as TGF-β. The effects of TGF-β on biliary system development and LPC differentiation have been recognized over the past 2 decades (28, 29, 49). The current study further elucidates how this cytokine contributes to LPC biology. On the one hand, TGF-β alone is insufficient to drive LPC behavior, including proliferation and differentiation (Figure 4). On the other hand, LPC-mediated liver regeneration is largely compromised in the absence of TGF-β signaling (Figure 7). Notably, HGF-driven LPC proliferation and function switching also requires TGF-β–activated SMAD signaling (Figure 7). Collectively, these results suggest that successful therapeutic strategies must account for the coordinated activation of multiple essential signaling pathways, which are profoundly disrupted in MHN-associated ALF. Beyond TGF-β and HGF, which additional signaling pathways are indispensable in LPC-mediated liver regeneration? How does the severely injured liver coordinate these critical signals within a hostile disease microenvironment? Addressing these questions will be essential for developing effective therapies for MHN-associated ALF.

## Methods

### Sex as a biological variable.

This study focused on HBV-associated ALF, a largely sex-independent condition; sex was therefore not treated as a biological variable. Human tissue specimens were obtained from both male and female donors. Male mice were used in an animal model.

### Patients.

A total of 8 patients with ALF were enrolled at the Department of Gastroenterology and Hepatology, Beijing You’an Hospital, affiliated with Capital Medical University, between 2015 and 2018. All patients were diagnosed with HBV-induced ALF, and the liver tissues were collected when patients received liver transplantation. These patients have been investigated in previous studies (46, 50). Tissue samples were fixed in 10% formalin for 24 hours and embedded in paraffin for histological measurement, IF, IHC, and GeoMx DSP analysis.

In this study, ALF is defined as severe acute liver injury lasting more than 4 weeks with coagulation abnormalities (international normalized ratio ≥ 1.5) and any degree of mental alteration (encephalopathy) in a patient without preexisting liver disease.

### Zebrafish ALF model and SB431542 treatment.

To establish an ALF model, *Tg(fabp10a:mCherry-NTR)* zebrafish at 3 days postfertilization (dpf) were treated with 10 mM Mtz (MedChemExpress, MCE) and a series of SB431542 (MCE) concentrations (1.56, 3.12, 6.25, 12.5, and 25.0 μM) via aqueous exposure in embryo medium (3 mL per well, 30 fish per well) at 28°C. After 48 hours of cotreatment (3–5 dpf), Mtz was removed by triple washing. Survival rate and morphological toxicity were monitored daily until 8 dpf under continued SB431542 exposure.

To assess the effect of SB431542 on liver regeneration after Mtz-induced injury, zebrafish were randomly assigned to 3 groups: (i) DMSO, (ii) Mtz + DMSO, and (iii) Mtz + SB431542. After Mtz removal (A48h-R0h), fish were transferred into fresh embryo medium containing either DMSO or 10 μM SB431542 and maintained until 8 dpf. At 0, 6, 24, and 72 hours after Mtz withdrawal (R0h, R6h, R24h, R72h), 10 fish per group were randomly selected for imaging. Liver-specific mCherry fluorescence was visualized using an electric motorized zoom fluorescence stereomicroscope (AZ100, Nikon) and quantified using NIS-Elements D 3.20 software. Liver fluorescence intensity was compared across groups to evaluate regenerative progression. Zebrafish orientation during imaging is illustrated in Supplemental Figure 13.

To examine the effect of HGF on survival, zebrafish were exposed to 15.0 mM Mtz from 3 dpf and intravenously injected with HGF (MCE) at 10 nL × 50 ng/μL at 3, 5, and 7 dpf (*n* = 30 per group). Mortality was monitored daily until 8 dpf and compared between groups using a χ^2^ test.

### GeoMx DSP.

Spatial transcriptomics was performed by GeoMx DSP (NanoString) with the probes of a whole-transcriptome atlas panel. FFPE tissue sections (5 μm) were baked at 60°C for 30 minutes. Subsequently, slides were deparaffinized by xylene, underwent antigen retrieval in Tris-EDTA buffer (pH 9.0, 00-4956-58; Thermo Fisher Scientific) for 20 minutes, and were incubated with 1 μg/mL proteinase K (AM2546; Thermo Fisher Scientific) treatment for 15 minutes at 37°C. After fixation with 10% neutral buffered formalin (15740-04; EMS Diasum) for 5 minutes and WTA probe hybridization at 37°C overnight, sections were stringently washed and stained with morphology markers (CK8/18-AF488, CK7-AF594, CD68-AF647) and nuclear stain SYTO83-CY3 for 1 hour. Based on the fluorescence signals, we defined and selected 4 phenotypically distinct cell type–specific AOIs: CK7^+^ LPCs (*n* = 37), CK8/18^+^ HCs (*n* = 20), α-SMA^+^ HSCs (*n* = 21), and CD68^+^ macrophages (*n* = 37). A total of 115 AOIs were UV-illuminated. The released RNA probes were collected for PCR amplification followed by AMPure XP bead (A63880; Beckman Coulter) purification. The purified libraries were sequenced by NextSeq 550 platform (Illumina) for spatial gene expression analysis.

### GeoMx data analysis.

FASTQ data were converted to DCC files by GeoMx NGS Pipeline software (V3.1.1.6) and analyzed by GeoMx DSP data center software (V3.1.0.222) for quality control and normalization. Quality control was assessed for each AOI to evaluate PCR contamination and sequencing depth. All 18,676 gene-associated probes and AOIs passed quality control. After Q3 normalization, gene expression data were analyzed by R (v4.3.1) with the following packages: limma (v3.58.1) for differential expression analysis, clusterProfiler (v4.10.1) for GSEA, ggplot2 (v3.5.2) for visualization, ComplexHeatmap (v2.25.2) for heatmap generation, and CellChat (v1.6.1) for cell-cell communication analysis. Differential expression analysis between groups was conducted with |log_2_FoldChange| > 0.5 and Benjamini-Hochberg–adjusted *P* < 0.05 as cutoff criteria. Adjusted *P* values less than 0.05 were considered statistically significant.

### Chromium single-cell gene expression Flex (Flex scRNA-seq).

A 50 μm FFPE liver tissue curl was dissociated with Liberase TH Research Grade (Roche Diagnostics, 05401151001) according to the manufacturer’s instructions. Isolated cells were washed, counted, and resuspended, loading approximately 16,000 cells per GEM well, targeting 10,000 recovered cells on a Chromium Chip Q. Sequencing libraries were prepared following the Chromium Fixed RNA Kit User Guide (10x Genomics, catalog 1000474), sequenced on an Illumina NovaSeq X Plus with paired-end dual-indexing, and demultiplexed using bcl2fastq. FASTQ files were processed with Cell Ranger v7.0.1 (10x Genomics) using the multipipeline against the GRCh38-2020-A reference genome.

For downstream analysis, count matrices were imported into R (v4.3.1) and processed with Seurat (v4.3). Low-quality cells were excluded by filtering cells with fewer than 200 or more than 7,000 detected genes and a mitochondrial gene fraction exceeding 10%. Putative doublets were identified and removed using DoubletFinder, with the optimal pK parameter determined by BCmvn maximization and the expected doublet rate estimated as 0.8% per 1,000 cells. Data were normalized using NormalizeData (scale factor = 10,000), followed by identification of 2,000 highly variable genes (VST method), PCA, and UMAP dimensionality reduction using the top 30 principal components. Unsupervised graph-based clustering (resolutio*n* = 0.5) identified 14 major cell populations (clusters 0–13), which were annotated based on canonical marker gene expression. Cholangiocytes, hepatocytes, HSCs/fibroblasts, and macrophages were extracted as a subset for reclustering (resolution = 0.3) and downstream analyses. Cell-cell communication was inferred using CellChat (v1.6.1) with the human ligand-receptor database, applying a truncated mean method (trim = 0.05). Trajectory analysis of hepatocytes and cholangiocytes was performed using Monocle 2 (v2.28.0) with DDRTree dimensionality reduction, using differentially expressed genes identified by Seurat as ordering genes.

### ScRNA-seq data processing and analysis.

Single-cell transcriptomic data from zebrafish liver (GSE272484) were obtained in HDF5-based AnnData format (.h5ad) and imported into R (v4.3.1) using the zellkonverter package (v1.12.1). The dataset includes liver samples collected under homeostatic and injury conditions, including 2 uninjured controls (Control1, Control2), and Mtz-induced ablation at 0, 1, and 9 dppa. For downstream analysis, only the Mtz injury groups and corresponding controls were retained. Hepatocytes, cholangiocytes, HSCs, and macrophages were selected based on canonical marker gene expression.

Raw unique molecular identifier counts were normalized using log normalization with a scale factor of 10,000. Highly variable genes were identified using the “vst” method, followed by data scaling and centering. PCA was performed to reduce dimensionality, and the top 30 principal components were used for batch correction across biological replicates using Harmony (v1.0). Harmony-corrected embeddings were used for UMAP visualization and graph-based clustering (resolution = 0.3). Clusters were manually annotated based on canonical marker gene expression.

Trajectory inference was conducted using Monocle2 (v2.28.0) on HSCs and cholangiocytes. Raw unique molecular identifier count matrices were extracted from Seurat objects and converted into CellDataSet objects. Size factors and dispersion estimates were computed using Monocle’s standard preprocessing pipeline. Highly variable genes identified in Seurat were retained for ordering. Dimensionality reduction was performed using the DDRTree algorithm, and cells were ordered along pseudotime. Lineage progression and pseudotemporal states were visualized by projecting cells in low-dimensional space. The expression dynamics of key hepatic lineage markers (e.g., hnf4a, gc, cebpa) and representative signaling molecules (e.g., tgfb1a, tgfb1b, hgfa, hgfb) were assessed across pseudotime, and unsupervised clustering of temporally regulated genes revealed distinct expression modules.

Cell-cell communication analysis was performed using the CellChat package (v1.6.1). For each condition (Control, Mtz 0 dppa, Mtz 1 dppa, and Mtz 9 dppa), a CellChat object was constructed using log-normalized expression data from the annotated cell populations, including HC, CC, HSC, and macrophage. The zebrafish-specific ligand-receptor database provided in CellChatDB was used as a reference, with manual addition of the HGF signaling axis (hgfa/hgfb–met). Communication probabilities were calculated using the truncated mean method (trim = 0.05), and signaling strength was aggregated at the pathway level. CellChat objects were merged across conditions using mergeCellChat to facilitate comparison. Global and pathway-specific communication networks were visualized using built-in circle plots, bubble plots, and aggregated interaction diagrams.

### RNA-seq data processing and analysis.

Total RNA was extracted from HepaRG and BMOL cells by TRIzol reagent. After RNA quality control, mRNA libraries were prepared by polyA enrichment and sequenced on the NovaSeq X Plus platform. For HepaRG and BMOL cells, raw reads were aligned to the GRCh38 and GRCm39 reference genomes, respectively. Quality control and preprocessing steps included adapter trimming, read quality filtering, and duplicate removal using FastQC, TrimGalore, fq, and Picard. Alignment and quantification were performed using STAR (v2.7.10a) and RSEM (v1.3.1). Additional assessments included transcript integrity evaluation (RSeQC), read distribution (Qualimap), and summary statistics (Samtools and Bedtools).

For downstream analysis, gene-level count matrices were imported into R and analyzed using the DESeq2 package (v1.28.0) to identify differentially expressed genes, using an absolute log_2_ fold-change greater than 1 and an adjusted *P* value less than 0.05 as thresholds. Depending on the analysis purpose, both raw counts and transcripts per million values were used. PCA and Pearson’s correlation heatmaps were used to assess sample similarity. Gene expression dynamics were visualized using volcano plots, violin plots, and heatmaps. The Mfuzz package (v2.58.0) was used for soft clustering of time-resolved expression trends. Functional enrichment analysis was conducted using the clusterProfiler package (v4.10.1), and significantly enriched GO BPs were identified based on a *P* value cutoff of 0.05. Time-course clustering was performed using the ClusterGVis package (v0.1.3) with Mfuzz to visualize dynamic expression patterns, highlighting selected marker genes. GSVA was conducted with hallmark gene sets from MSigDB (v24.1.0) using the GSVA package (v1.50.5) to assess pathway activity, focusing on MYC- and E2F-related signatures. Data visualization was performed using ggplot2 (v3.5.2) and ggpubr (v0.6.0).

### Cells.

HepaRG cells were obtained from Biopredic International and cultured in Williams’ E medium supplemented with 10% heat-inactivated FBS, 5 μg/mL insulin, 50 μM hydrocortisone hemisuccinate, 1% l-glutamine, and 100 U/mL penicillin-streptomycin. BMOL cells were provided by George Yeoh, Harry Perkins Institute of Medical Research and School of Molecular Sciences, The University of Western Australia, Perth, Western Australia, Australia. Cells are maintained in Williams’ E medium supplemented with 10% FBS, 2 mM l-glutamine, 100 U/mL penicillin/streptomycin, and 10 μg/mL insulin (I0516, Sigma-Aldrich). Cells were seeded at a density of 1 × 10^4^ to 5 × 10^4^ cells/cm² and cultured at 37°C in a humidified incubator with 5% CO_2_, with the medium being changed every 48 to 72 hours.

Details of GeoMx DSP spatial transcriptomics, transgenic mouse experiments, zebrafish models, single-cell analyses, RNA-seq, and other methods, as well as reagents used in this study, are provided in the Supplemental Methods and Supplemental Table 1.

### Statistics.

All data were expressed as mean ± SD. Comparisons between 2 independent groups were performed using a 2-tailed Welch’s *t* test. Multiple-group comparisons were performed using 1-way ANOVA followed by Dunnett’s or other specified multiple comparisons tests. Mortality rates were compared using a 2-sided Fisher’s exact test. Statistical significance was defined as *P* < 0.05.

### Study approval.

The study protocol involving ALF patients was approved by local ethics committees of Beijing You’an Hospital, Capital Medical University (Jing-2015-084), and the Medical Faculty Mannheim, Heidelberg University (2017-584N-MA). Written informed consent was obtained from patients or their representatives.

The animal experiments (mouse and zebrafish) were approved by the Laboratory Animal Ethics Committee of Ningbo University (protocol 14106) and conformed to the local guidelines for animal care.

### Data availability.

The sequencing datasets generated during this study, including GeoMx DSP, 10x Genomics Flex scRNA-seq, and bulk RNA-seq of BMOL and HepaRG cells, are available in the NCBI Sequence Read Archive under BioProject accession number PRJNA1305731. Values for all data points shown in graphs and values behind any reported means are reported in the Supporting Data Values file. All raw data related to this study can be obtained by contacting the corresponding author.

## Author contributions

HLW conceived and designed the project. HL, CS, XY, WL, and HD collected the patient samples and performed pathological evaluation. CT, TL, HG, LJ, MZ, JX, and LX undertook experiments. CT, HW, CDLT, and ZG performed bioinformatic analyses. CT and HLW drafted the article. CT, SH, RL, MPE, HD, SD, and HLW discussed the data and edited the article critically.

## Conflict of interest

The authors have declared that no conflict of interest exists.

## Funding support

- Deutsche Forschungsgemeinschaft (grants WE 5009/9-1, WE 5009/12-1, and WE 5009/15-1 to HLW and DO 373/24-1 to SD).
- Chinese-German Cooperation Group (grant GZ 1517 to HLW and HD).
- Chinese-German Mobility Programme (grant M-0099 to SD).
- Bundesministerium für Forschung, Technologie und Raumfahrt-PTJ (FKZ 031L0257A and 031L0314A to SD and SH).
- Ministry of Science, Research and the Arts Baden-Württemberg and the Deutsche Forschungsgemeinschaft (INST 35/1503-1 FUGG).
- China Scholarship Council (grant 202106320043 to CT).
- Heidelberg University to cover part of the publication fee.

## Supplementary Material

Supplemental data

Unedited blot and gel images

Supporting data values

## Version 1 07/28/2026

In-Press Preview

## Version 2 09/15/2026

Electronic publication
