# Supplementary material for: TGF-β serves as a critical signaling determinant of liver progenitor cell activation and function
Source: J Clin Invest. 2026 Jul 28;136(18):e199913. doi: 10.1172/JCI199913 (PMC13574154; doi:10.1172/JCI199913)

Supplementary data

Suppl.-Materials and Methods

Mouse model

Smad7 transgenic mice were described previously (1). Smad7 transgenic mice and their wild-type littermates (n=3 per group, male, 8-10 weeks old) were fed by a diet containing 3,5-diethoxycarbonyl-1,4-dihydrocollidine (DDC, 0.1% wt/wt in Purina 5015 mouse chow; Bio-Serv, Frenchtown, NJ) for 4 weeks to induce LPC proliferation (2). Mice received BrdU injection 2 h before sacrifice. Liver samples from multiple lobes were fixed in 4% buffered formalin for histological analysis.

Cell counting kit-8 assay (CCK-8)

Cell viability was assessed by the Cell Counting Kit-8 (CK04-11, Dojindo Laboratories) according to the manufacturer's instructions. Briefly, cells were seeded in 96-well plates at a density of 3-5×103 cells per well and cultured overnight for attachment. After treatments under experimental conditions, 10 μL CCK-8 solution was added to each well containing 100 μL medium and incubated for 1-4 hours at 37°C. The absorbance was measured at 450 nm by microplate reader (Spark 10M; TECAN).

Colony formation assay

Colony formation ability was evaluated by colony formation assay. Cells were

seeded in 6-well plates at 1000 cells per well and cultured for 2 weeks. Colonies were fixed with 4% paraformaldehyde and stained with 0.1% crystal violet for 20 minutes. Excess stain was gently washed off with distilled water, and then photographed.

Cell cycle assay

Cells were synchronized by serum starvation overnight, followed by 24 h treatment under experimental conditions. After treatment, cells were harvested, fixed in ice-cold 75% ethanol overnight at -20°C, and stained with propidium iodide (50 μg/ml) containing 100 μg/ml RNase A for 1 hour at 37°C. Cell cycle distribution was analyzed using flow cytometry (FACSCanto II TPMA2, BD) with at least 10,000 cells recorded per sample. Data were analyzed by FlowJo software (v10.8.1).

RNA isolation and real-time PCR

Total RNA was isolated from cells using TRIzol reagent (15596018; Invitrogen) according to the manufacturer's instructions, followed by chloroform phase separation (C2432; Sigma-Aldrich), isopropanol (1096342511; VWR) precipitation and 75% ethanol wash. The RNA pellet was dissolved in DEPC treated water (4387937; Thermo Fisher Scientific). RNA concentration was measured by multimode microplate reader (Spark 10M; TECAN). For cDNA synthesis, 1 μg total RNA was reverse transcribed using RevertAid H Minus Reverse Transcriptase (EP0452; Thermo Fisher Scientific). Real-time PCR was

performed on a StepOnePlus system (4376600; Thermo Fisher Scientific) by SYBR Green Master Mix (A25918; Thermo Fisher Scientific) with specific primers. The cycling conditions included initial denaturation at 95°C for 15 minutes, followed by 40 cycles of 95°C for 15 seconds, 60°C for 20 seconds, and 72°C for 20 seconds. Relative gene expression was calculated using the ΔΔCt method. qPCR experiments were performed in compliance with MIQE guidelines.

Chromatin immunoprecipitation

Chromatin immunoprecipitation (ChIP) was performed by SimpleChIP® Enzymatic Chromatin IP Kit (#9003, Cell Signaling Technology) according to the manufacturer's protocol. Briefly, cells were fixed with 1% formaldehyde for 10 minutes at room temperature. Chromatin was fragmented by partial digestion with Micrococcal Nuclease at 37°C for 20 minutes to obtain chromatin fragments of 1-5 nucleosomes. The chromatin was immunoprecipitated with c-Myc antibody (2 μg per sample) overnight at 4°C. After immunoprecipitation, protein G magnetic beads were added and incubated for 2 hours at 4°C. The beads were washed by low salt wash buffer three times and high salt wash buffer once. The protein-DNA complexes were eluted and cross-links were reversed by incubation with 5M NaCl at 65°C for 2 hours. After treatment with RNase A and Proteinase K, DNA was purified using spin columns and analyzed by qPCR.

Immunohistochemistry

Paraffin-embedded tissue sections were deparaffinized by xylene (No. 9713.2, Carl Roth) and rehydrated by graded ethanol (K928.4; Carl Roth). For antigen retrieval, sections were heated in 10 mM citrate buffer (pH 6.0, C2404; Sigma-Aldrich) or 1 mM EDTA (pH 8.4, E9884; Sigma-Aldrich) by microwave (800W, alternating 30-second heating/cooling cycles for 15 minutes). After cooling to room temperature, sections were blocked with blocking peroxide (S200389-2; Dako) and 3% BSA (11930.03; SERVA Electrophoresis GmbH) in PBS (14190094; Thermo Fisher Scientific), then incubated with primary antibody in 0.3% BSA/PBS overnight at 4°C. After returning to room temperature, the sections were incubated with the secondary for 1 hour at room temperature. Immunoreactivity was visualized by 3,3'-diaminobenzidine (D5905; Sigma-Aldrich) with 0.03% hydrogen peroxide (1072102500; Merck), counterstained with hematoxylin (H-3401-500; Vector Laboratories). Finally, sections were dehydrated, mounted with mounting medium (C9368; Sigma-Aldrich), and scanned under microscope (DMRBE; Leica) or whole slide system (Aperio VERSA; Leica). The staining intensity was quantified by measuring the integrated optical density per area (IOD/Area) by Image J software (v1.54d).

Immunofluorescence staining

For immunofluorescence staining (IF), cells on coverslips were fixed with 4%

formaldehyde solution (f1635; Sigma-Aldrich), permeabilized with 0.2% Triton X-100 (X100; Sigma-Aldrich), and blocked with 3% BSA in PBS. For tissue sections, samples were deparaffinized, rehydrated, and subjected to microwave-based antigen retrieval in 10 mM citrate buffer (pH 6.0) or 1 mM EDTA (pH 8.0) at 95-100°C (800W, alternating 30-second heating/cooling cycles for 15 minutes). Both cell and tissue samples were incubated with primary antibodies in 0.3% BSA/PBS overnight at 4°C, followed by secondary antibodies for 1 hour at room temperature. Sections were mounted using Fluoromount-G™ (00-4958-02; Thermo Fisher Scientific) and scanned with confocal microscope (TCS SP8; Leica).

Western blotting

Total proteins were extracted by RIPA buffer containing protease inhibitor (04693132001; Roche) and phosphatase inhibitor cocktail 2 (P5726; Sigma-Aldrich), then quantified by BCA assay (5000116; Bio Rad). Twenty µg protein samples were separated by sodium dodecyl sulfate polyacrylamide electrophoresis at 80V through stacking gel and 120V through separating gel, then transferred to PVDF membranes (10600001; GE Healthcare Life Sciences) at 200mA for 2 hours. Following blocking with 5% BSA in Tris-buffered Saline with Tween (TBST), membranes were incubated with primary antibodies overnight at 4°C and HRP-conjugated secondary antibodies for 1 hour at room temperature. GAPDH were used as negative controls. Protein bands were

visualized using Plus-ECL (NEL103001EA; Perkin Elmer) and detected by the imaging system (Fusion SL4; PEQLAB).

References

1. Feng T, et al. Smad7 regulates compensatory hepatocyte proliferation in damaged mouse liver and positively relates to better clinical outcome in human hepatocellular carcinoma. *Clin Sci (Lond).* 2015;128(11):761-74.
2. Feng D, et al. Interleukin-22 promotes proliferation of liver stem/progenitor cells in mice and patients with chronic hepatitis B virus infection. *Gastroenterology.* 2012;143(1):188-98 e7.

Table S1. Materials, reagents, antibodies, commercial kits, and primers

Chemical or reagents	Company	Cat.No.
10 mM Tris-HCl, 0.05% Tween 20, pH 8.0	Teknova	T1485
10% neutral buffered formalin	Sigma Aldrich	HT501128-4L
100% deionized formamide	neoLab	1103ML500
10X Tris-EDTA pH 9.0	Thermo Fisher	00-4956-58
10X Trypsin-EDTA Solution	Sigma	T4174
20X SSC	Sigma Aldrich	S6639
30% Hydrogen Peroxide	Sigma-Aldrich	1072102500
96% Ethanol	Carl Roth	T171.4
99.8% Ethanol	Carl Roth	K928.4
Acrylamide/Bis solution 29:1	Serva	10687.01
Anhydrous ethanol	Neolab	LC-4045.1
APS	Sigma-Aldrich	A3678
Bovine Serum Albumin	Serva	11930
Chloroform	Sigma-Aldrich	C2432
DEPC-treated water	Thermo Fisher	AM9922
Dimethyl sulfoxide	Sigma-Aldrich	41639
dNTP Mix	Thermo Fisher	R0191
Dual Endogenous Enzyme Blocking	DAKO	S200389-2
Dulbecco's Balanced Salt Solution	Thermo Fisher	14190169
ECL buffer (WB)	Perkin Elmer	NEL103001EA
EDTA	Sigma-Aldrich	E9884
EDTA-Na	Carl Roth	8043.1
FBS	Thermo Fisher	10270106
Fluoromount-G mounting medium	Thermo Fisher	00-4958-02
Formaldehyde solution	Sigma-Aldrich	252549
Glycine	Carl Roth	3790.3
Hematoxylin Counterstain	VectorLaboratories	H-3401-500
Hydrocortisone 21-hemisuccinate sodium salt	Sigma-Aldrich	H2270-100MG
Isopropanol	Carl Roth	9866.1
L-Glutamine 200mM	Sigma-Aldrich	G7513
Methanol	Carl Roth	4627.6
Mounting Medium	Sigma-Aldrich	C9368
Nonidet P40 Substitute	Sigma-Aldrich	11754599001
NuPAGE™ LDS Sample Buffer	Thermo Fisher	NP0007
NuPAGE™ Sample Reducing Agent	Thermo Fisher	NP0009
PBS powder	Bio&Sell	BS.L 182-50
Penicillin Streptomycin	Sigma-Aldrich	P0781
Phosphatase inhibitor cocktail 2	Sigma-Aldrich	P5726
PowerUp SYBR Green Master Mix	Thermo Fisher	A25918
Protease Inhibitor Cocktail	Sigma-Aldrich	S8820
Protein Assay Reagent A	Bio Rad	5000113
Protein Assay Reagent B	Bio Rad	5000114

Protein Assay Reagent S	Bio Rad	5000115
Protein ladder	Thermo Fisher	26619
Proteinase K	Thermo Fisher	AM2546
Random Hexamer	Thermo Fisher	SO142
Recombinant EGF(h)	Thermo Fisher	AF-100-15
Recombinant HGF	MCE	HY-P7121
Recombinant TGFβ1(h)	Thermo Fisher	100-21
RevertAid H Minus Reverse Transcriptase	Thermo Fisher	EP0452
RiboLock RNase Inhibitor	Thermo Fisher	EO0382
RNase A	Thermo Fisher	EN0531
RNase AWAY	Thermo Fisher	7003PK
SB431542	MCE	HY-10431
SDS	Carl Roth	2326.2
Sodium chloride	Carl Roth	3957.2
Stripping buffer	Thermo Fisher	21059
TAE buffer (50X)	Jena Bioscence	BU-119-50
TEMED	Sigma-Aldrich	T9281
Tris base	Carl Roth	5429.5
Triton™ X-100	Sigma-Aldrich	X100
Trizol reagent	Thermo Fisher	15596018
Tween20	Sigma Aldrich	P1379
William’s E medium	Thermo Fisher	A1217601
Xylene	Carl Roth	9713.2

Antibodies for immunoblotting

Antibody	Species	Dilution	Company	Cat.No.
p-RB	Rabbit	1:1000	Cell Signaling	8516T
RB	Mouse	1:1000	Cell Signaling	9309T
p15	Rabbit	1:1000	Abcam	ab53034
p21	Mouse	1:500	Santa Cruz Biotechnology	sc-471
Cyclin D1	Rabbit	1:1000	Abcam	ab16663
p-SMAD2	Rabbit	1:1000	Cell Signaling	3108T
p-SMAD3	Rabbit	1:1000	Abcam	ab52903
SMAD2/3	Rabbit	1:1000	Cell Signaling	5678S
c-MYC	Rabbit	1:1000	Abcam	ab32072
p-FOXO1	Rabbit	1:1000	Cell Signaling	9461S
FOXO1	Rabbit	1:1000	Cell Signaling	2880S
p-AKT	Rabbit	1:1000	Cell Signaling	4060T
AKT	Rabbit	1:1000	Cell Signaling	9272S
p-ERK	Rabbit	1:1000	Cell Signaling	4370T
ERK	Rabbit	1:1000	Cell Signaling	4695T
p-MET	Rabbit	1:1000	Thermo Fisher	44-888G
MET	Mouse	1:1000	Cell Signaling	3127s
c-FOS	Rabbit	1:1000	Cell Signaling	2250S
GAPDH	Mouse	1:2000	Santa Cruz Biotechnology	sc-32233
Anti-Rabbit IgG	Mouse	1:2500	Santa Cruz Biotechnology	sc-2357

Anti-Mouse IgG	Goat	1:2500	Santa Cruz Biotechnology	sc-2005

Antibodies for immunohistochemistry

Antibody	Species	Dilution	Company	Cat.No.
p-SMAD2	Rabbit	1:100	IBL	28027
CK7	Mouse	1:200	DAKO	M7018
CK19	Mouse	1:200	DSHB	TROMA-III
CD68	Mouse	1:200	Thermo Fisher	14-0688-82
c-MYC	Rabbit	1:1000	Proteintech	67447-1-Ig
p-ERK	Rabbit	1:200	Cell Signaling	4370T
p-STAT3	Rabbit	1:200	Cell Signaling	9145T
Anti-Rabbit/HRP	Swine	1:200	DAKO	P021702-2
Anti-Mouse/HRP	Goat	1:200	DAKO	P0447

Antibodies for immunofluorescence

Antibody	Species	Dilution	Company	Cat.No.
Ki-67	Rabbit	1:200	Abcam	ab16667
p-SMAD3	Rabbit	1:200	Abcam	ab118825
p-MET	Rabbit	1:200	Thermo Fisher	44-888G
p-EGFR	Rabbit	1:200	Cell Signaling	3777T
aSMA	Rabbit	1:200	Abcam	ab5694
TGF-β	Mouse	1:100	Santa Cruz	sc-31609
DRAQ5		1:1000	Cell Signaling	4084L
Alexa Fluor 488 Donkey anti-mouse	Donkey	1:200	Jackson Immunoresearch	715-546-150
Alexa Fluor Cy3 Donkey anti-Rabbit	Donkey	1:200	Jackson Immunoresearch	711-166-152

Antibodies for GeoMx staining

Antibody	Species	Dilution	Company	Cat.No.
CK8/18 Alexa Fluor® 488	Mouse	1:100	Bio-Techne	NBP2-34655AF488
CK7 Alexa Fluor® 594	Rabbit	1:100	Abcam	ab203427
CD68 Alexa Fluor® 647	Mouse	1:100	Santa Cruz	sc-20060 AF647
α-SMA Alexa Fluor® 647	Rabbit	1:100	Abcam	ab267537
SYTO83		1:15000	Thermo Fisher	S11364

Antibodies for ChIP assay

Antibody	Species	Dilution	Company	Cat.No.
c-MYC	Rabbit	1:50	Abcam	ab32072
IgG Control	Rabbit	2.5 μg/ml	Cell Signaling	CST#3900S

Critical commercial assays

Kit	Company	Cat.No.
Bio-Rad protein assay kit	Bio Rad	5000113-115
Caspase-3 activity assay	Beyotime	C1115

SYBR Green Master Kit	Thermo Fisher	A25918
GeoMx RNA Slide Prep Kit	Nanostring	GMX-PREP-RNA-FFPE-12
GeoMx DSP Instrument Buffer Kit	Nanostring	GMX-DSP-BUFF-KIT
GeoMx DSP Collection Plate Kit	Nanostring	GMX-DSP-COLL-PLT
GeoMx Hyb Code Pack: RNA	Nanostring	GMX-RNA-HYB-96
Human Whole Transcriptome Atlas	Nanostring	GMX-RNA-H-WTA
Nextseq 500/550 High Output Kit v2.5 kit	Nanostring	20024906
High Sensitivity DNA ScreenTape Analysis	Agilent	5067-5584
Qubit™ dsDNA Quantification Assay Kits	Thermo Fisher	Q32851
SimpleChIP® Enzymatic Chromatin IP Kit	Cell signaling	9003S

Primer for qRT-PCR

Gene	Forward primers (5'-3')	Reverse primers (5'-3')
PPIA	GGCAAATGCTGGACCCAACACA	TGCTGGTCTTGCCATTCCTGGA
GAPDH	GTCTCCTCTGACTTCAACAGCG	ACCACCCTGTTGCTGTAGCCAA
CCND1	TCTACACCGACAACTCCATCCG	TCTGGCATTTTGGAGAGGAAGTG
CDKN2B	ACGGAGTCAACCGTTTCGGGAG	GGTCGGGTGAGAGTGGCAGG
CDKN1A	AGGTGGACCTGGAGACTCTCAG	TCCTCTTGGAGAAGATCAGCCG
MKI67	GAAAGAGTGGCAACCTGCCTTC	GCACCAAGTTTTACTACATCTGCC
MYC	CCTGGTGCTCCATGAGGAGAC	CAGACTCTGACCTTTTGCCAGG
EGFR	AACACCCTGGTCTGGAAGTACG	TCGTTGGACAGCCTTCAAGACC
MET	TGCACAGTTGGTCCTGCCATGA	CAGCCATAGGACCGTATTTCGG
HNF4A	GGTGTCCATACGCATCCTTGAC	AGCCGCTTGATCTTCCCTGGAT
HNF1A	AGACGCTAGTGGAGGAGTGCAA	GGCAAACCAGTTGTAGACACGC
CPS1	CTAGCCTGGATTACATGGTCACC	CCTCAAAGGTACGACCAATAGCC
APOB	AGAGGACAGAGCCTTGGTGGAT	CTGGACAAGGTCATACTCTGCC
F7	GGAACAGTGCTCCTTTGAGGAG	GACTTGAGATGATCCTGGCAGG
F10	TGGTGGAACCATTCTGAGCGAG	CGGTTGTGCTTGATGACCACCT
Ppia	CATACAGGTCCTGGCATCTTGTC	AGACCACATGCTTGCCATCCAG
Gapdh	CATCACTGCCACCCAGAAGACTG	ATGCCAGTGAGCTTCCCGTTCAG
Hnf1a	AGAGACCTTGGTGGAGGAGTGT	GGCAAACCAGTTGTAGACACGC
Hnf4a	TGCGAACTCCTTCTGGATGACC	CAGCACGTCCTTAAACACCATGG
Myc	TCGCTGCTGTCCTCCGAGTCC	GGTTTGCCTCTTCTCCACAGAC
Ccnd1	GCAGAAGGAGATTGTGCCATCC	AGGAAGCGGTCCAGGTAGTTCA
Cdkn1a	TCGCTGTCTTGCACTCTGGTGT	CCAATCTGCGCTTGGAGTGATAG
Cdkn2b	ATCCCAACGCCCTGAACCGCT	AGTTGGGTTCTGCTCCGTGGAG
Mki67	GAGGAGAAACGCCAACCAAGAG	TTTGTCCTCGGTGGCGTTATCC

Primer for ChIP qRT- PCR

Gene	Forward primers (5'-3')	Reverse primers (5'-3')
CCND1 (-247~-31bp)	CTCTGCCGGGCTTTGATCTT	GCTGTGGGTCCTGGCTG
CCND1 (-797~-593bp)	GTGCGCCGGAATGAAACTTG	GACACCCCATATCCAAGCCG
GAPDH	GCGTGTAAGGGTCCCCGTCCT	GTTCAACTGGGCACGCACCGA

Figure legends

Figure S1. Macrophages and activated hepatic stellate cells dominate the ductular reaction microenvironment in acute liver failure patients. (A) Hematoxylin and eosin (HE), immunohistochemistry (IHC), and immunofluorescence (IF) were performed on liver tissues collected from 8 irreversible acute liver failure (ALF) patients who underwent liver transplantation. **(B)** A representative HE staining image shows an area with massive hepatocyte loss. A frame highlights ductular reaction (DR) regions, where proliferative liver progenitor cells (LPCs) are surrounded by dense inflammatory cell infiltration. **(C)** IHC staining for CD68 and α-SMA was performed to highlight macrophages (Mø) and activated HSCs in DR regions. **(D)** IHC staining for CK7 and CK19 was performed to demonstrate the transition from DR and intermediate hepatocyte-like cells (IHLCs) to mature hepatocytes (HCs). **(E)** Co-IF staining for CK7 (yellow), CD68 or α-SMA (red), CK8/18 (green), and nuclei (SYTO83, blue) was used to identify LPCs, macrophages, activated hepatic stellate cells (HSCs), HCs hepatocytes in ALF patients. Scale bars: 100 μm (B–D, low magnification); 25 μm (B–E, high magnification).

Figure S2. GeoMx Digital Spatial Profiling (DSP) workflow for cell-type-specific transcriptomic analysis of liver tissue. (A) Schematic overview of the GeoMx DSP workflow. Tissue sections are hybridized with UV-photocleavable oligonucleotide-tagged RNAscope probes and stained with fluorescent morphology markers. Regions of interest (ROIs) are selected based on tissue morphology. UV light cleaves photocleavable oligonucleotides within selected ROIs, and the released oligos are collected and dispensed into a 96-well plate, followed by next-generation sequencing (NGS). **(B)** Representative fluorescence images illustrating how a digital micromirror device (DMD) directs UV light to select cell-type-specific areas of illumination (AOIs) within a user-defined ROI. A liver tissue section stained for CK8/18 (hepatocytes, HC), CK7 (liver progenitor cells, LPC), CD68 (macrophages, Mø), and SYTO83 (DNA) is shown. Within the ROI (white boundary), $CK7^+$ areas (AOI1, gray mask) and $CD68^+$ areas (AOI2, gray mask) were independently illuminated to cleave and collect cell-type-specific oligonucleotides for RNA profiling. Insets show high-magnification views of representative AOI-selected cells. Scale bars, 50 μm (top) and 10 μm (bottom).

Figure S3. GSEA identifies downregulation of hepatic metabolic and functional pathways in LPCs. (A) GSEA was performed to compare the transcriptomic profiles of LPCs and HCs. Enrichment plots (left panels) illustrate curated gene sets related to liver-specific functions. Normalized enrichment scores (NES) and adjusted *p* values are provided for each comparison. The right panels display heatmaps of representative genes within each pathway, with z-score normalization applied across samples. **(B)** Representative GeoMx digital spatial profiling image of AOI3, showing co-staining for CK8/18 (hepatocytes, green), CK7 (LPCs, yellow), α-SMA (HSCs, magenta), and SYTO83 (DNA, blue). The dashed white circles indicate segmented AOIs used for

spatial transcriptomic profiling. Scale bar, 50 μm. **(C)** Representative multiplexed immunofluorescence images of peri-necrotic regions in MHN-associated ALF liver, showing $CK7^+$ LPC clusters (yellow) surrounded by $CD68^+$ macrophages (red, left) and α-SMA^+ activated HSCs (magenta, right). CK8/18 (hepatocytes, green) and SYTO83 (DNA, blue) are shown in both panels. Scale bar, 200 μm.

Figure S4. Single-cell transcriptomic analysis of MHN-associated ALF patient livers identifies HSCs and macrophages as the predominant sources of HGF and TGF-β1, respectively. (A) Liver tissues from a MHN-ALF patient were subjected to FLEX scRNA-seq. **(B)** UMAP visualization of all cells is shown, colored by annotated cell types, including B cells, DC, cholangiocytes, endothelial cells, HSCs, hepatocytes, lymphatic endothelial cells, macrophages, neutrophils, T/NK cells, plasma cells, proliferating cells and VSMC. **(C)** Expression of representative marker genes across all annotated cell types is displayed as a dot plot. Dot size indicates the percentage of cells expressing each gene, and color intensity reflects average expression level. **(D)** Four cell populations—cholangiocytes, hepatocytes, HSCs, and macrophages—are highlighted in a UMAP and selected for downstream signaling analysis. **(E)** Expression of canonical marker genes for cholangiocytes (*KRT7*, *EPCAM*, *CFTR*, *SPP1*) and hepatocytes (*APOA1*, *APOA2*, *HP*, *TTR*) is shown as violin plots. **(F)** Expression of hepatocyte transcription factors (*HNF4A*, *HNF1A*, *FOXA1*, *FOXA2*, *FOXA3*, *CEBPA*, *ONECUT1*, *PROX1*, *SOX9*) across the four cell populations is presented as a dot plot and heatmap. Notably, a subset of cholangiocytes expressed HNF4A and HNF1A, consistent with hepatocyte-like gene program acquisition in LPCs undergoing differentiation toward hepatocytes in MHN-associated ALF.

Figure S5. Macrophage subclustering and ligand expression profiling in the 10x FLEX single-cell RNA-seq dataset. (A) UMAP plot shows three macrophage subsets identified by unsupervised subclustering: resident Kupffer cells, recruited monocyte-derived macrophages, and an inflammatory subset. **(B)** Subset-defining marker genes (C1QA, CD5L, VSIG4 for Kupffer cells; S100A8, S100A9, VCAN for recruited monocyte-derived macrophages; NFKBIA, NLRP3, CXCL2 for the inflammatory subset) are displayed as a dot plot. **(C)** Expression of TGF-β family ligands (TGFB1, TGFB2, TGFB3) and HGF across all hepatic cell types is shown as a dot plot. **(D)** UMAP feature plots show the expression of TGFB1 and HGF within the macrophage compartment.

Figure S6. TGF-β signaling is essential for suppressing LPCs proliferation in vivo and in vitro. (A) Quantification of p-SMAD3 immunofluorescence intensity (IOD) was performed in liver tissues from wild-type (WT) and Smad7 transgenic (TG) mice fed with DDC for 4 weeks. **(B)** CK19 immunohistochemical staining of liver sections from wild-type and TG(*SMAD7*) mice fed a DDC diet for 4 weeks. Left panels show

representative CK19 staining images, and right panels show digital quantification of CK19-positive areas (highlighted in red). Scale bars, 50 μm. **(C)** Quantification of CK19 expression was performed using integrated optical density (IOD) normalized to tissue area. **(D)** Quantification of CK19 immunofluorescence intensity (IOD) was performed in liver tissues from WT and TG(*Smad7*) mice fed with DDC for 4 weeks. **(E)** Cell viability was analyzed in BMOL cells treated with different concentrations of TGF-β1 for 48 hours using the CCK-8 assay. **(F)** Caspase-3 activity in HepaRG cells treated with or without TGF-β1, normalized to untreated controls. **(G)** qRT-PCR analysis of *Ccnd1*, *Cdkn2b*, and *Cdkn1a* mRNA expression was performed in BMOL cells treated with TGF-β1 for the indicated durations. **(H)** Quantification of Ki-67 immunofluorescence intensity (IOD) was performed in HepaRG cells treated with the indicated concentrations of TGF-β1. **(I)** Representative immunofluorescence images and quantification of Ki-67 (red) and Draq5 (blue) in BMOL cells treated with or without TGF-β1. Scale bar: 50 μm. **(J)** Quantification of p-SMAD2/SMAD2, p-SMAD3/SMAD3, p-RB/RB, p15, p21, and Cyclin D1 protein ratios in BMOL cells at the indicated time points following TGF-β1 treatment. Data are presented as mean ± SD, and statistical significance is indicated as follows: $p < 0.05$ (*), $p < 0.01$ (**), $p < 0.001$ (***), $p < 0.0001$ (****); ns, not significant. 1-way ANOVA with Dunnett's test (**E**, **J**); 2-tailed Welch's t test (**A**, **C**, **D**, **F**, **G**, **H**, **I**).

Figure S7. c-Myc expression and ERK phosphorylation in the livers of ALF patients. (A, B) IHC staining for c-MYC and p-ERK was performed on liver tissues from eight ALF patients. Each patient sample includes an overview image and areas with higher magnification. Scale bars: 500 μm and 50 μm.

Figure S8. HGF promotes LPC proliferation. (A) CCK-8 assay was performed in BMOL cells treated with different concentrations of HGF for 24 hours. **(B)** Colony formation assay was performed in BMOL cells exposed to various concentrations of HGF for 14 days. **(C)** qRT-PCR analysis of *Cdkn2b*, *Cdkn1a*, *Ccnd1*, and *Mki67* mRNA expression was performed in BMOL cells treated with HGF for the indicated durations. **(D)** Quantification of p-MET/MET, p-AKT/AKT, p-ERK/ERK, p-FOXO1/FOXO1, C-MYC, C-JUN, C-FOS, Cyclin D1, and p-RB/RB protein ratios in BMOL cells at the indicated time points following HGF treatment. Data are presented as mean ± SD, and statistical significance is indicated as follows: $p < 0.05$ (*), $p < 0.01$ (**), $p < 0.001$ (***), $p < 0.0001$ (****); ns, not significant. 1-way ANOVA with Dunnett's test (**A**, **D**); 2-tailed Welch's t test (**C**).

Figure S9. HGF overrides TGF-β and promotes LPC proliferation. (A) Colony formation assay was performed in BMOL cells treated with TGF-β1, HGF, or a combination of both for 14 days. **(B)** Quantification of Ki-67 immunofluorescence intensity (IOD) was performed in HepaRG cells treated with TGF-β1, HGF, or a

combination of both. **(C)** Representative immunofluorescence images and quantification of Ki-67 (red) and Draq5 (blue) in BMOL cells treated with TGF-β1, HGF, or a combination of both. Scale bar: 50 μm. **(D)** Flow cytometric analysis of cell cycle distribution in BMOL cells treated with TGF-β1, HGF, or a combination of both. **(E)** qRT-PCR analysis of CDKN1A and CDKN2B mRNA expression in HepaRG cells treated with TGF-β1, HGF, or a combination of both. Data are presented as mean ± SD, and statistical significance is indicated as follows: *p* < 0.05 (**), p < 0.01 (**), p < 0.001 (****); ns, not significant. 2-tailed Welch's t test (**B**-**E**).

Figure S10. EGF overrides TGF-β and promotes LPC proliferation. (A) Cell viability was assessed in HepaRG and BMOL cells treated with different concentrations of EGF for 48 h using the CCK-8 assay. **(B)** A colony formation assay was performed in HepaRG and BMOL cells treated with the indicated concentrations of EGF for 14 days. **(C)** qPCR was used to measure MYC/Myc and CCND1/Ccnd1 mRNA expression in HepaRG cells treated with 1 ng/mL EGF for 0, 1, 3, 6, 9, and 24 h and in BMOL cells treated for 0, 1, 8, and 24 h. **(D)** Western blotting was performed to examine phosphorylated and total ERK, AKT, FOXO1, RB, c-FOS, c-MYC, and Cyclin D1 in HepaRG cells treated with 1 ng/mL EGF at the indicated time points. **(E)** Quantification of p-ERK/ERK, p-AKT/AKT, p-FOXO1/FOXO1, p-RB/RB, c-FOS, c-MYC, and Cyclin D1 protein ratios in BMOL cells at the indicated time points following EGF treatment. **(F)** ChIP-qPCR was used to analyse c-Myc binding to two promoter regions of the *CCND1* gene (~247 bp to ~31 bp and ~797 bp to ~593 bp) in HepaRG cells with EGF treatment for 1h and 8 h. Data are presented as mean ± SD, and statistical significance is indicated as follows: *p* < 0.05 (**), p < 0.01 (**), p < 0.001 (****), *p* < 0.0001 (****); ns, not significant. 1-way ANOVA with Dunnett's test (A, E); 2-tailed Welch's t test (**C**, **F**).

Figure S11. EGF overrides TGF-β and promotes LPC proliferation. (A) Flow cytometry was performed to analyze cell cycle distribution in HepaRG and BMOL cells treated with TGF-β1 (5 ng/mL), EGF (1 ng/mL), or both for 24 h. The right panel summarizes the percentage of cells in the G0/G1, S, and G2/M phase. **(B)** A colony formation assay was performed in HepaRG and BMOL cells treated with TGF-β1, EGF and TGF-β1+EGF for 14 days. **(C)** Immunofluorescence (IF) staining for Ki-67 and Draq5 was performed in HepaRG and BMOL cells under the TGF-β1, EGF and TGF-β1+EGF 24h treatment. (**D**) qRT-PCR analysis of *CDKN1A* and *CDKN2B* mRNA expression in HepaRG cells treated with TGF-β1, EGF, or a combination of both. Scale bars: 50 μm. Data are presented as mean ± SD, and statistical significance is indicated as follows: *p* < 0.05 (**), p < 0.01 (**), p < 0.001 (****), *p* < 0.0001 (****); ns, not significant. 2-tailed Welch's t test (**A**, **C**, **D**)

Figure S12. Knockdown of *EGFR* or *MET* differentially affects LPC proliferation and hepatocyte differentiation markers. (A) qRT-PCR analysis of *EGFR*, *MET*,

MKI67, *HNF4A*, *CPS1*, *APOB*, *F7*, and *F10* mRNA expression in HepaRG cells transfected with *EGFR* siRNA (si*EGFR*) or control siRNA (siCon). **(B)** qRT-PCR analysis of *MET*, *EGFR*, *MKI67*, *HNF4A*, *CPS1*, *APOB*, *F7*, and *F10* mRNA expression in HepaRG cells transfected with *MET* siRNA (si*MET*) or control siRNA (siCon). Data are presented as mean ± SD, and statistical significance is indicated as follows: $p < 0.05$ (*), $p < 0.01$ (**), $p < 0.001$ (***), $p < 0.0001$ (****); ns, not significant. 2-tailed Welch's t test (**A**, **B**).

Figure S13. Orientation and liver localization for fluorescence imaging in zebrafish larvae.

Representative lateral view of a *Tg(fabp10a:mCherry-NTR)* zebrafish larva at 6 dpf from the DMSO control group. Liver-specific mCherry fluorescence is visible in the ventral-anterior region and outlined by a white dashed line. The liver is located posterior to the eye and adjacent to the yolk extension. This orientation was used throughout the study for consistent liver imaging and quantification. Scale bar, 200 μm.

Figure S1

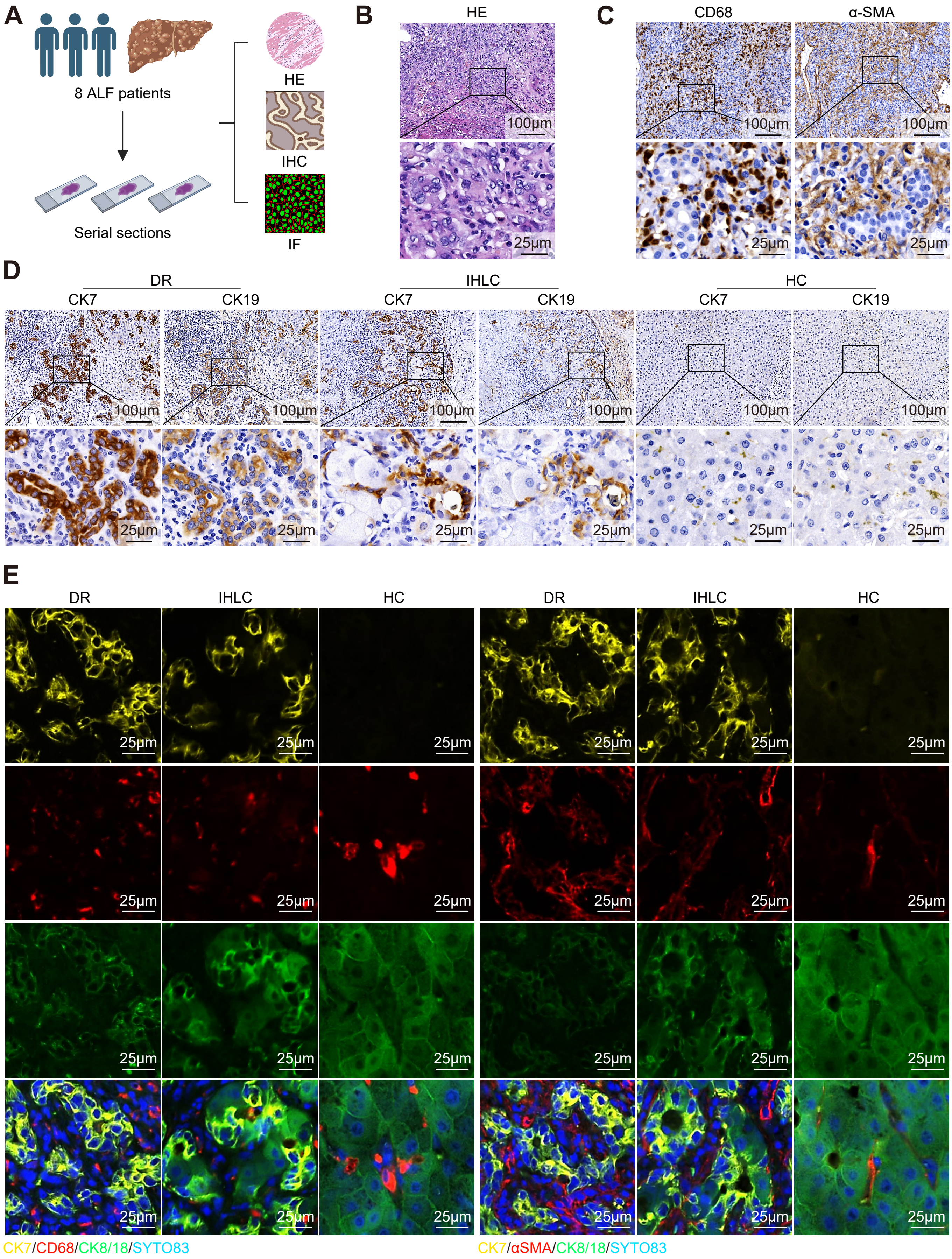

A
8 ALF patients
Serial sections
HE
IHC
IF
B
HE
100μm
25μm
C
CD68
α-SMA
100μm
25μm
D
DR
IHLC
HC
CK7
CK19
100μm
25μm
E
DR
IHLC
HC
25μm
CK7/CD68/CK8/18/SYTO83
CK7/αSMA/CK8/18/SYTO83

Figure S2

A

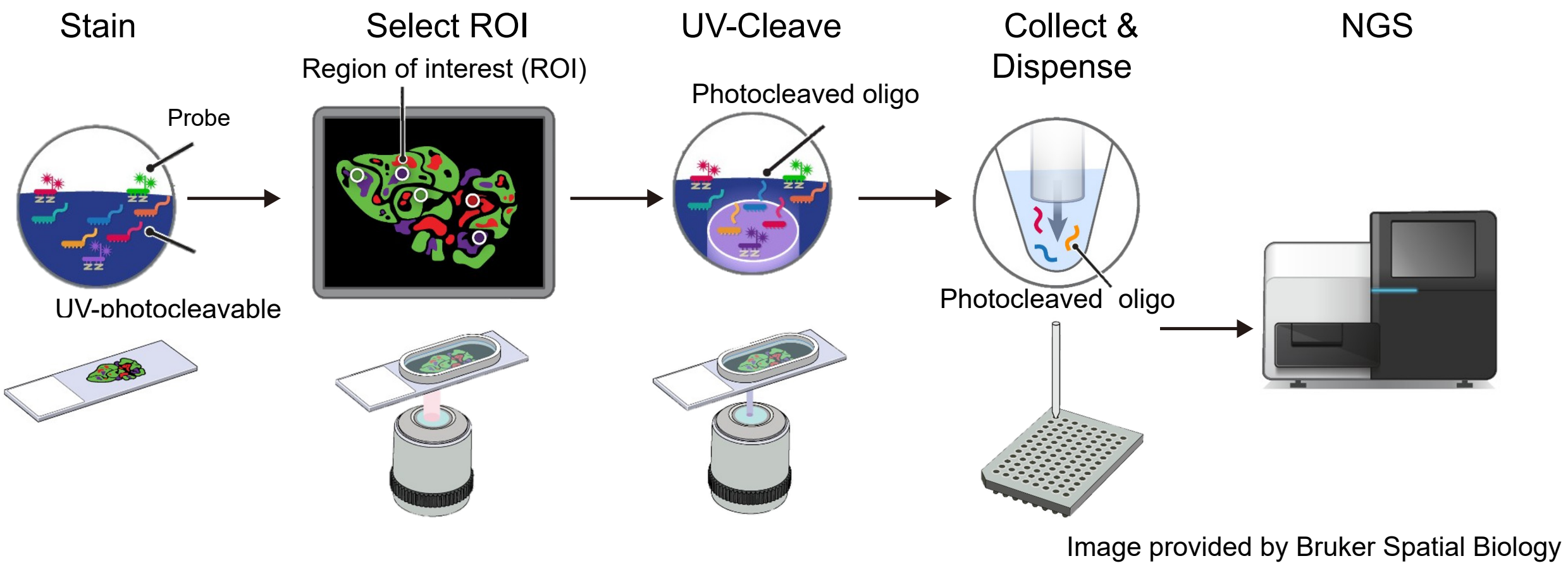

Stain
Select ROI
UV-Cleave
Collect &
Dispense
NGS
Region of interest (ROI)
Probe
Photocleaved oligo
UV-photocleavable
Photocleaved oligo
Image provided by Bruker Spatial Biology

B

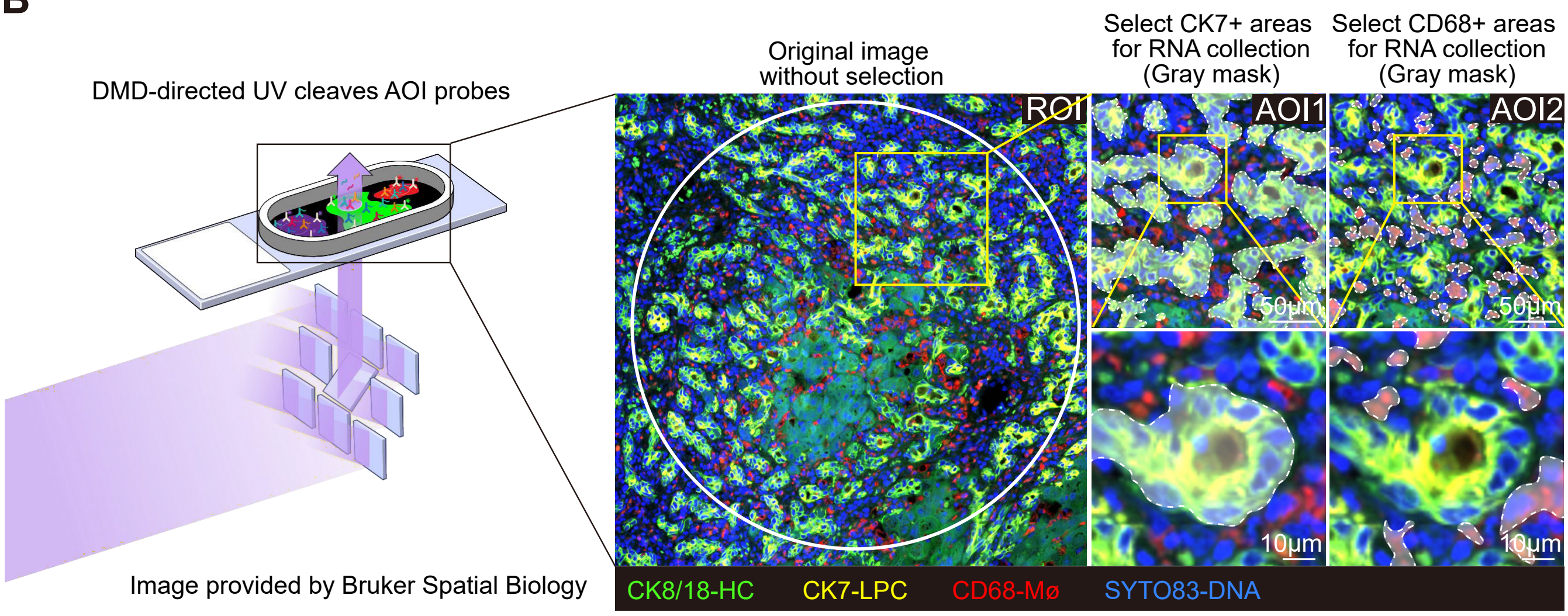

DMD-directed UV cleaves AOI probes
Original image
without selection
Select CK7+ areas
for RNA collection
(Gray mask)
Select CD68+ areas
for RNA collection
(Gray mask)
ROI
AOI1
AOI2
50μm
50μm
10μm
10μm
Image provided by Bruker Spatial Biology
CK8/18-HC
CK7-LPC
CD68-Mø
SYTO83-DNA

Figure S3

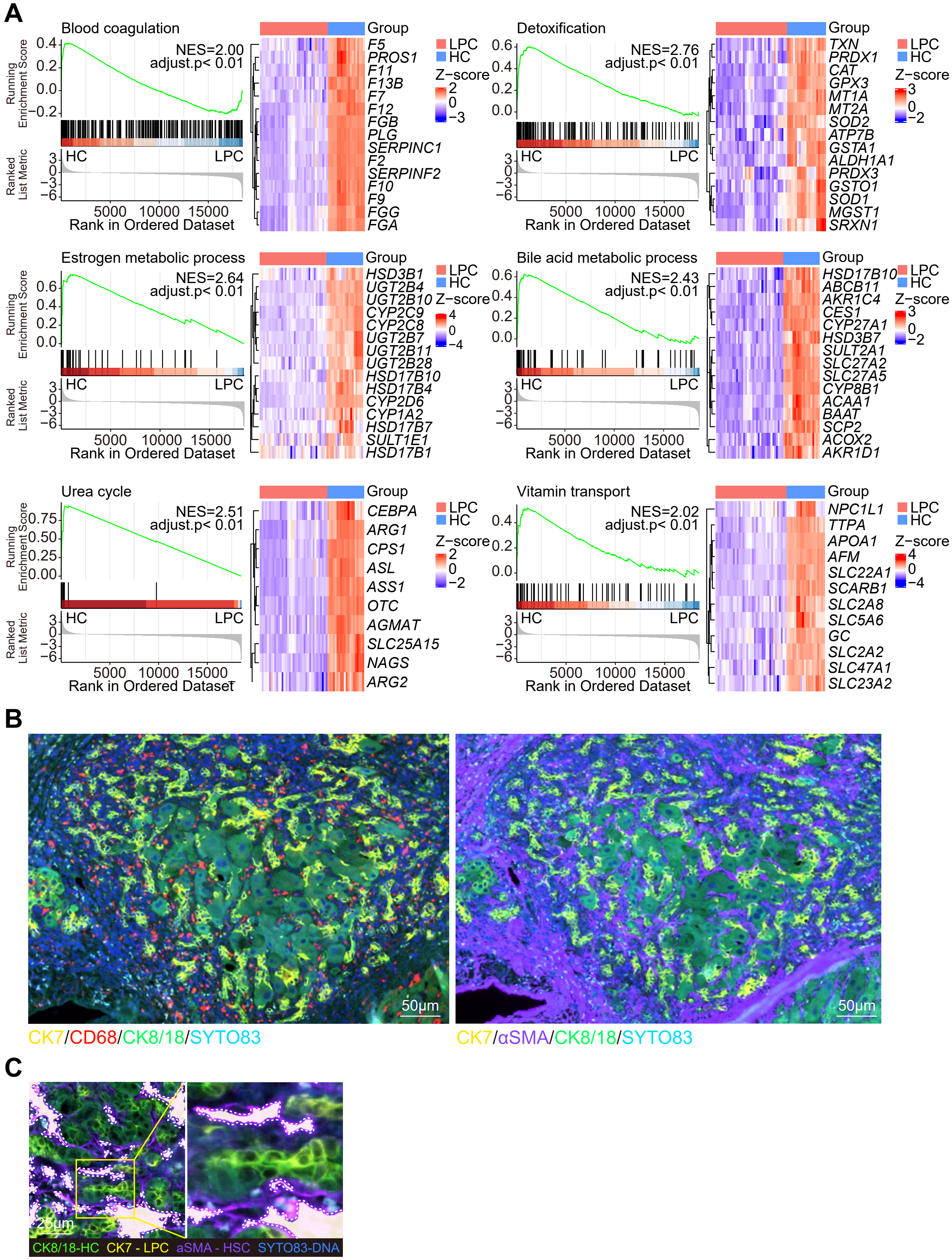
A
Blood coagulation
NES=2.00
adjust.p< 0.01
Running Enrichment Score
Ranked List Metric
HC
LPC
Rank in Ordered Dataset
Group
LPC
HC
Z−score
F5
PROS1
F11
F13B
F7
F12
FGB
PLG
SERPINC1
F2
SERPINF2
F10
F9
FGG
FGA
Detoxification
NES=2.76
adjust.p< 0.01
TXN
PRDX1
CAT
GPX3
MT1A
MT2A
SOD2
ATP7B
GSTA1
ALDH1A1
PRDX3
GSTO1
SOD1
MGST1
SRXN1
Estrogen metabolic process
NES=2.64
adjust.p< 0.01
HSD3B1
UGT2B4
UGT2B10
CYP2C9
CYP2C8
UGT2B7
UGT2B11
UGT2B28
HSD17B10
HSD17B4
CYP2D6
CYP1A2
HSD17B7
SULT1E1
HSD17B1
Bile acid metabolic process
NES=2.43
adjust.p< 0.01
HSD17B10
ABCB11
AKR1C4
CES1
CYP27A1
HSD3B7
SULT2A1
SLC27A2
SLC27A5
CYP8B1
ACAA1
BAAT
SCP2
ACOX2
AKR1D1
Urea cycle
NES=2.51
adjust.p< 0.01
CEBPA
ARG1
CPS1
ASL
ASS1
OTC
AGMAT
SLC25A15
NAGS
ARG2
Vitamin transport
NES=2.02
adjust.p< 0.01
NPC1L1
TTPA
APOA1
AFM
SLC22A1
SCARB1
SLC2A8
SLC5A6
GC
SLC2A2
SLC47A1
SLC23A2
B
50μm
CK7/CD68/CK8/18/SYTO83
CK7/αSMA/CK8/18/SYTO83
C
25μm
CK8/18-HC CK7 - LPC aSMA - HSC SYTO83-DNA

Figure S4

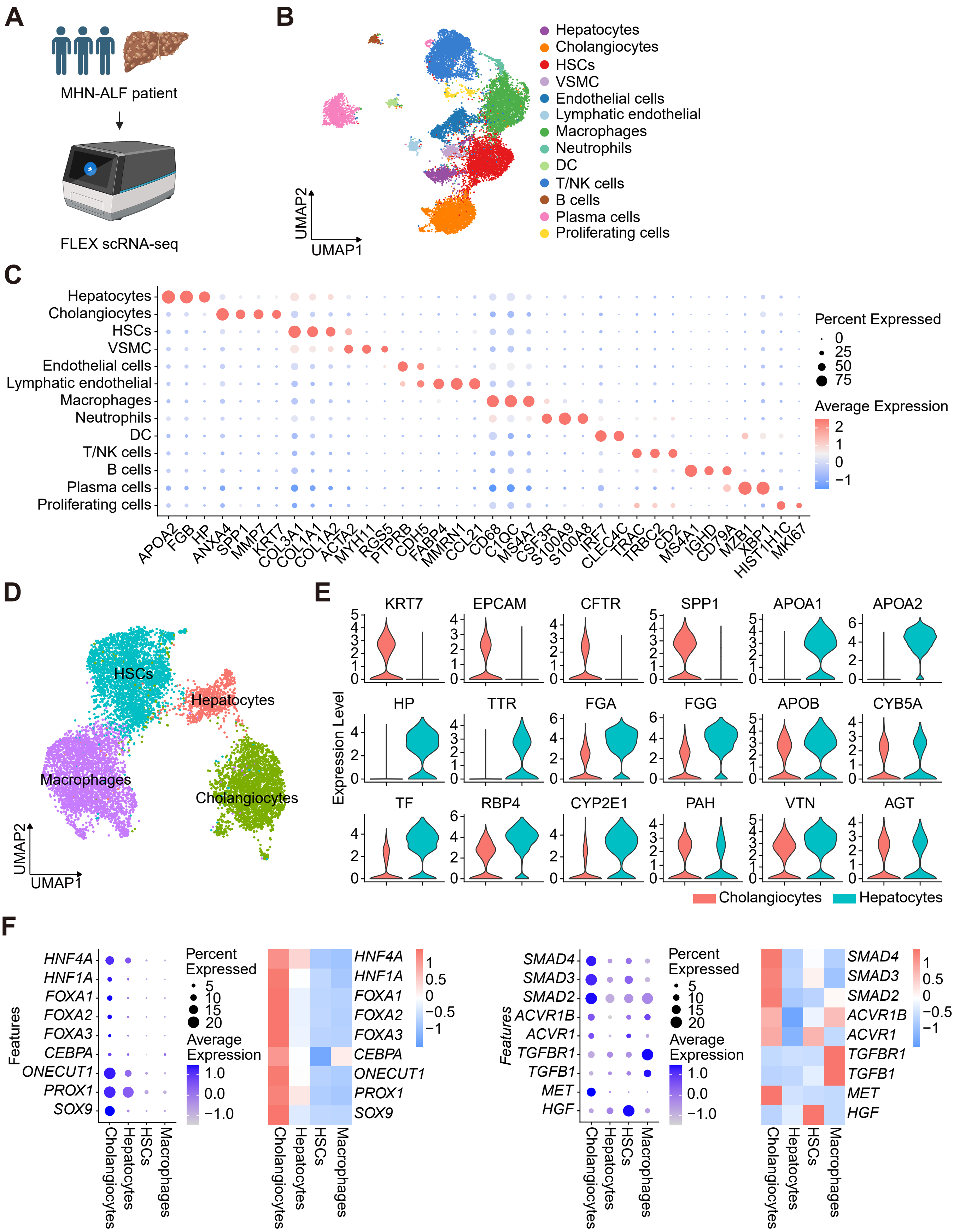
A
MHN-ALF patient
FLEX scRNA-seq
B
Hepatocytes
Cholangiocytes
HSCs
VSMC
Endothelial cells
Lymphatic endothelial
Macrophages
Neutrophils
DC
T/NK cells
B cells
Plasma cells
Proliferating cells
UMAP1
UMAP2
C
Percent Expressed
0
25
50
75
Average Expression
2
1
0
−1
APOA2
FGB
HP
ANXA4
SPP1
MMP7
KRT7
COL3A1
COL1A1
COL1A2
ACTA2
MYH11
RGS5
PTPRB
CDH5
FABP4
MMRN1
CCL21
CD68
C1QC
MS4A7
CSF3R
S100A9
S100A8
IRF7
CLEC4C
TRAC
TRBC2
CD2
MS4A1
IGHD
CD79A
MZB1
XBP1
HIST1H1C
MKI67
D
HSCs
Hepatocytes
Macrophages
Cholangiocytes
UMAP1
UMAP2
E
Expression Level
KRT7
EPCAM
CFTR
SPP1
APOA1
APOA2
HP
TTR
FGA
FGG
APOB
CYB5A
TF
RBP4
CYP2E1
PAH
VTN
AGT
Cholangiocytes
Hepatocytes
F
Features
HNF4A
HNF1A
FOXA1
FOXA2
FOXA3
CEBPA
ONECUT1
PROX1
SOX9
Percent Expressed
5
10
15
20
Average Expression
1.0
0.0
−1.0
Cholangiocytes
Hepatocytes
HSCs
Macrophages
1
0.5
0
−0.5
−1
SMAD4
SMAD3
SMAD2
ACVR1B
ACVR1
TGFBR1
TGFB1
MET
HGF

Figure S5

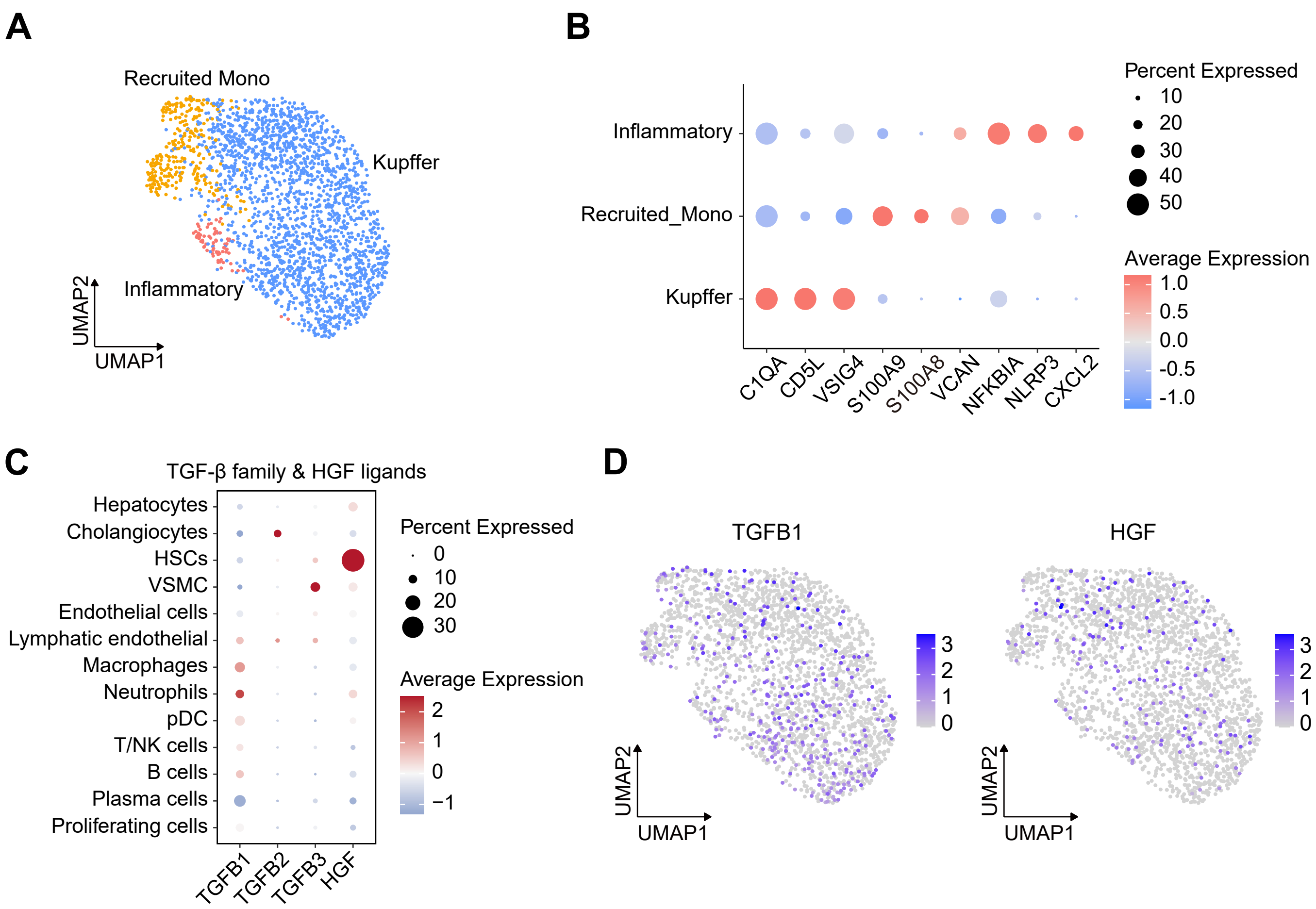
A
Recruited Mono
Kupffer
Inflammatory
UMAP2
UMAP1
B
Percent Expressed
10
20
30
40
50
Average Expression
1.0
0.5
0.0
-0.5
-1.0
Inflammatory
Recruited_Mono
Kupffer
C1QA
CD5L
VSIG4
S100A9
S100A8
VCAN
NFKBIA
NLRP3
CXCL2
C
TGF-β family & HGF ligands
Hepatocytes
Cholangiocytes
HSCs
VSMC
Endothelial cells
Lymphatic endothelial
Macrophages
Neutrophils
pDC
T/NK cells
B cells
Plasma cells
Proliferating cells
TGFB1
TGFB2
TGFB3
HGF
Percent Expressed
0
10
20
30
Average Expression
2
1
0
−1
D
TGFB1
HGF
3
2
1
0
UMAP2
UMAP1

Figure S6

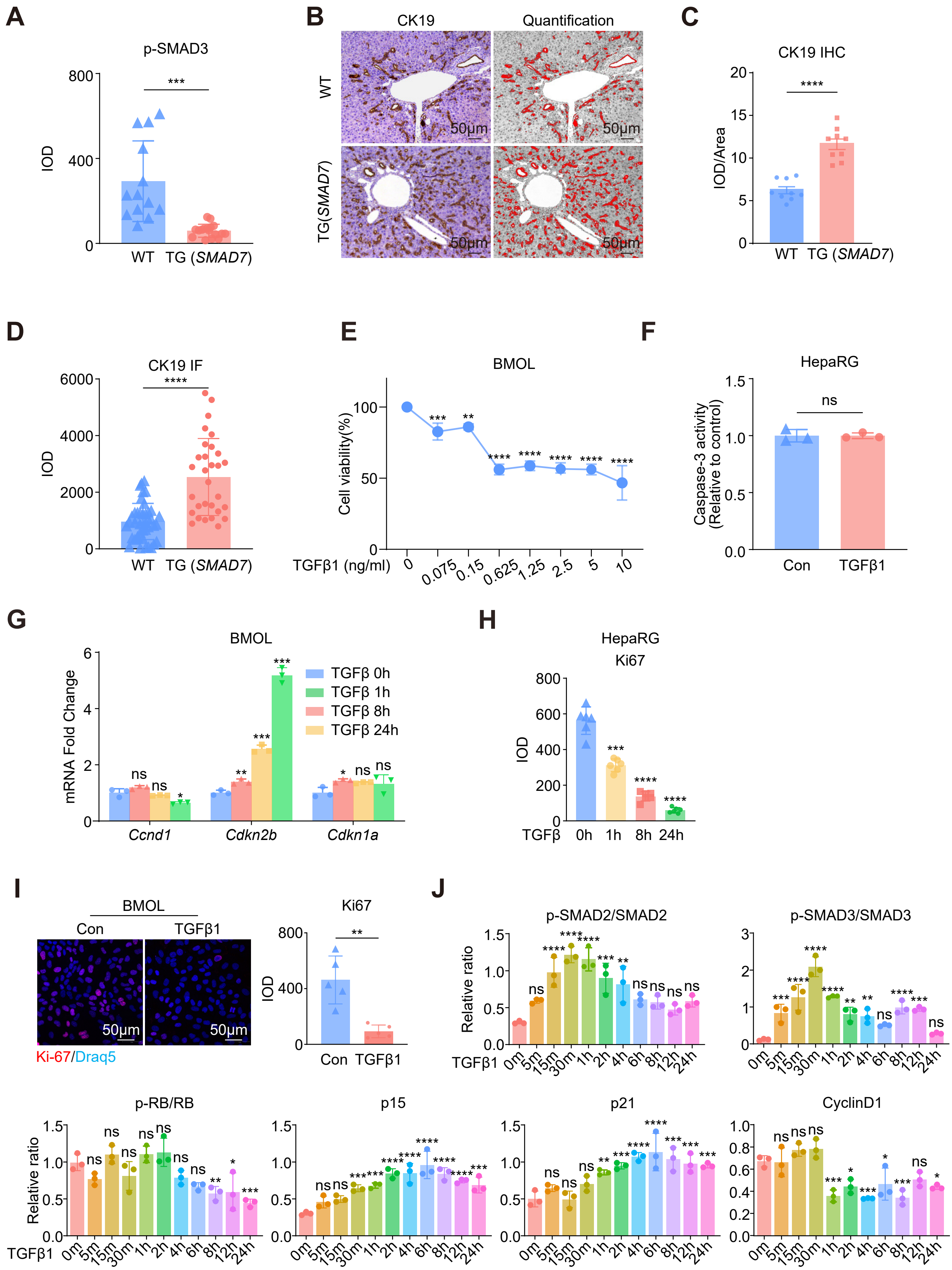

A
p-SMAD3
***
IOD
WT
TG (SMAD7)
B
CK19
Quantification
WT
TG(SMAD7)
50μm
C
CK19 IHC
****
IOD/Area
D
CK19 IF
****
E
BMOL
Cell viability(%)
TGFβ1 (ng/ml)
F
HepaRG
ns
Caspase-3 activity (Relative to control)
Con
TGFβ1
G
BMOL
mRNA Fold Change
TGFβ 0h
TGFβ 1h
TGFβ 8h
TGFβ 24h
Ccnd1
Cdkn2b
Cdkn1a
H
HepaRG
Ki67
TGFβ 0h 1h 8h 24h
I
BMOL
Con
TGFβ1
Ki-67/Draq5
Ki67
**
J
p-SMAD2/SMAD2
p-SMAD3/SMAD3
Relative ratio
p-RB/RB
p15
p21
CyclinD1
TGFβ1 0m 5m 15m 30m 1h 2h 4h 6h 8h 12h 24h

Figure S7

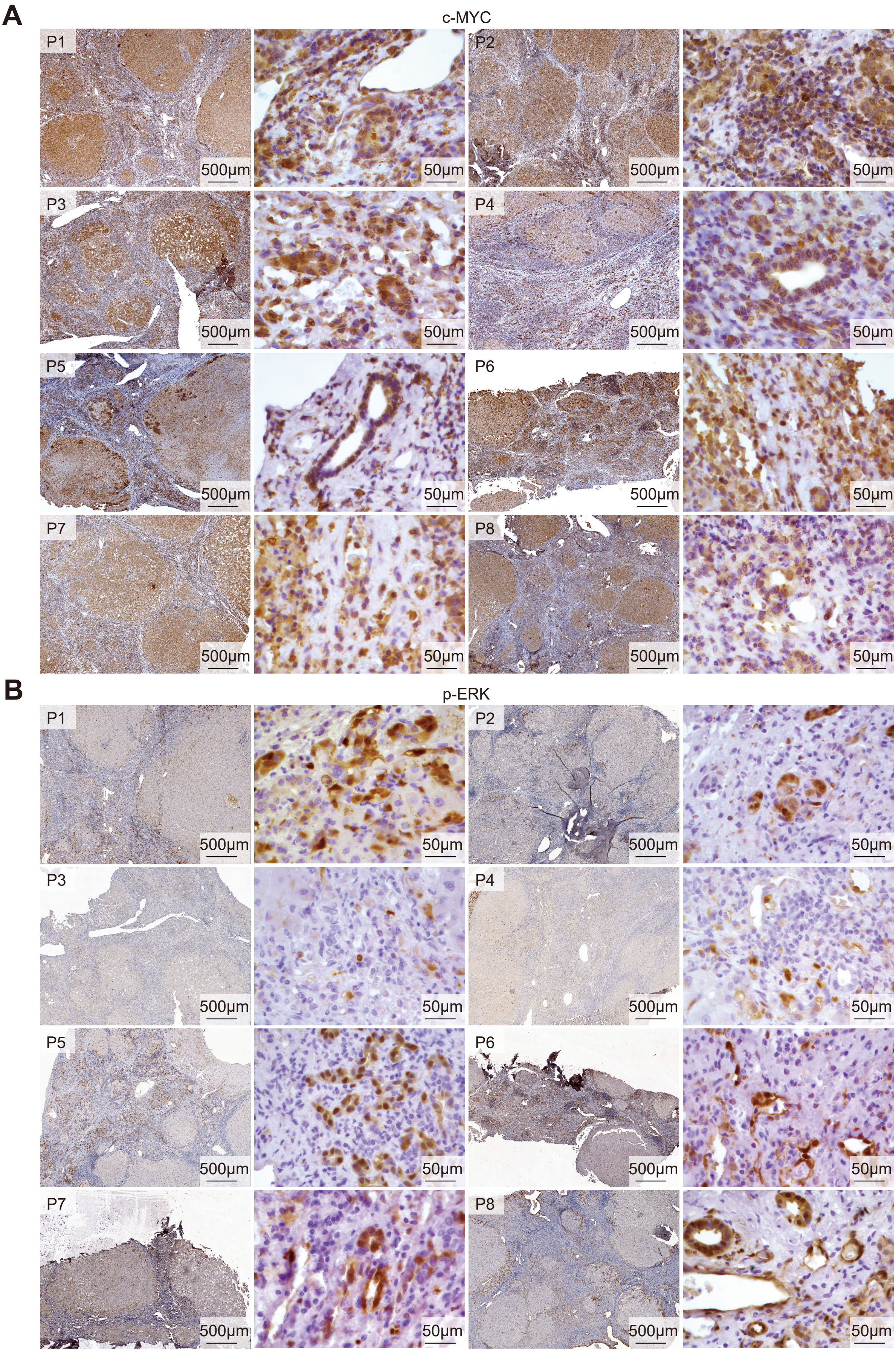
A
c-MYC
P1
500µm
50µm
P2
500µm
50µm
P3
500µm
50µm
P4
500µm
50µm
P5
500µm
50µm
P6
500µm
50µm
P7
500µm
50µm
P8
500µm
50µm
B
p-ERK
P1
500µm
50µm
P2
500µm
50µm
P3
500µm
50µm
P4
500µm
50µm
P5
500µm
50µm
P6
500µm
50µm
P7
500µm
50µm
P8
500µm
50µm

Figure S8

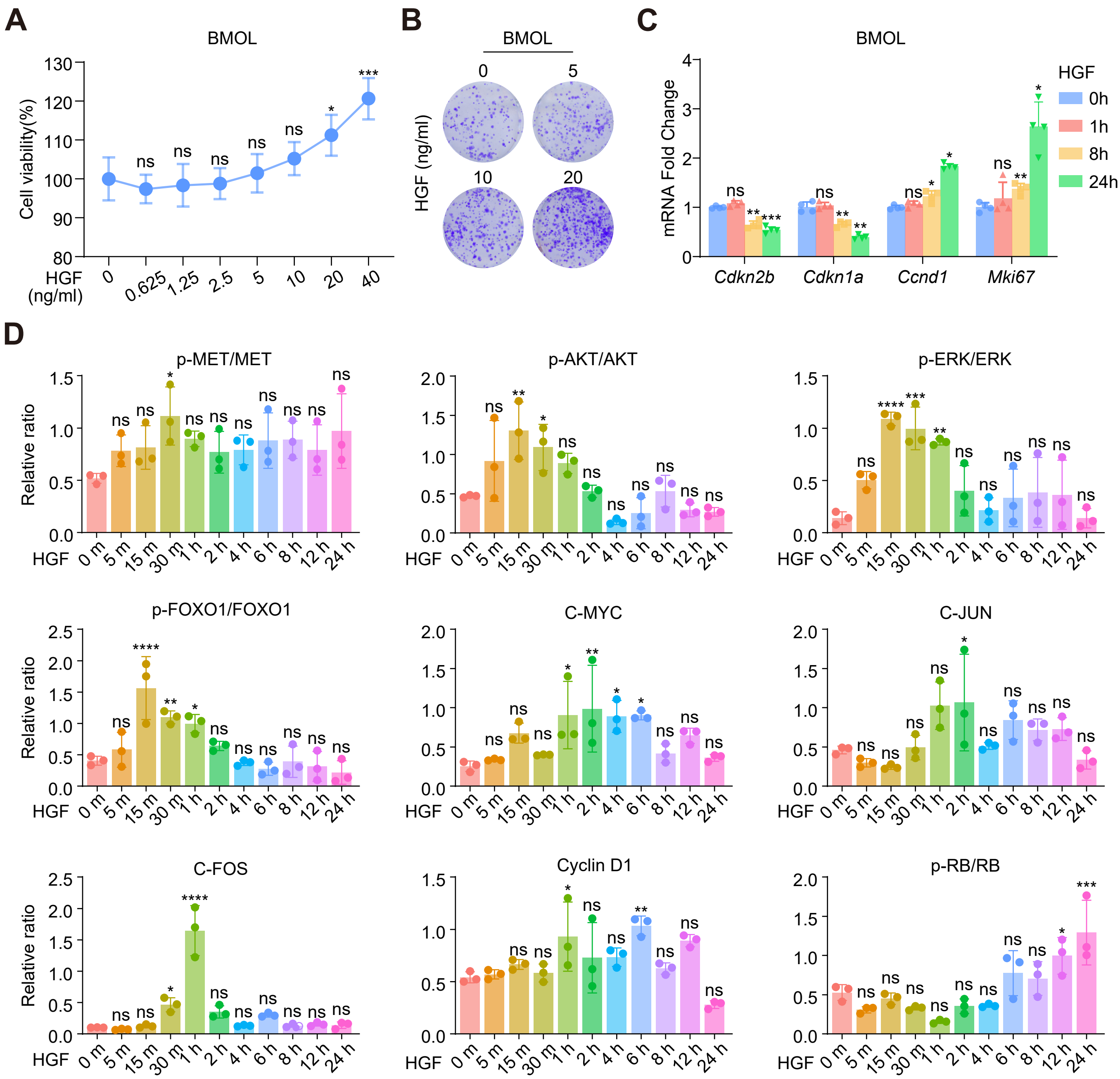

A
BMOL
Cell viability(%)
HGF (ng/ml)
0
0.625
1.25
2.5
5
10
20
40
B
BMOL
HGF (ng/ml)
0
5
10
20
C
BMOL
mRNA Fold Change
HGF
0h
1h
8h
24h
Cdkn2b
Cdkn1a
Ccnd1
Mki67
D
p-MET/MET
p-AKT/AKT
p-ERK/ERK
p-FOXO1/FOXO1
C-MYC
C-JUN
C-FOS
Cyclin D1
p-RB/RB
Relative ratio
HGF
0 m
5 m
15 m
30 m
1 h
2 h
4 h
6 h
8 h
12 h
24 h

Figure S9

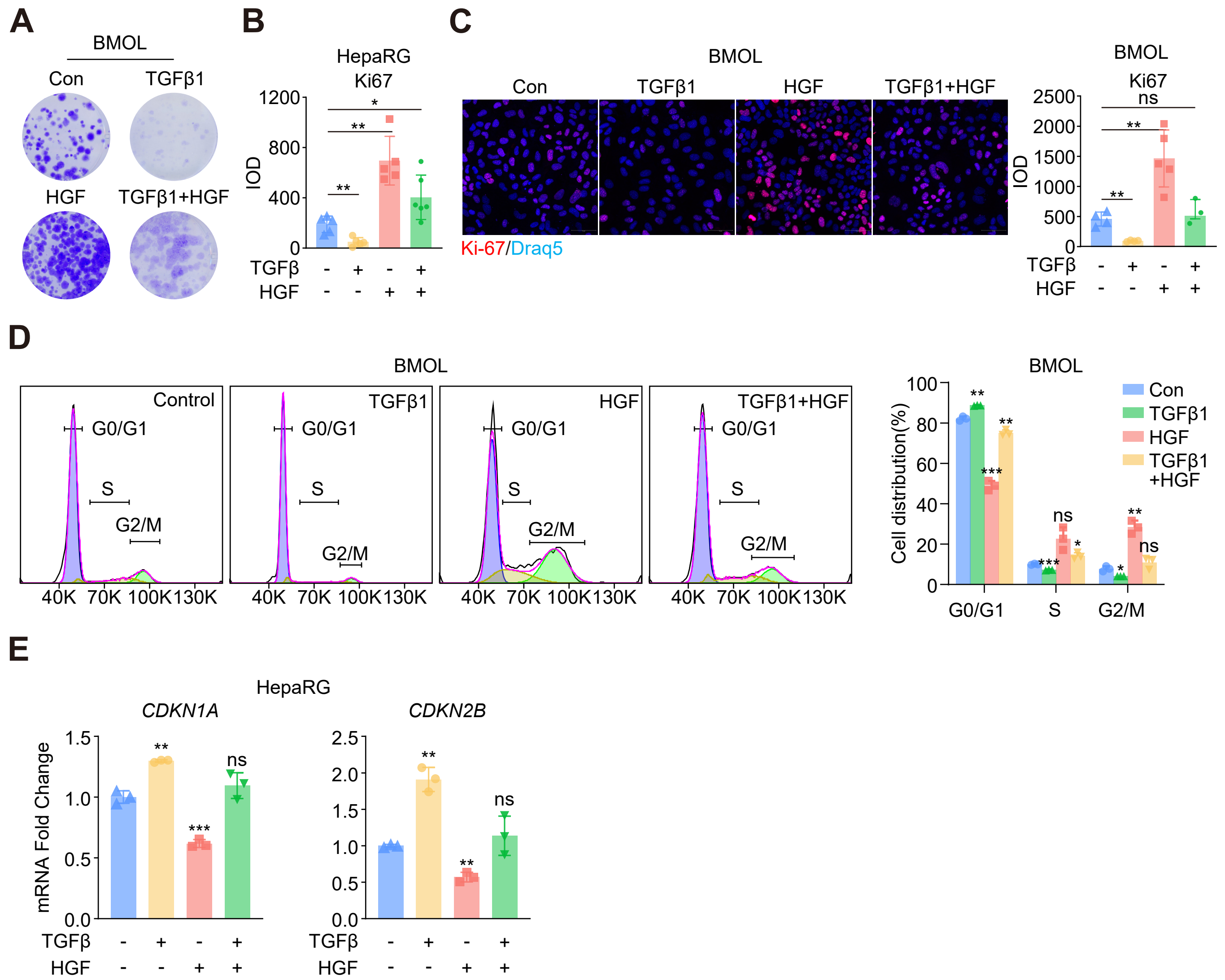
A
BMOL
Con
TGFβ1
HGF
TGFβ1+HGF
B
HepaRG
Ki67
IOD
TGFβ
HGF
C
BMOL
Con
TGFβ1
HGF
TGFβ1+HGF
Ki-67/Draq5
BMOL
Ki67
IOD
TGFβ
HGF
D
BMOL
Control
TGFβ1
HGF
TGFβ1+HGF
G0/G1
S
G2/M
40K 70K 100K130K
BMOL
Cell distribution(%)
Con
TGFβ1
HGF
TGFβ1 +HGF
G0/G1
S
G2/M
E
HepaRG
CDKN1A
CDKN2B
mRNA Fold Change
TGFβ
HGF

Figure S10

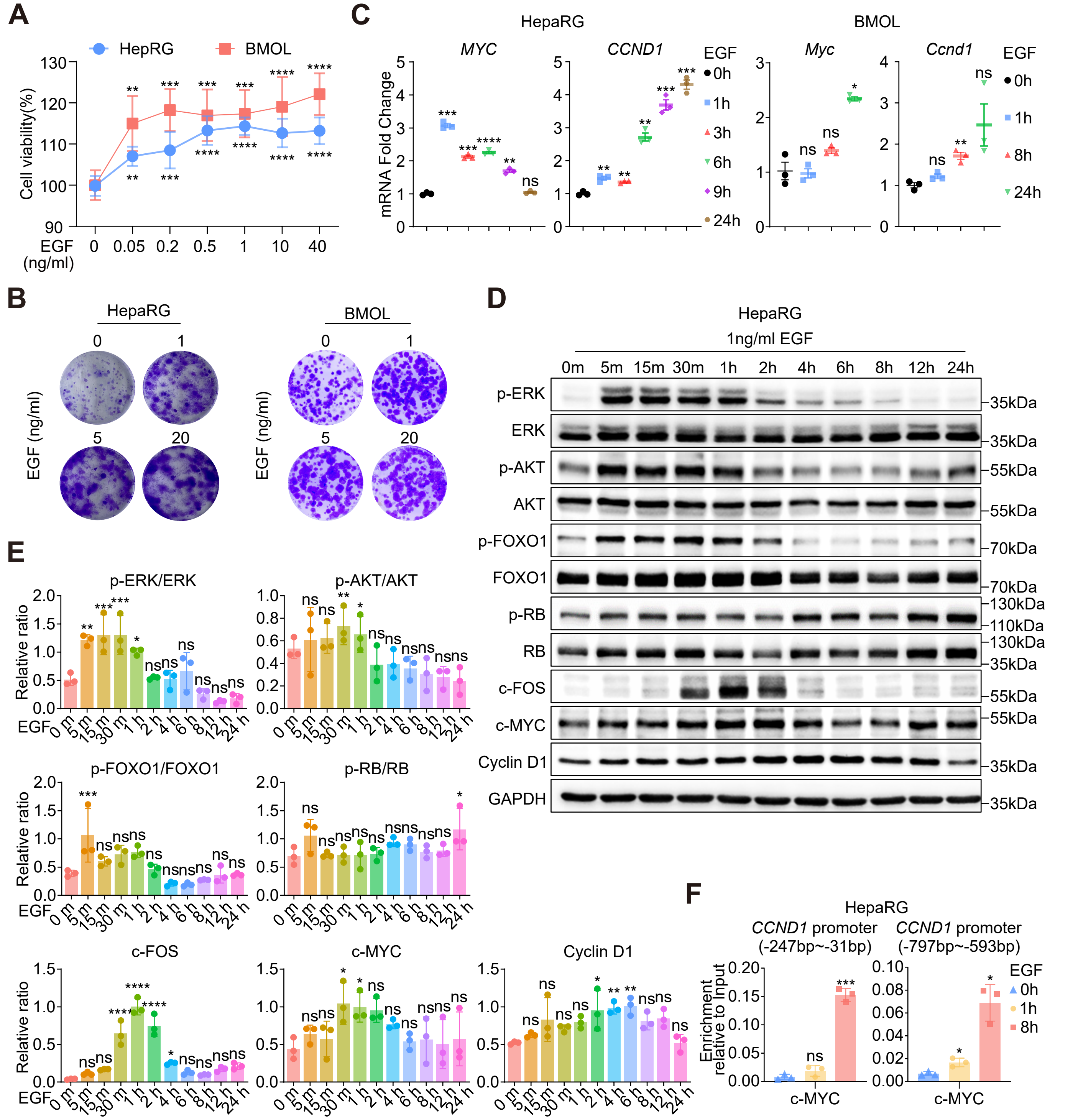

A
HepRG
BMOL
Cell viability(%)
EGF (ng/ml)
0 0.05 0.2 0.5 1 10 40
C
HepaRG
BMOL
MYC
CCND1
Myc
Ccnd1
mRNA Fold Change
EGF
0h
1h
3h
6h
9h
24h
8h
B
HepaRG
BMOL
EGF (ng/ml)
0 1 5 20
D
HepaRG
1ng/ml EGF
0m 5m 15m 30m 1h 2h 4h 6h 8h 12h 24h
p-ERK
ERK
p-AKT
AKT
p-FOXO1
FOXO1
p-RB
RB
c-FOS
c-MYC
Cyclin D1
GAPDH
35kDa
55kDa
70kDa
130kDa
110kDa
E
p-ERK/ERK
p-AKT/AKT
p-FOXO1/FOXO1
p-RB/RB
c-FOS
c-MYC
Cyclin D1
Relative ratio
EGF
0 m 5 m 15 m 30 m 1 h 2 h 4 h 6 h 8 h 12 h 24 h
F
HepaRG
CCND1 promoter (-247bp~-31bp)
CCND1 promoter (-797bp~-593bp)
Enrichment relative to Input
c-MYC
EGF
0h
1h
8h

Figure S11

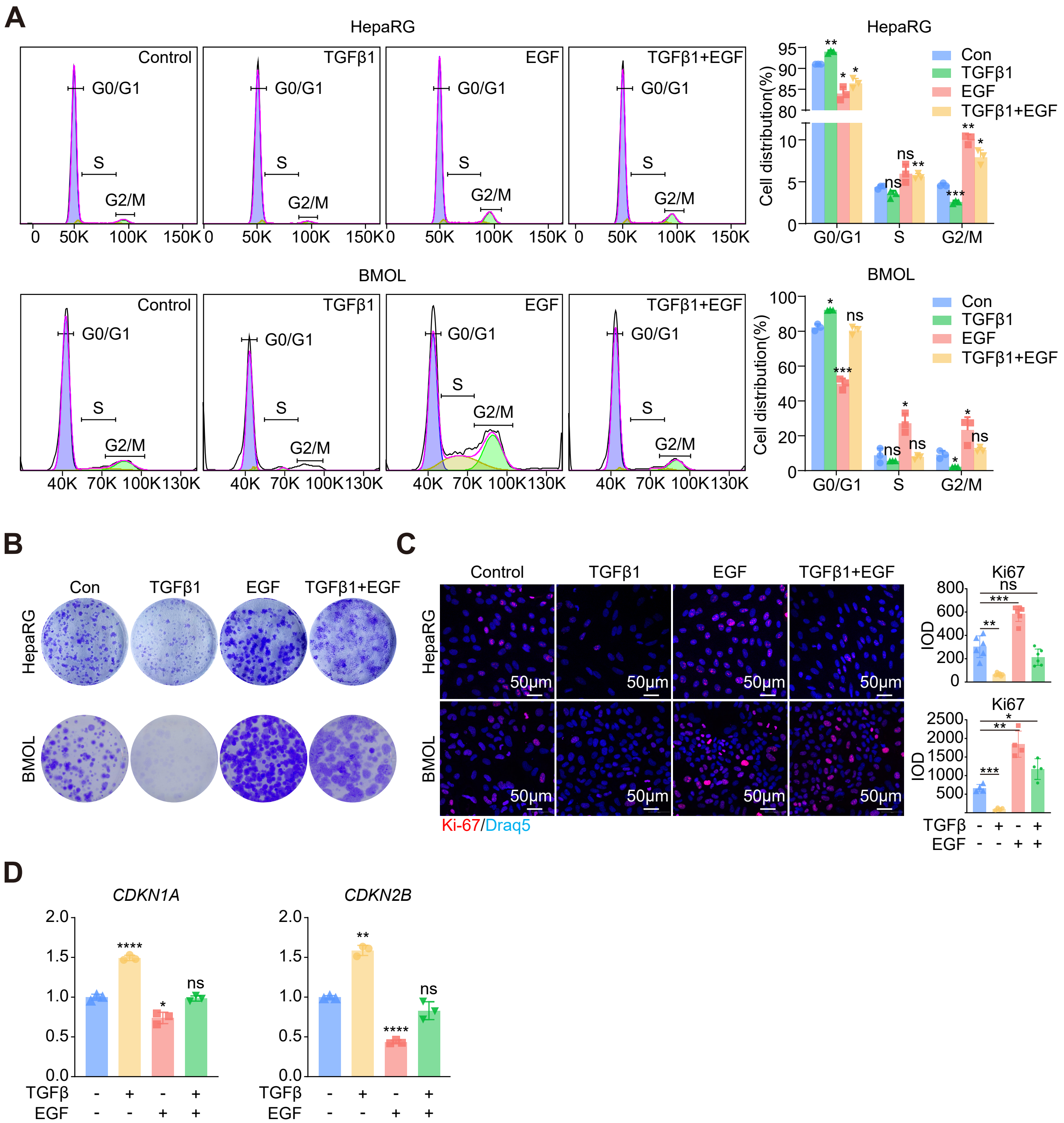
A
HepaRG
Control
TGFβ1
EGF
TGFβ1+EGF
G0/G1
S
G2/M
0 50K 100K 150K
HepaRG
Con
TGFβ1
EGF
TGFβ1+EGF
Cell distribution(%)
G0/G1 S G2/M
BMOL
40K 70K 100K 130K
BMOL
B
Con TGFβ1 EGF TGFβ1+EGF
HepaRG
BMOL
C
Control TGFβ1 EGF TGFβ1+EGF
50μm
Ki-67/Draq5
Ki67
IOD
TGFβ
EGF
D
CDKN1A
CDKN2B
TGFβ
EGF

Figure S12

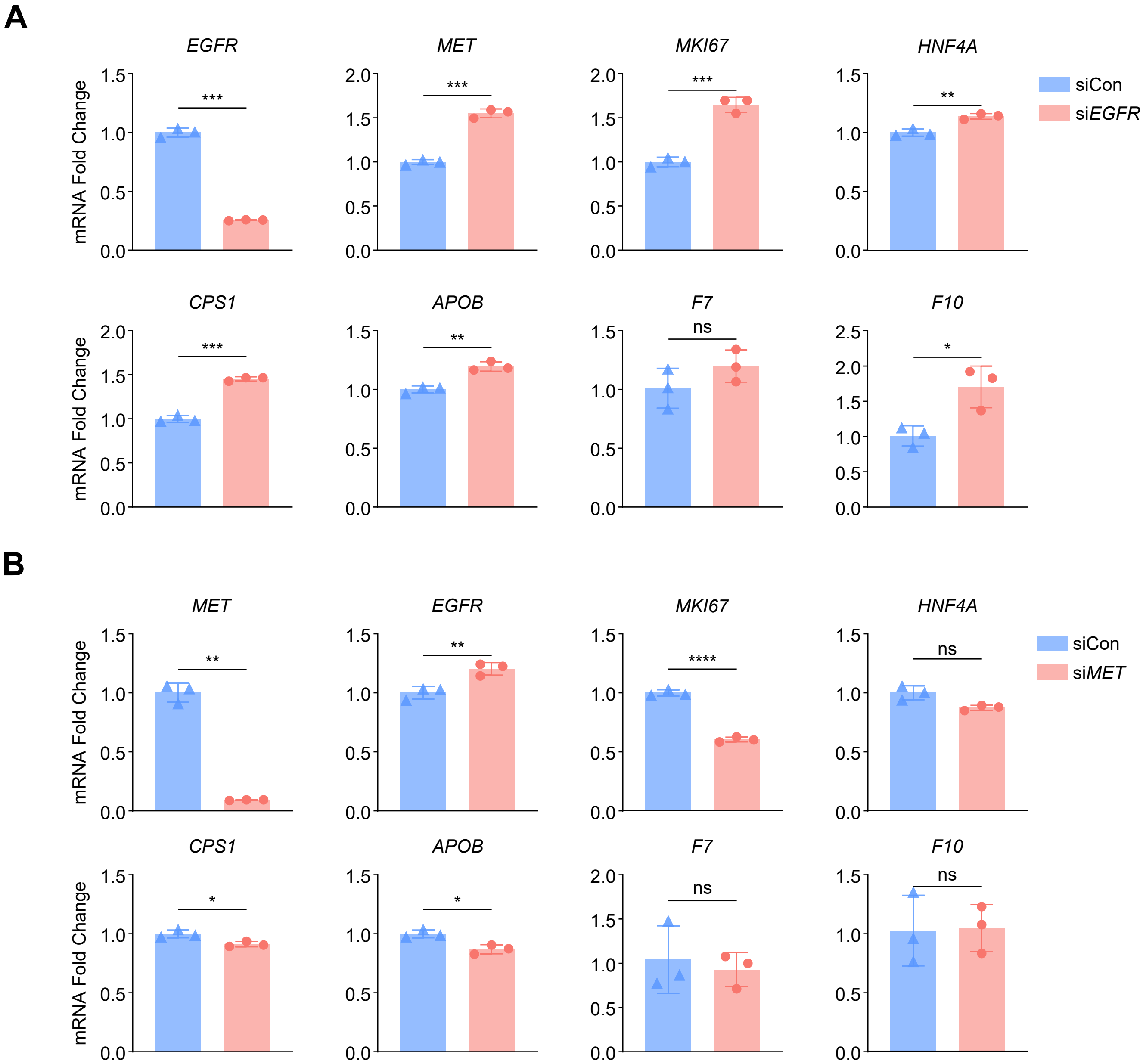
A
EGFR
MET
MKI67
HNF4A
siCon
siEGFR
mRNA Fold Change
***
***
***
**
CPS1
APOB
F7
F10
***
**
ns
*
B
MET
EGFR
MKI67
HNF4A
siCon
siMET
**
**
****
ns
CPS1
APOB
F7
F10
*
*
ns
ns

Figure S13

A

DMSO

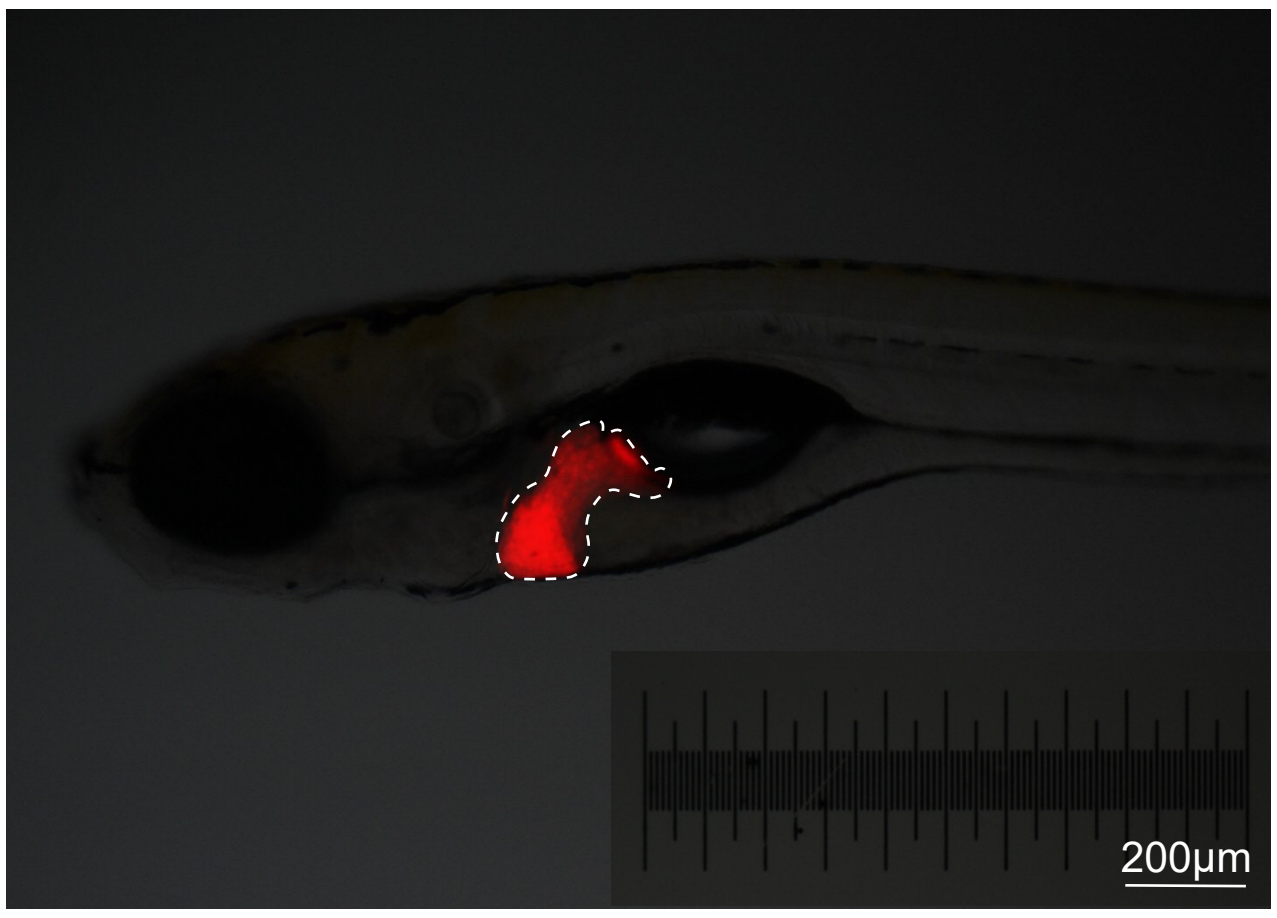

Supplement: Supplementary file 1 [file jci-136-199913-s243.pdf]
