# Supplementary material for: TGF-β serves as a critical signaling determinant of liver progenitor cell activation and function
Source: J Clin Invest. 2026 Jul 28;136(18):e199913. doi: 10.1172/JCI199913 (PMC13574154; doi:10.1172/JCI199913)
Supplement: Supplementary file 2 [file jci-136-199913-s244.pdf]

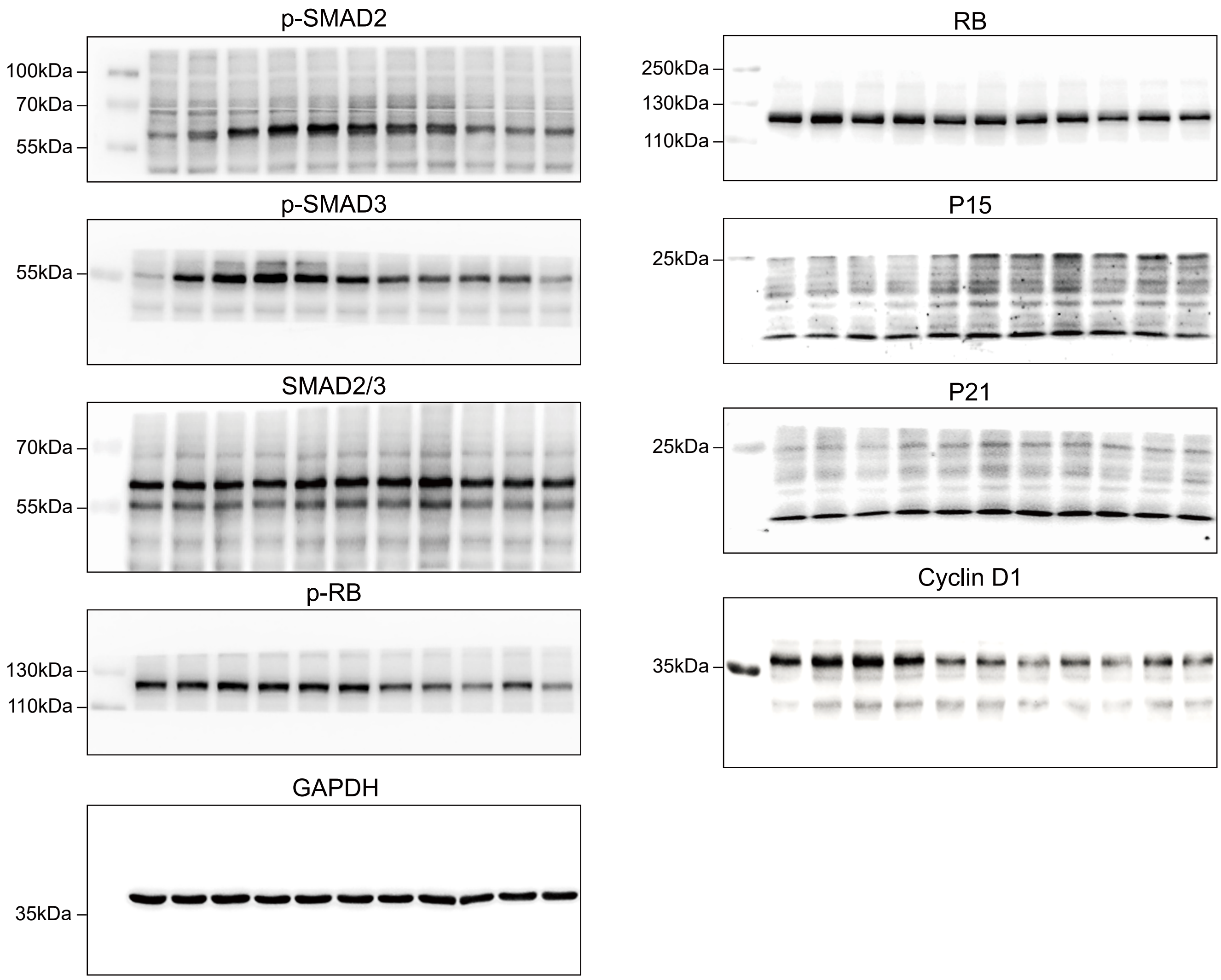

Figure 1. Uncropped Western blots for Figure 4H. Full-length, uncropped images of p-SMAD2, p-SMAD3, SMAD2/3, p-RB, RB, P15, P21, Cyclin D1, and GAPDH corresponding to Figure 4H are shown. Molecular weight markers are indicated.

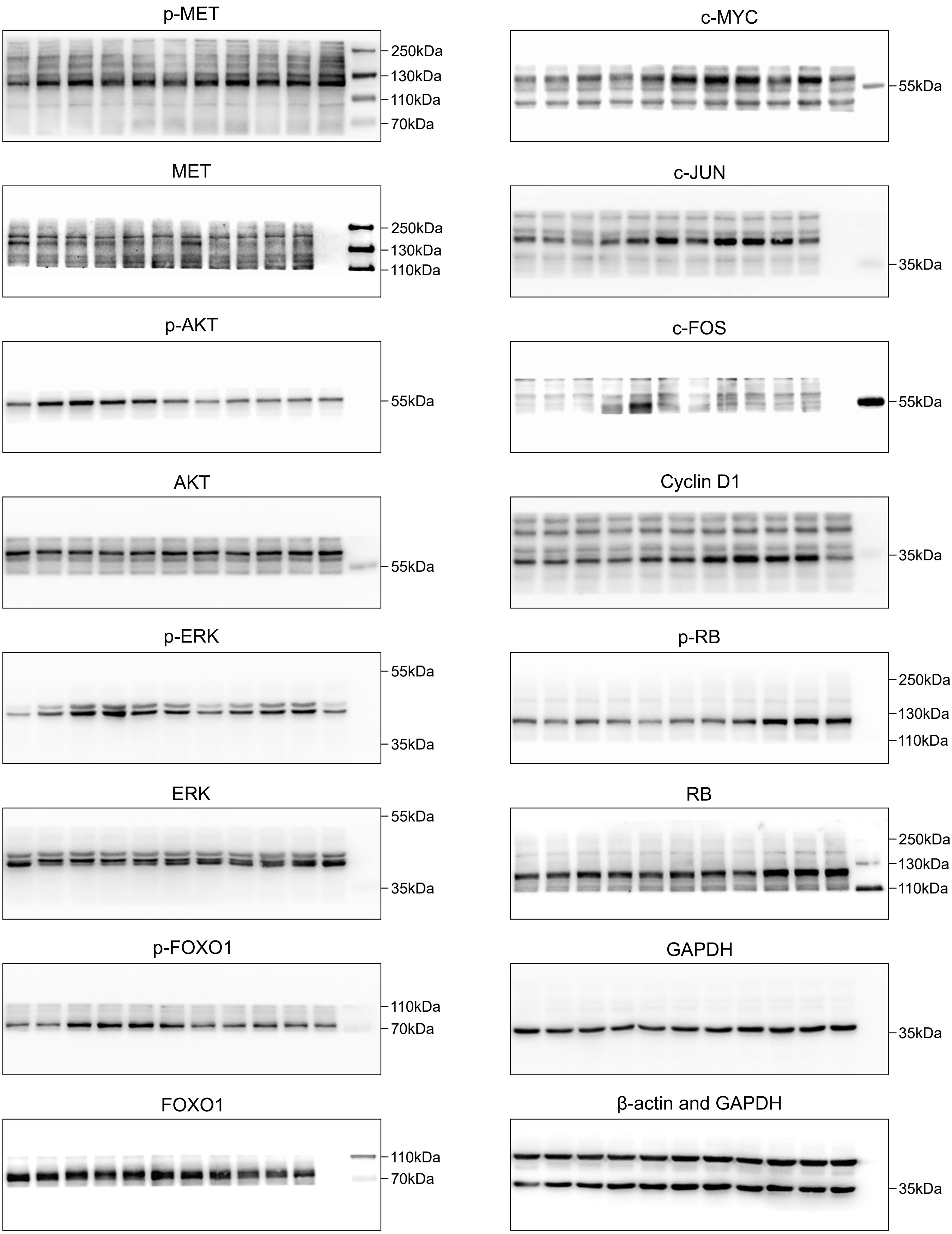

Figure 2. Uncropped Western blots for Figure 6E–F. Full-length, uncropped images of p-MET, MET, p-AKT, AKT, p-ERK, ERK, p-FOXO1, FOXO1, c-MYC, c-JUN, c-FOS, Cyclin D1, p-RB, RB, GAPDH, and β-actin corresponding to Figure 6E–F are shown. Molecular weight markers are indicated.

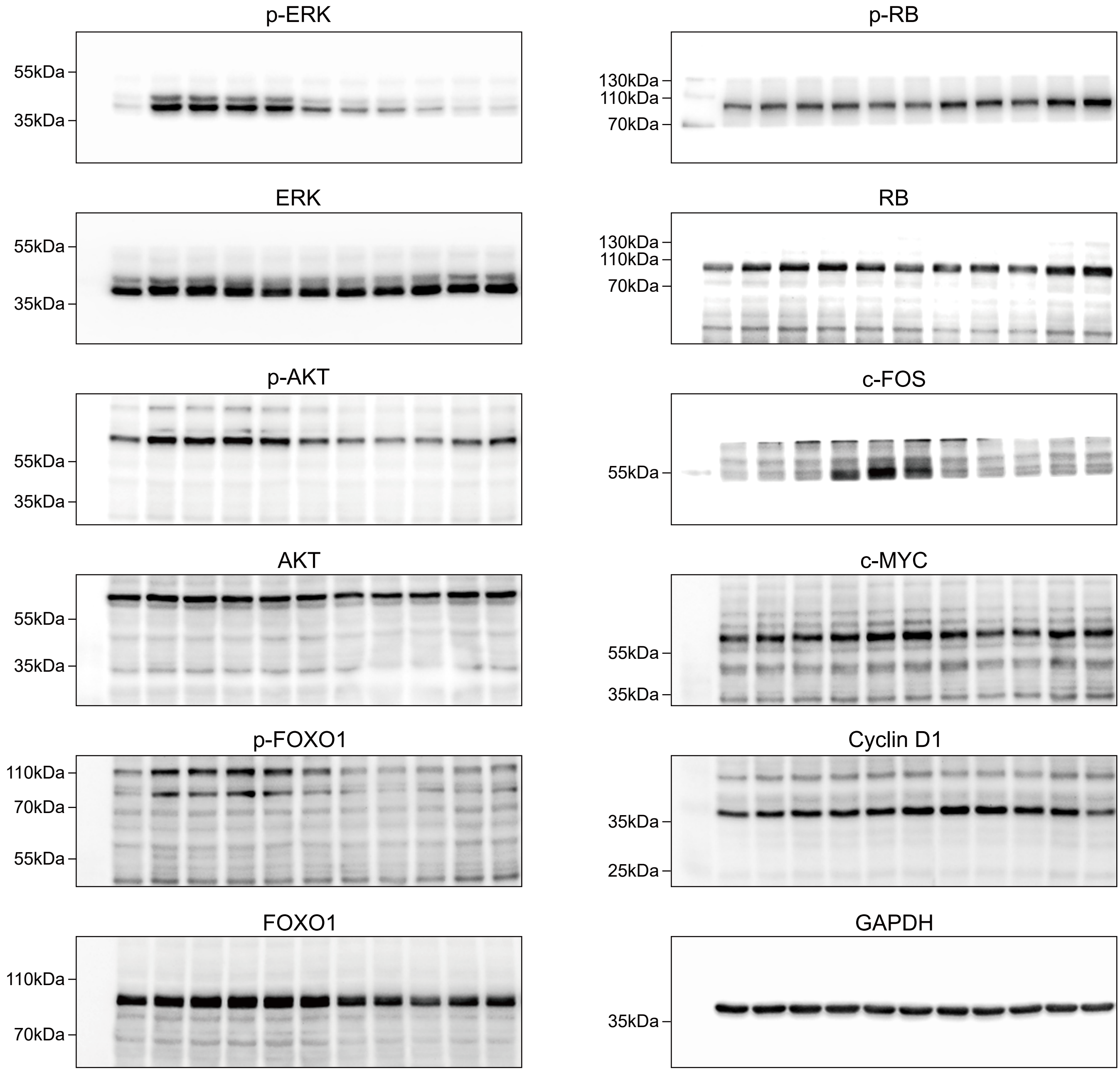

Figure 3. Uncropped Western blots for Figure S10D. Full-length, uncropped images of p-ERK, ERK, p-AKT, AKT, p-FOXO1, FOXO1, p-RB, RB, c-FOS, c-MYC, Cyclin D1, and GAPDH corresponding to Figure S9D are shown. Molecular weight markers are indicated.
